# Supplementary figures and images for: CRISPR/Cas with ribonucleoprotein complexes and transiently selected telomere vectors allows highly efficient marker-free and multiple genome editing in Botrytis cinerea
Source: PLoS Pathog. 2020 Aug 17;16(8):e1008326. doi: 10.1371/journal.ppat.1008326 (PMC7451986; doi:10.1371/journal.ppat.1008326)

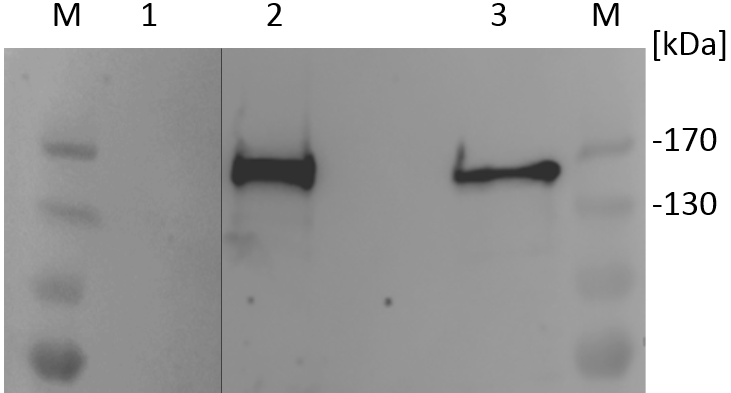

Supplement: S1 Fig — Total B. cinerea protein extracts (15 μg per lane) were loaded, separated by polyacrylamide gel electrophoresis, and Cas9 detected with a monoclonal Cas9 antibody. M: Marker; 1: B05.10 (WT); 2: B05.10-Cas9-SV40x4 (stably integrated gene); 3: B05.10 (pTEL-Cas9-Stux2). (TIF) [file ppat.1008326.s001.tif]

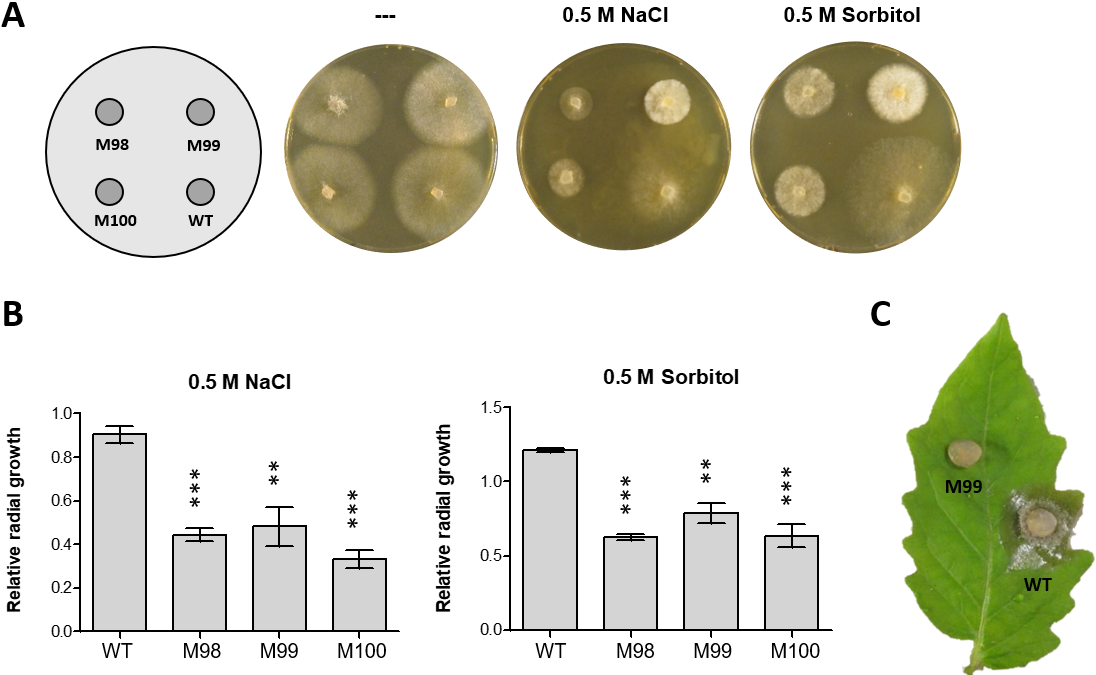

Supplement: S2 Fig — (A) Pictures of three IprR Bos1 mutants (M98, M99, M100, all having the same ‘+T’ insertion) and WT growth for 48 h on ME medium without (—) and with 0.5 M NaCl or sorbitol. (B) Effects of salt and osmotic stress treatments on radial growth, compared to growth on pure ME medium (n = 3). The p values by one-way ANOVA followed by Dunnett’s multiple comparisons (control: WT) post hoc test are indicated. **p ≤ 0.01; ***p ≤ 0.001 (n = 4). (C) Infection on tomato leaf by WT and mutant M99 (72 h). (TIF) [file ppat.1008326.s002.tif]

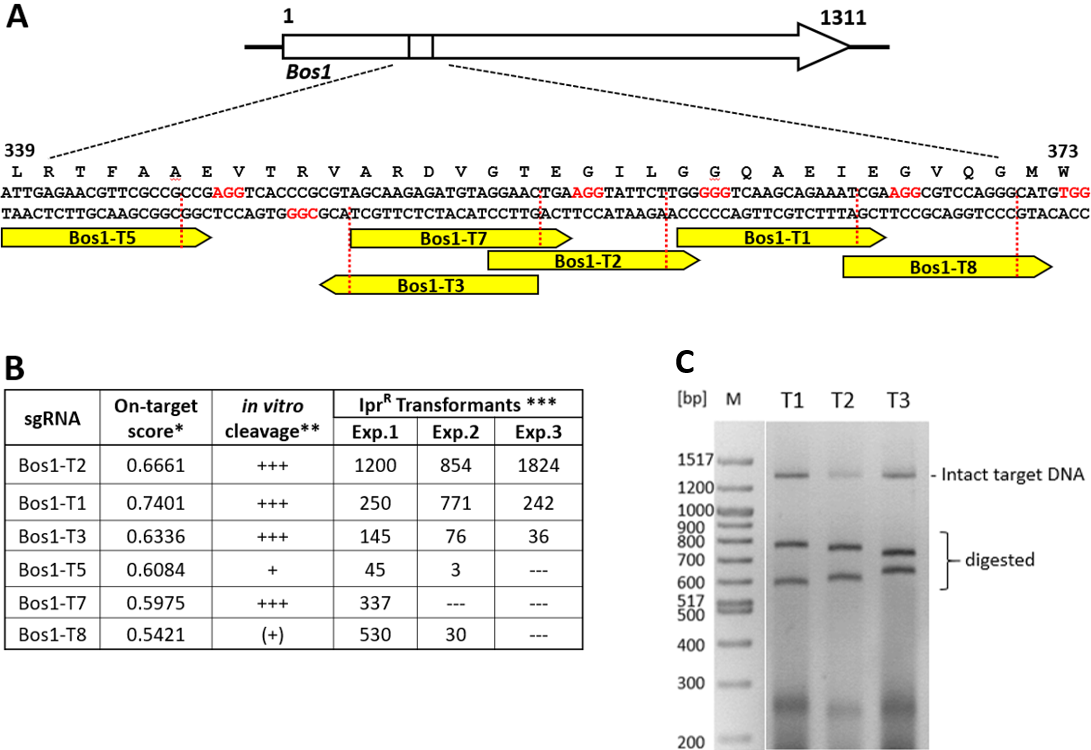

Supplement: S3 Fig — (A) Positions and expected cleavage sites (red dotted lines) of the sgRNAs (yellow) in Bos1. PAM sequences for each of the sgRNAs are indicated in red. (B) Summary of on-target scores, in vitro cleavage activities, and transformation efficiencies with different sgRNAs. *On-target efficiency scores calculated with the Broad Institute GPP sgRNA Designer. **In vitro cleavage efficiency was estimated from gel pictures. *** Number of IprR B. cinerea transformants per assay. (C) Examples of in vitro cleavage reactions with sgRNAs bos1-T1, -T2 and -T3. (TIF) [file ppat.1008326.s003.tif]

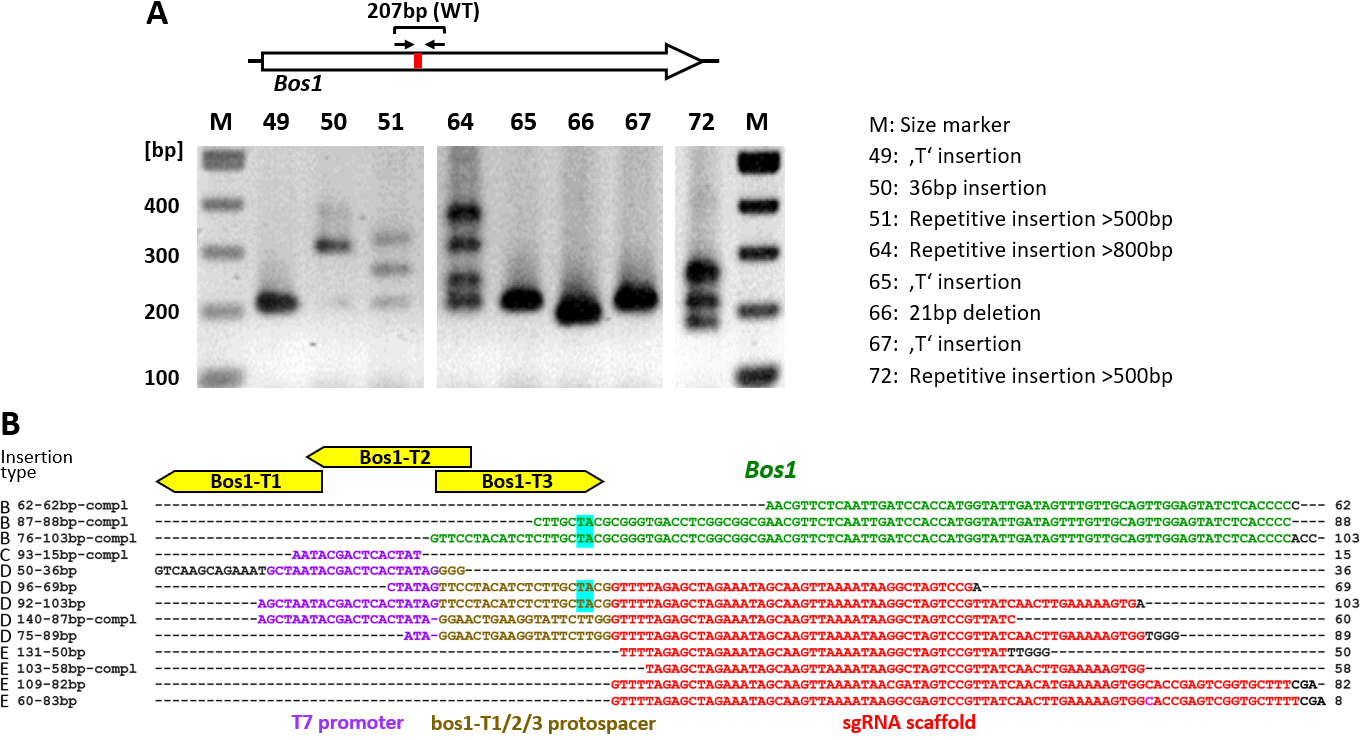

Supplement: S4 Fig — (A) Ethidium bromide-stained agarose gels (negative pictures) with PCR fragments generated with primers TL_87 Bos1_check 200 Fw/ TL_87 Bos1_check 200 Rv, showing variations of fragment sizes due to different types of NHEJ-induced mutations in transformants obtained with Cas9/bos1-T1 RNP. (B) Origins and sequences of different types of NHEJ insertions obtained with Cas9/bos1-T1, Cas9/bos1-T2 and Cas9/bos1-T3 RNPs. Type A (not shown): 164 bp B. cinerea mitochondrial DNA, two joined fragments of 84 and 79 bp. Type B: B. cinerea Bos1-DNA. Type C: 15 bp fragment of the sgRNA scaffold encoding part of the T7 RNA polymerase promoter. Type D: sgRNA scaffold DNA containing part or all of the protospacer sequences of bos1-T1/-2/-3. Type E: sgRNA scaffold DNA lacking protospacer sequences. Positions of the sgRNAs relative to the Bos1 sequence (in green) are shown. compl: Insertion in inverse orientation, complementary sequence is shown. The bases flanking the Bos1-T3 sgRNA cleavage site are indicated with a blue background. (TIF) [file ppat.1008326.s004.tif]

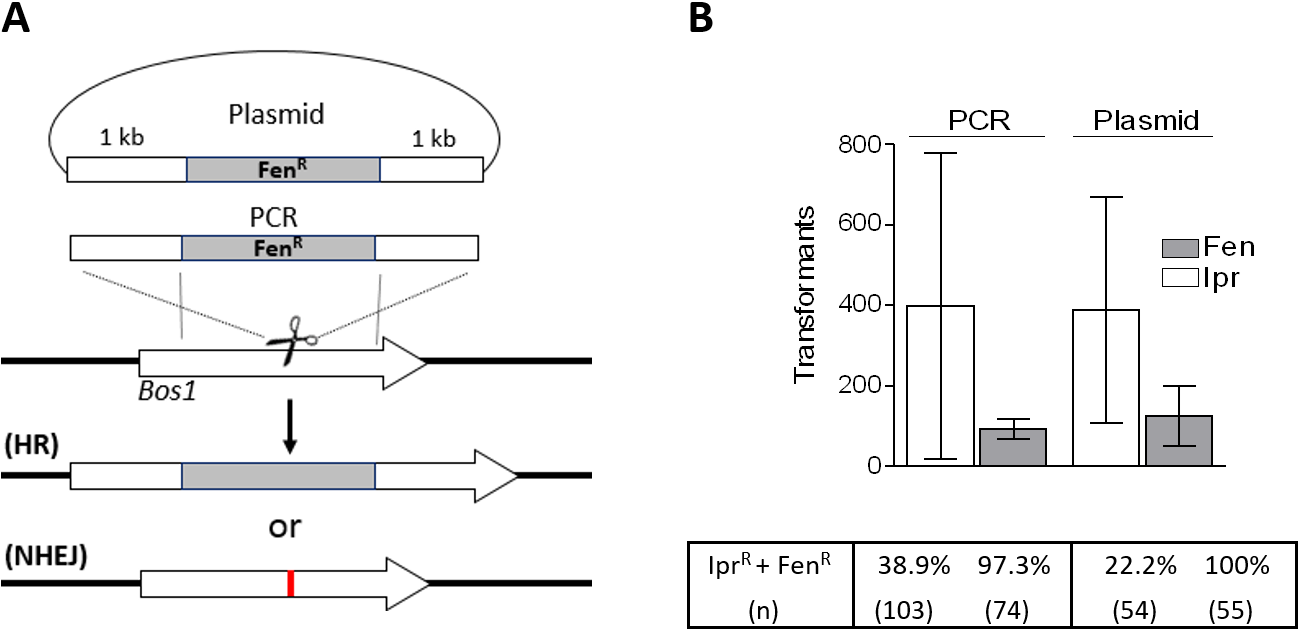

Supplement: S5 Fig — (A) Experimental scheme. Bos1 inactivation leading to IprR occurs either by targeted integration of the FenR RT via HR, or via NHEJ. As RT, either a circular plasmid or a PCR fragment amplified from this plasmid, both containing 1 kb Bos1 homology flanks, were used. (B) Transformation results: Primary selection was either for IprR (white bars) or for FenR (grey bars) (n = 3). When the PCR fragment or the plasmid were transformed without RNP as control, no FenR colonies, except for one colony in one experiment, were obtained. Below the diagram, the fraction of transformants with resistance to both fungicides is shown. (n): Number of transformants tested. Statistical analyses were performed by analysis of variance (ANOVA, followed by Dunnett’s multiple comparisons. No significant differences between transformation results with PCR fragments and circular plasmids, or between the numbers of IprR and FenR colonies obtained with the same batches of transformed protoplasts were observed. (TIF) [file ppat.1008326.s005.tif]

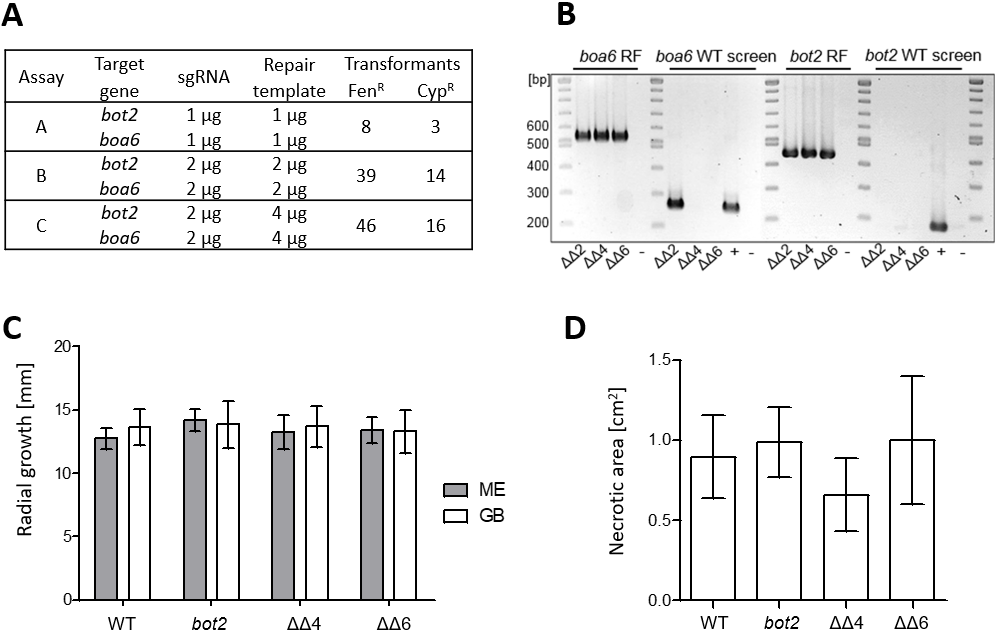

Supplement: S6 Fig — (A) Transformation results. (B) PCR-based verification of bot2 boa6 double (ΔΔ) k.o. mutants. boa6 RT right flank (RF) integration screen using primers TniaD_ol_Cyp_Fw/ TL129 (537 bp); boa6 WT screen using primers TL157/ TL158 (263 bp), bot2 RT RF integration screen using primers TL130/ TL132 (444 bp), bot2 WT screen using primers TL133/ TL159 (180 bp). +: B. cinerea WT DNA (positive control); -: no template DNA (negative control). (C) Growth of WT and mutants after 72 h on agar plates with rich (ME) and minimal (GB: Gamborg B5 with 25 mM glucose) medium (one-way ANOVA; n = 3). (D) Lesion formation after 72 h on tomato leaves (one way ANOVA; n = 3). In (C) and (D), no significant differences in radial growth and infection between WT and mutants were observed. (TIF) [file ppat.1008326.s006.tif]

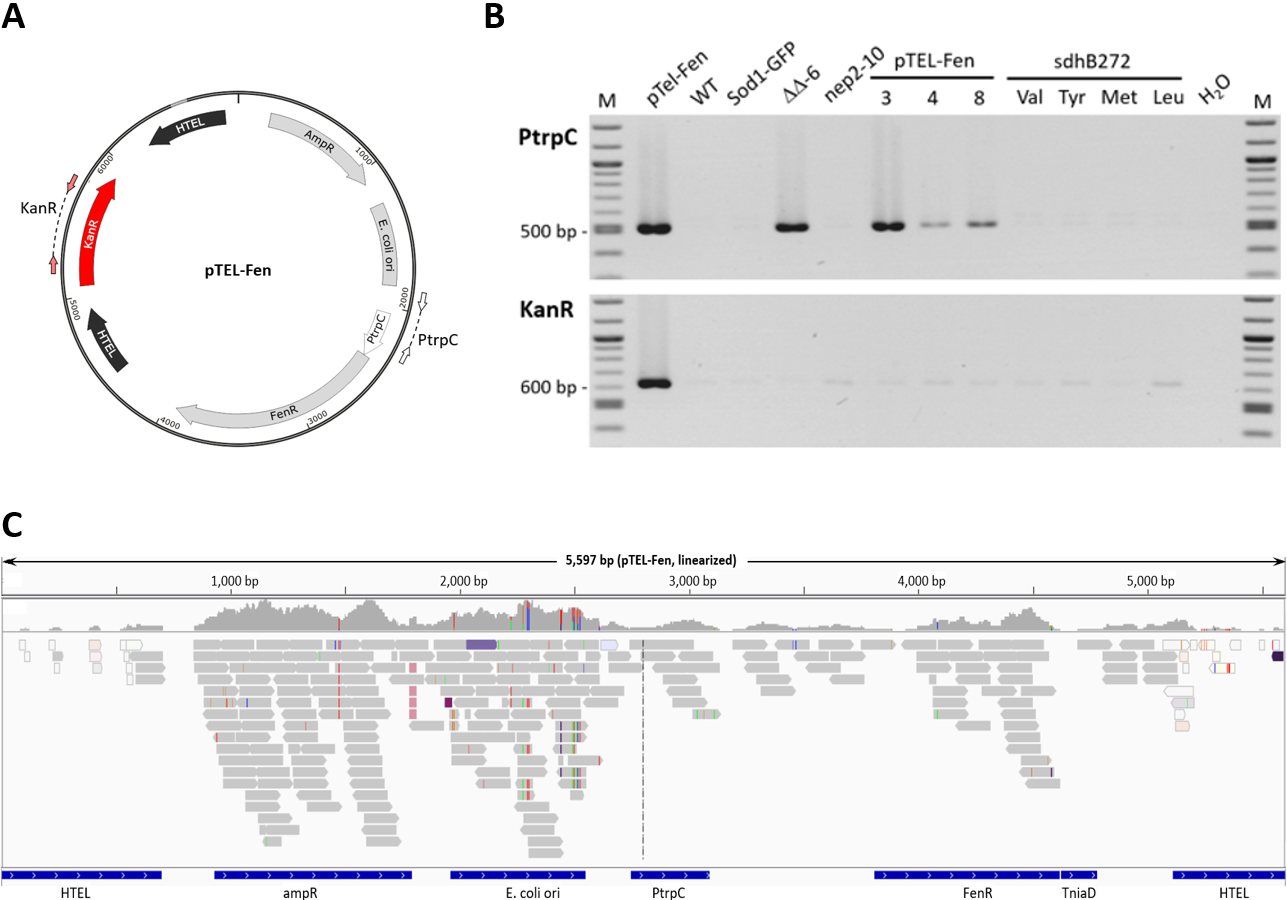

Supplement: S7 Fig — (A) Map of pTEL-Fen, indicating the PCR fragments that were amplified. (B) Negative picture of ethidium bromide stained agarose gel showing the result of the PCR reactions performed with total DNA from the B. cinerea strains used for sequencing. Expected sizes were for kanR: 625 bp; PtrpC: 504 bp. (C) Alignment of Illumina bam reads mapping to the linearized sequence of pTEL-Fen in the DNA of B. cinerea strain pTEL-Fen8. (TIF) [file ppat.1008326.s007.tif]

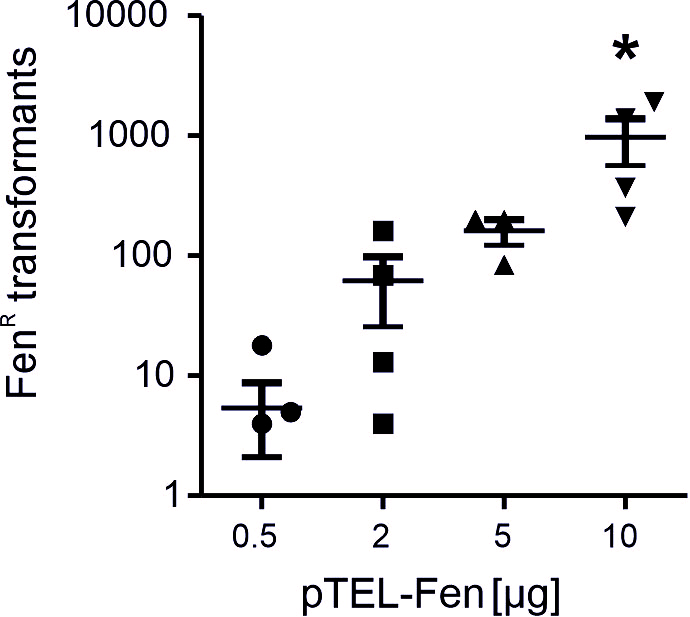

Supplement: S8 Fig — Individual data points are shown. The p value by one-way ANOVA followed by Dunnett’s multiple comparisons (control: 0.5 μg pTEL-Fen) post hoc test is indicated. *p ≤ 0.05. (TIF) [file ppat.1008326.s008.tif]

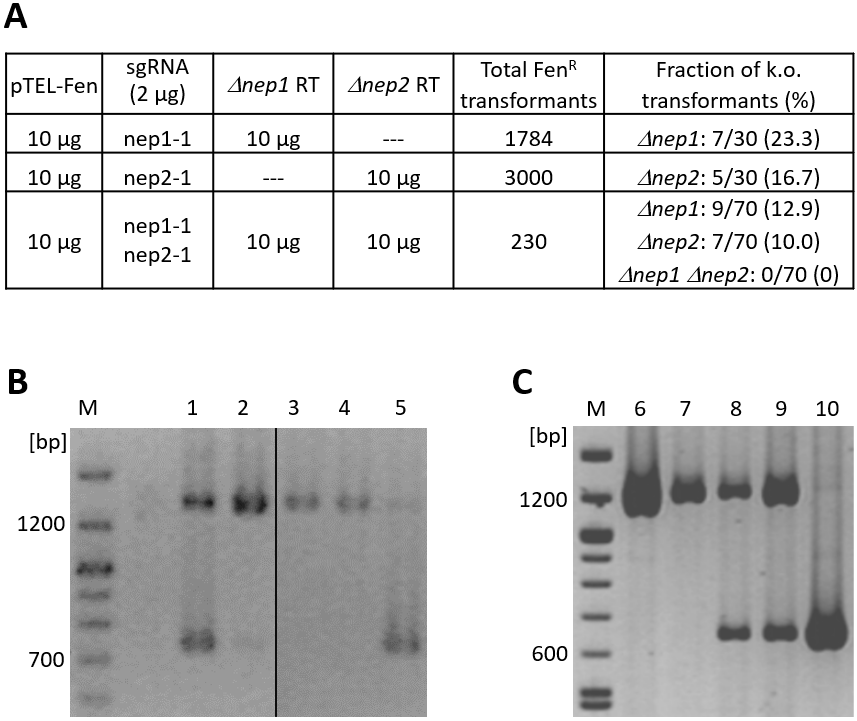

Supplement: S9 Fig — (A) Transformation result. (B) PCR-based verification of nep1 deletion mutants, using primers TL143/ TL144; size of WT fragment 1,353 bp, size of nep1 k.o. fragment 733 bp. Lanes 1–5: FenR transformants. Transformant #5 represents a nearly pure nep1 mutant. (C) PCR-based verification of nep2 deletion mutants, using primers TL145/ TL146; size of WT fragment 1,220 bp, size of nep2 k.o. fragment 641 bp. Lane 6: B. cinerea WT; lanes 7–10: FenR transformants. Transformant #10 represents a purified nep2 mutant. M: DNA marker. (TIF) [file ppat.1008326.s009.tif]

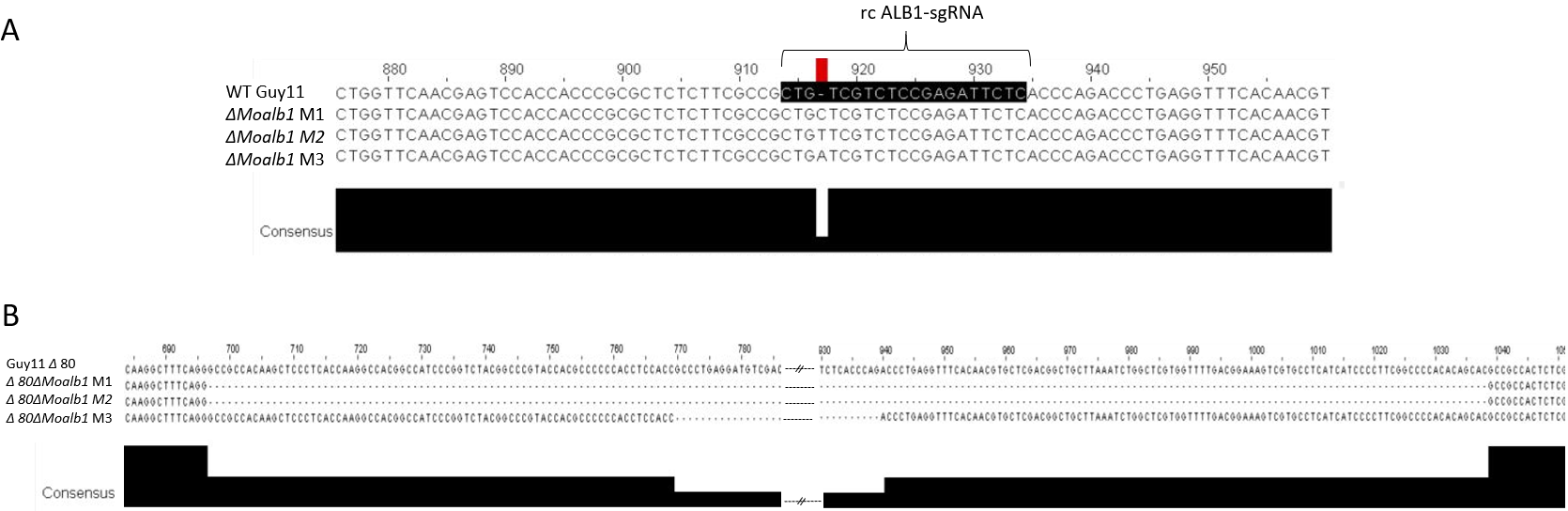

Supplement: S10 Fig — (A) Alignment of a M. oryzae ALB1 genomic sequence section (WT strain Guy11) and three edited mutants showing a white phenotype. A DNA fragment around the MoALB1-sgRNA binding site (shown as reverse complement: rc ALB1-sgRNA) was amplified with primers SeqAlb1 F/ SeqAlb1 R, and the PCR product sequenced. (B) Alignment of a MoALB1 genomic sequence (strain Guy11ku80) section with the sequences of three edited mutants showing a white phenotype. Amplification and sequencing were done as described above. Alignments were done with Jalview 2.10.4. (TIF) [file ppat.1008326.s010.tif]

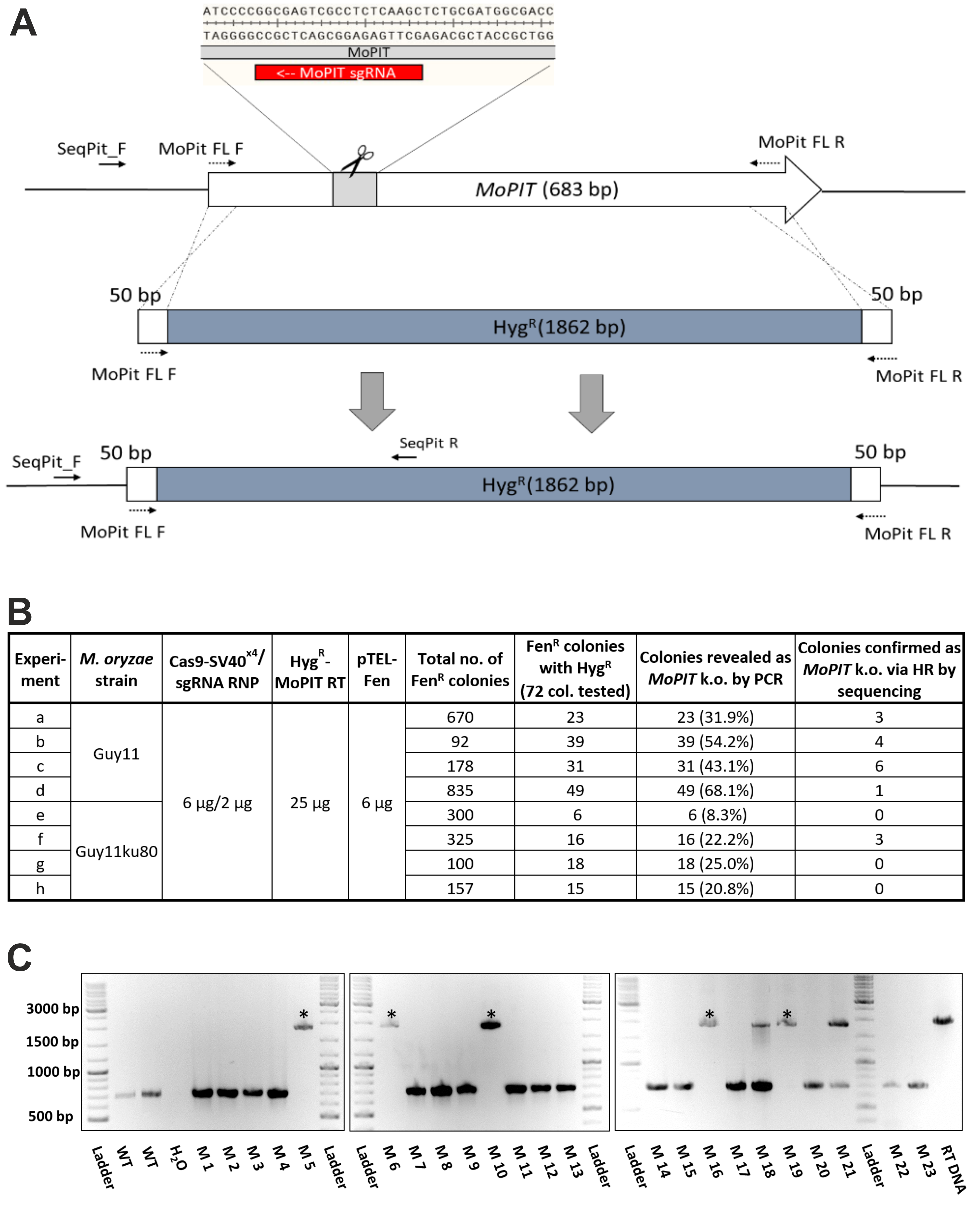

Supplement: S11 Fig — (A) Schematic illustration of MoPIT locus with sgRNA and binding sites for primers used in this study. The upper cartoon delineates the genomic region of MoPIT. The middle cartoon shows the repair template (RT) containing HygR and 50 bp flanks on each site for homologous recombination. The cartoon at the bottom displays the mutated MoPIT region after gene replacement. (B) Results for co-transformations of strains Guy11 and Guy11ku80 with pTEL-Fen, Cas9-sgRNA RNP and HygR-MoPIT repair template. Four independent experiments were performed with isolate Guy11ku80 (a-d) and Guy11 (e-f). After transformation, protoplasts were firstly selected for FenR, and 72 of the mutants were additionally tested for HygR. This revealed 8–25% for Guy11ku80 and 32–68% for Guy11 mutants being resistant to both antibiotics. PCR analyses were applied to test mutants, co-resistant to FenR and HygR, for the presence of the repair template at the MoPIT locus (see part C). From them 18% for Guy11ku80, however only in one out of four experiments, and 4–25% for Guy11, showed integration of the HygR-MoPIT repair template. To discriminate whether gene replacement was due to HR or NHEJ sequencing of PCR products, amplified with primers SeqPit5’_F/ SeqPit5’_R, and primers SeqPit3’F/ SeqPit3’R, was performed. Thus, all three Guy11ku80 mutants showed the predicted gene replacement by HR while the frequency for Guy11 varied between 50–80%. (C) PCR-based verification of pTEL-mediated gene replacement was done for all mutants co-selected for FenR and HygR (exemplary pictures are shown for mutants from experiment a, mutants M1-M23). PCR amplification was done using primers MoPit FL F and MoPit FL R. Thus, amplification of the MoPIT WT gene yielded a PCR-product size of 683 bp while the band size corresponding to successful gene replacement was 1835 bp. The presence of two PCR products, at 683 bp and 1835 bp (M18, M21), indicated an unspecific integration of the complete HygR construct into the genome witho [file ppat.1008326.s011.tif]

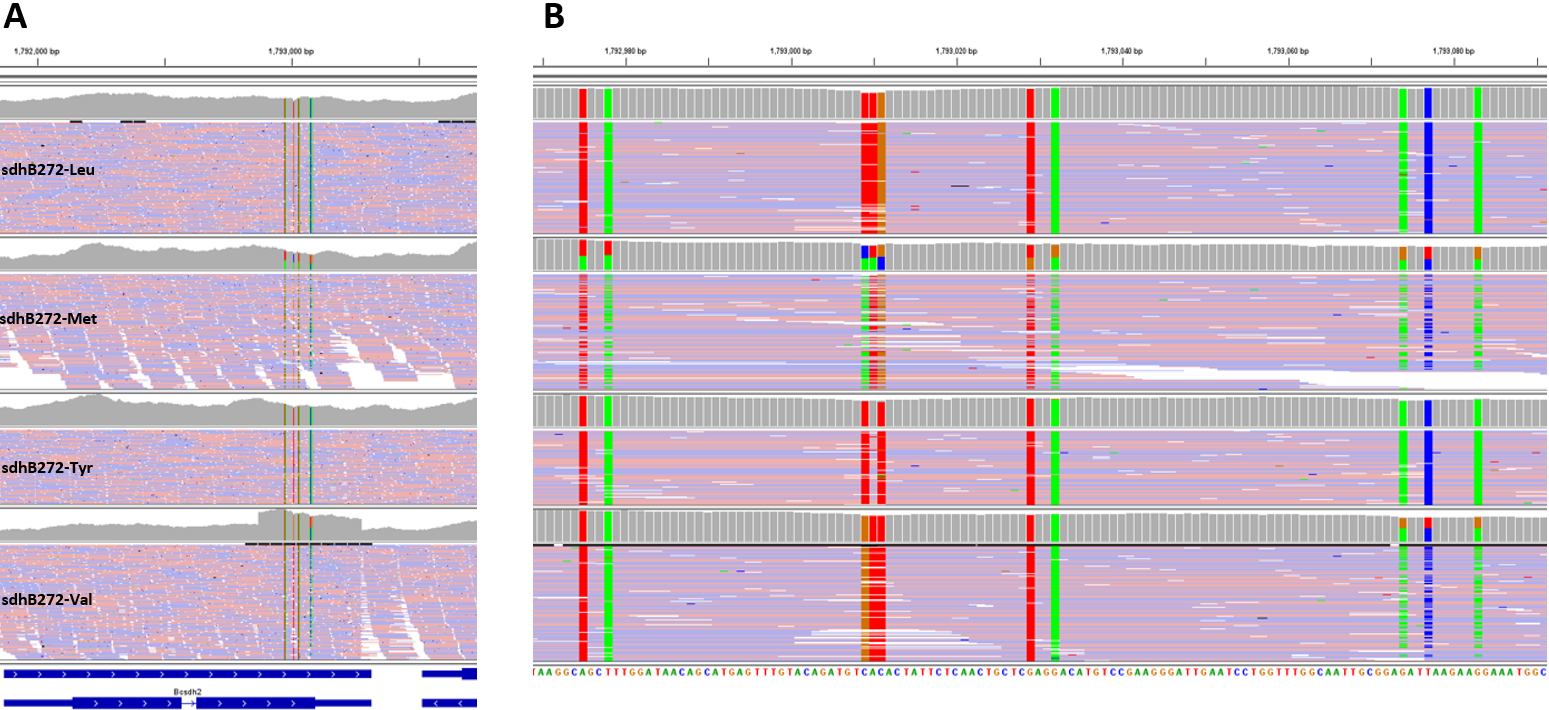

Supplement: S12 Fig — (A) Read coverage in the region surrounding sdhB. Note duplicated coverage in the region corresponding to the 500 bp sdhB repair template in sdhB272-Val. (B) Mutations in the region surrounding codon 272. Note mixed edited/ WT reads indicating heterokaryosis in strain sdhB272-Met, and mixed edited/ WT reads restricted to three positions on the right presumably due to homologous and in addition ectopic integration of the repair template in strain sdhB272-Val. (TIF) [file ppat.1008326.s012.tif]

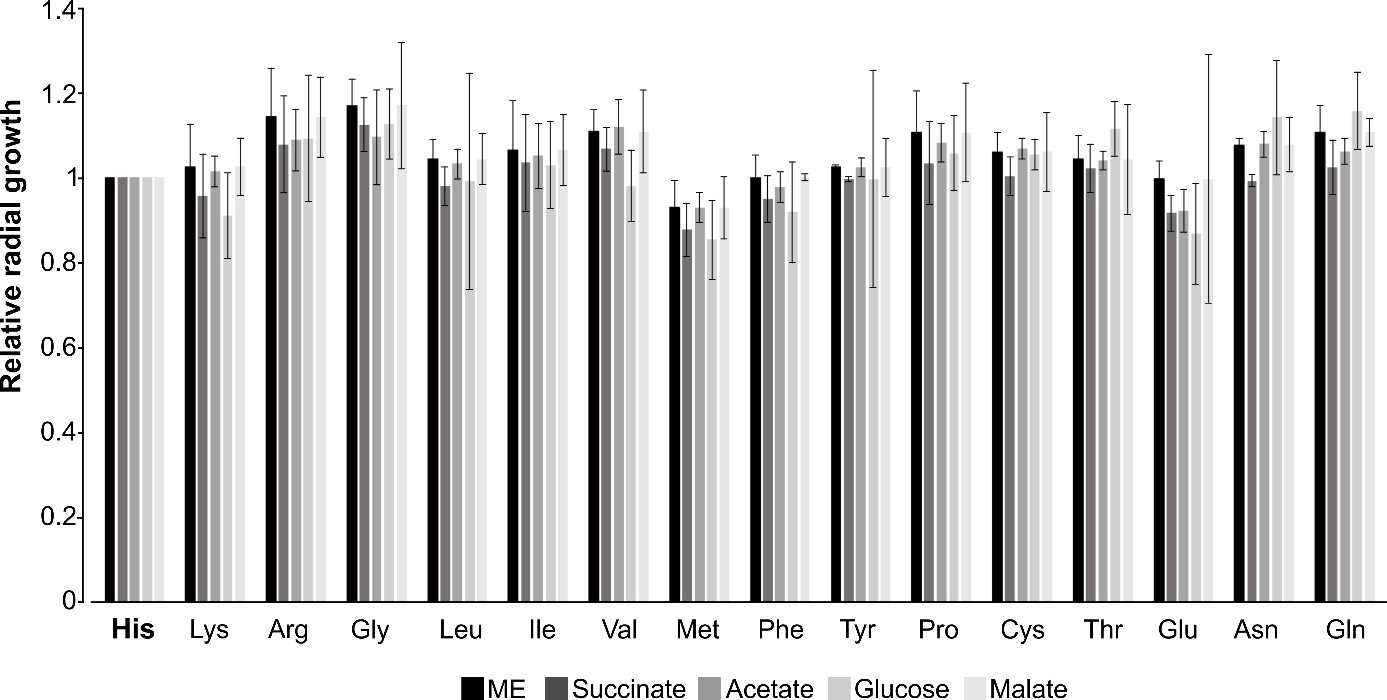

Supplement: S13 Fig — Statistical analyses were performed by analysis of variance (ANOVA) followed by Dunnett’s multiple comparisons (control: His). No significant differences between the growth rates of the WT strain (His) and any of the mutants were observed by one-way ANOVA followed by Dunnett’s multiple comparisons (control: His) post hoc test. (TIF) [file ppat.1008326.s013.tif]
