# Supplementary material for: CRISPR/Cas with ribonucleoprotein complexes and transiently selected telomere vectors allows highly efficient marker-free and multiple genome editing in Botrytis cinerea
Source: PLoS Pathog. 2020 Aug 17;16(8):e1008326. doi: 10.1371/journal.ppat.1008326 (PMC7451986; doi:10.1371/journal.ppat.1008326)
Supplement: S1 Text — (DOCX) [file ppat.1008326.s020.docx]

**Supplementary file 1: Plasmid sequences**

The sequences of the following plasmids are shown below:

1. pFB2N
2. pTEL-Fen
3. pUC-BcCas-SV40x4_nat_niaD
4. pTEL-BcCas9GFP-NLS-SV40x4
5. pTEL-BcCas9GFP-NLS-Stux2
6. pET24a_Cas9_SV40x4-NLS-His
7. pET24a_Cas9_BcStux2-NLS-His

**1. pFB2N**

LOCUS Exported 10341 bp ds-DNA circular SYN 09-DEC-2019

DEFINITION synthetic circular DNA.

ACCESSION .

VERSION .

KEYWORDS pFB2N

SOURCE synthetic DNA construct

ORGANISM synthetic DNA construct

REFERENCE 1 (bases 1 to 10341)

AUTHORS Sabine Fillinger

TITLE -

JOURNAL -

COMMENT Assemble of:

Fragment : pFAC1 : NheI at 5409 to BglII at 15628.

Assembled on 06/02/06

FEATURES Location/Qualifiers

source 1..10341

/organism="synthetic DNA construct"

/mol_type="other DNA"

source 329..430

/organism="synthetic DNA construct"

/mol_type="other DNA"

misc_feature 93..689

/label=HTEL

/note="Human telomeric region, compare Barreau et al 1998"

gap 328..431

/estimated_length="104"

CDS complement(1035..1850)

/label=KanR

/note="keep under constant selection for stabilisation of

telomere regions"

misc_feature complement(2020..2512)

/label=HTEL

/note="Human telomeric region, compare Barreau et al 1998"

misc_feature 3041..3241

/label=cos

terminator 4653..4903

/label=TtrpC

/note="terminator from A. nidulans"

CDS complement(4904..5929)

/codon_start=1

/gene="aph(4)-Ia"

/product="aminoglycoside phosphotransferase from E. coli"

/label=HygR

/note="confers resistance to hygromycin"

/translation="MKKPELTATSVEKFLIEKFDSVSDLMQLSEGEESRAFSFDVGGRG

YVLRVNSCADGFYKDRYVYRHFASAALPIPEVLDIGEFSESLTYCISRRAQGVTLQDLP

ETELPAVLQPVAEAMDAIAAADLSQTSGFGPFGPQGIGQYTTWRDFICAIADPHVYHWQ

TVMDDTVSASVAQALDELMLWAEDCPEVRHLVHADFGSNNVLTDNGRITAVIDWSEAMF

GDSQYEVANIFFWRPWLACMEQQTRYFERRHPELAGSPRLRAYMLRIGLDQLYQSLVDG

NFDDAAWAQGRCDAIVRSGAGTVGRTQIARRSAAVWTDGCVEVLADSGNRRPSTRPRAK

E"

promoter 5930..6699

/label=Pcpc1

/note="promoter from N. crassa"

CDS 7873..8064

/codon_start=1

/gene="rop"

/product="Rop protein, which maintains plasmids at low copy

number"

/label=rop

/translation="MTKQEKTALNMARFIRSQTLTLLEKLNELDADEQADICESLHDHA

DELYRSCLARFGDDGENL"

misc_feature 8166..8306

/label=bom

/note="basis of mobility region from pBR322"

rep_origin 8492..9080

/label=ori

/note="high-copy-number ColE1/pMB1/pBR322/pUC origin of

replication"

CDS complement(9251..10111)

/codon_start=1

/gene="bla"

/product="beta-lactamase"

/label=AmpR

/note="confers resistance to ampicillin, carbenicillin, and

related antibiotics"

ORIGIN

1 gatctaggcc tgcaggatgc tagcttcaga cgtgtctagg gataacaggg taattcgaac

61 ccccgcgccg cctttgcgag ggtggagttg ccttagggtt agggttaggg ttagggttag

121 ggttagggtt agggttaggg ttagggttag ggttagggtt tagggttagg gttagggtta

181 gggttagggt tagggttagg gtcagggtca ggggtagggt caggggtagg gtcaggggta

241 ggggtagggg tagggtcagg gttagggtta gggttagggt tagggttagg gttagggtta

301 gggtcagggt tagggttagg gttagggnnn nnnnnnnnnn nnnnnnnnnn nnnnnnnnnn

361 nnnnnnnnnn nnnnnnnnnn nnnnnnnnnn nnnnnnnnnn nnnnnnnnnn nnnnnnnnnn

421 nnnnnnnnnn naggggtagg ggtaggggta gggttagggt tagggttagg gttagggtta

481 gggttagggt tagggtcagg gtcagggtca ggggtagggg taggggtagg ggtaggggta

541 gggttagggg tagggttagg ggtaggggta ggggtagggt tagggttagg gtttagggtt

601 agggttaggg ttagggttag ggttaggggt tagggttagg gttagggtta gaaggttagg

661 gttagggtta gggttaaggg ttaagggtta ggtgtggggt gtggcgaatt cctcgacctg

721 caagcggccg cttgcagggg ggggggggcg ctgaggtctg cctcgtgaag aaggtgttgc

781 tgactcatac caggcctgaa tcgccccatc atccagccag aaagtgaggg agccacggtt

841 gatgagagct ttgttgtagg tggaccagtt ggtgattttg aacttttgct ttgccacgga

901 acggtctgcg ttgtcgggaa gatgcgtgat ctgatccttc aactcagcaa aagttcgatt

961 tattcaacaa agccgccgtc ccgtcaagtc agcgtaatgc tctgccagtg ttacaaccaa

1021 ttaaccaatt ctgattagaa aaactcatcg agcatcaaat gaaactgcaa tttattcata

1081 tcaggattat caataccata tttttgaaaa agccgtttct gtaatgaagg agaaaactca

1141 ccgaggcagt tccataggat ggcaagatcc tggtatcggt ctgcgattcc gactcgtcca

1201 acatcaatac aacctattaa tttcccctcg tcaaaaataa ggttatcaag tgagaaatca

1261 ccatgagtga cgactgaatc cggtgagaat ggcaaaagct tatgcatttc tttccagact

1321 tgttcaacag gccagccatt acgctcgtca tcaaaatcac tcgcatcaac caaaccgtta

1381 ttcattcgtg attgcgcctg agcgagacga aatacgcgat cgctgttaaa aggacaatta

1441 caaacaggaa tcgaatgcaa ccggcgcagg aacactgcca gcgcatcaac aatattttca

1501 cctgaatcag gatattcttc taatacctgg aatgctgttt tcccggggat cgcagtggtg

1561 agtaaccatg catcatcagg agtacggata aaatgcttga tggtcggaag aggcataaat

1621 tccgtcagcc agtttagtct gaccatctca tctgtaacat cattggcaac gctacctttg

1681 ccatgtttca gaaacaactc tggcgcatcg ggcttcccat acaatcgata gattgtcgca

1741 cctgattgcc cgacattatc gcgagcccat ttatacccat ataaatcagc atccatgttg

1801 gaatttaatc gcggcctcga gcaagacgtt tcccgttgaa tatggctcat aacacccctt

1861 gtattactgt ttatgtaagc agacagtttt attgttcatg atgatatatt tttatcttgt

1921 gcaatgtaac atcagagatt ttgagacaca acgtggcttt cccccccccc cctgcaagcg

1981 gccgcttgca ggtcgaggaa ttcgccacac cccacaccta acccttaacc cttaacccta

2041 accctaaccc taaccctaac cctaacccta accctaaccc ctaaccctaa ccctaaccct

2101 aaccctaacc ctaaacccta accctaaccc tacccctacc cctaccccta accctacccc

2161 taaccctacc cctaccccta cccctacccc tacccctgac cctgaccctg accctaaccc

2221 taaccctaac cctaacccta accctaaccc taaccctacc cctaccccta cccctnnccc

2281 taaccctaac cctaaccctg accctaaccc taaccctaac cctaacccta accctaaccc

2341 taaccctgac cctaccccta cccctacccc tgaccctacc cctgacccta cccctgaccc

2401 tgaccctaac cctaacccta accctaaccc taaccctaac cctaaaccct aaccctaacc

2461 ctaaccctaa ccctaaccct aaccctaacc ctaaccctaa ccctaaccct aaggcaactc

2521 caccctcgca aaggcggcgc gggggttcga attaccctgt tatccctaga tacgtctgct

2581 ttttgttgac ttccattgtt cattccacgg acaaaaacag agaaaggaaa cgacagaggc

2641 caaaaagctc gctttcagca cctgtcgttt cctttctttt cagagggtat tttaaataaa

2701 aacattaagt tatgacgaag aagaacggaa acgccttaaa ccggaaaatt ttcataaata

2761 gcgaaaaccc gcgaggtcgc cgccccgtaa caaggcggat cgccggaaag gacccgcaaa

2821 tgataataat tatcaattgc atactatcga cggcactgct gccagataac accaccgggg

2881 aaacattcca tcatgatggc cgtgcggaca taggaagcca gttcatccat cgctttcttg

2941 tctgctgcca tttgctttgt gacatccagc gccgcacatt cagcagcgtt tttcagcgcg

3001 ttttcgatca acgtttcaat gttggtatca acaccaggtt taactttgaa cttatcggca

3061 ctgacggtta ccttgttctg cgctggctca tcacgcagga taccaaggct gatgttgtag

3121 atattggtca ccggctgagg gttttcgatt gccgctgcgt ggatagcacc atttgcgatc

3181 aggcgtcctt gatgaatgac actccattgc gaataagttc gaaggagacg gtgtcacgaa

3241 tgcgctggtc cagctcggtc gattgccttt tgtgcagcag aggtatcaat ctcaacgcca

3301 aggctcatcg aagcgcaata ttgctgctca ccaaaacgcg tattgaccag gtgttcaacg

3361 gcaaatttct gcccttctga tgtcagaaag gcaaagtgat tttctttctg gtattcagtt

3421 gctgtgtgtc ggtttcagca aaaccaagct cgcgcaattc ggctgtgcag atttagaagg

3481 cagatcacca gacagcaacg gccaacggaa aacagcgcat acagaacatc cgtcgccgcg

3541 ccgacaacgt gataattttt atgacccatg atttatttcc ttttagacgt gagcctgtcg

3601 cacagcaaag ccgccgaaag ttcctcgacc gatgcctcga ccgatgccct tgagagcctt

3661 caacccagtc agctccttcc ggtgggcgcg gggcatgact atcgtcgccg cacttatgac

3721 tgtcttcttt atcatgcaac tcgtaggaca ggtgccggca gcgctctggg tcattttcgg

3781 cgaggaccgc tttcgctgga gcgcgacgat gatcggcctg tcgcttgcgg tattcggaat

3841 cttgcacgcc ctcgctcaag ccttcgtcac tggtcccgcc accaaacgtt tcggcgagaa

3901 gcaggccatt atcgccggca tggcggccga cgcgctgggc tacgtcttgc tggcgttcga

3961 gcttgcatgc aaggagatgg cgcccaacag tcccccggcc acggggcctg ccaccatacc

4021 cacgccgaaa caagcgctca tgagcccgaa gtggcgagcc cgatcttccc catcggtgat

4081 gtcggcgata taggcgccag caaccgcacc tgtggcgccg gtgatgccgg ccacgatgcg

4141 tccggcgtag aggatcctct agctagaaag aaggattacc tctaaacaag tgtacctgtg

4201 cattctgggt aaacgactca taggagagtt gtaaaaaagt ttcggccggc gtattgggtg

4261 ttacggagca ttcactaggc aaccatggtt actattgtat acccatctta gtaggaatga

4321 ttttcgaggt ttatacctac gatgaatgtg tgtcctgtag gcttgagagt tcaaggaaga

4381 aacagtgcaa ttatctttgc gaacccaggg gctggtgacg gaattttcat agtcaagcta

4441 tcagagttaa gaagaggagc atgtcaaagt acaattagag acaaatatat agtcgcgtgg

4501 agccaagagc ggattcctca gtctcgtagg tctcttgacg accgttgatc tgcttgatct

4561 cgtctcccga aaatgaaaat agactctgct aagctattct tctgcttcgc cggagcctga

4621 agggcgtact agggttgcga ggtccaatgc attaatgcat tgcagatgag ctgtatctgg

4681 aagaggtaaa cccgaaacgc gttttattct tgttgacatg gagctattaa atcactagaa

4741 ggcactcttt gctgcttgga caaatgaacg tatcttatcg agatcctgaa caccatttgt

4801 ctcaactccg gagctgacat cgacaccaac gatcttatat ccagattcgt caagctgttt

4861 gatgatttca gtaacgttaa gtggatcccg gtcggcatct actctattcc tttgccctcg

4921 gacgagtgct ggggcgtcgg tttccactat cggcgagtac ttctacacag ccatcggtcc

4981 agacggccgc gcttctgcgg gcgatttgtg tacgcccgac agtcccggct ccggatcgga

5041 cgattgcgtc gcatcgaccc tgcgcccaag ctgcatcatc gaaattgccg tcaaccaagc

5101 tctgatagag ttggtcaaga ccaatgcgga gcatatacgc ccggaggcgc ggcgatcctg

5161 caagctccgg atgcctccgc tcgaagtagc gcgtctgctg ctccatacaa gccaaccacg

5221 gcctccagaa gaagatgttg gcgacctcgt attgggaatc cccgaacatc gcctcgctcc

5281 agtcaatgac cgctgttatg cggccattgt ccgtcaggac attgttggag ccgaaatccg

5341 cgtgcacgag gtgccggact tcggggcagt cctcggccca aagcatcagc tcatcgagag

5401 cctgcgcgac ggacgcactg acggtgtcgt ccatcacagt ttgccagtga tacacatggg

5461 gatcagcaat cgcgcatatg aaatcacgcc atgtagtgta ttgaccgatt ccttgcggtc

5521 cgaatgggcc gaacccgctc gtctggctaa gatcggccgc agcgatcgca tccatggcct

5581 ccgcgaccgg ctgcagaaca gcgggcagtt cggtttcagg caggtcttgc aacgtgacac

5641 cctgtgcacg gcgggagatg caataggtca ggctctcgct gaattcccca atgtcaagca

5701 cttccggaat cgggagcgcg gccgatgcaa agtgccgata aacataacga tctttgtaga

5761 aaccatcggc gcagctattt acccgcagga catatccacg ccctcctaca tcgaagctga

5821 aagcacgaga ttcttcgccc tccgagagct gcatcaggtc ggagacgctg tcgaactttt

5881 cgatcagaaa cttctcgaca gacgtcgcgg tgagttcagg ctttttcata tgggtacctg

5941 agaacatctt gttgccctgc tttccgtgcg aaatactacc ggtacttttg ggaaacaagg

6001 gaacaggagg gcgctgctgt gcgcggttct gagtgttcag gattgaagct gaagaaggtg

6061 ctgaggaagc gtagaactgt tgcggacgcg agttctgaga agagctgtac cgattggtga

6121 aagccgaaga agtgagttgg tgccctgttg cctggataat gtttgcaact cgctggttct

6181 gcagagacgg agacaaatgc tggctacgat gttgctgatt caggttgata cctcggtcga

6241 gatactgttt tggtttgata gggtggattt ggttgcagag aagaagaaag gaaggtcaaa

6301 gagggaaaac tgggcggagg gaaggatttt gtatcaggca gcaaactgcc actgcagtgg

6361 ccctggcagt gccgggcgag gcacccacgc acggccgcgc aaccggttgg tccttgccca

6421 ccacgaaacc cttctgaaag gtcagatgga agtgtgcgac agtgcgcgtc cccaagccaa

6481 tgcaggcgcc atggatccac tccccacccg caagatttca ctgtgcgttc ttattggttg

6541 ccgcaaggcc agccaaaggg ggaagtatga gtcacagcac cgatacaaga aaattgcaga

6601 actaacatat ggatgcgcgc gctattctgt agagctctgg gcaaagcacc aatcctgcgg

6661 gtcggtacac acactagcac tgccccacct gaggcagtca gccccgctga ccgaattgcc

6721 aagagccaat ggagacggaa agccaacgct gatggagcac catctgaatg gacctcgctc

6781 gcttgcctgg aagggacaag ggacaccgga gacagggcct ccaaccaagg gcgcgggaaa

6841 gacgatcccc aaagtcgcaa cggcccagaa agaacgcatc catcacattc ggatgggatg

6901 gcttgaagcc agcttgcagc aacttcaaag ctcgacgcga ggctggatgg ccttccccat

6961 tatgattctt ctcgcttccg gcggcatcgg gatgcccgcg ttgcaggcca tgctgtccag

7021 gcaggtagat gacgaccatc agggacagct tcaaggatcg ctcgcggctc ttaccagcct

7081 aacttcgatc attggaccgc tgatcgtcac ggcgatttat gccgcctcgg cgagcacatg

7141 gaacgggttg gcatggattg taggcgccgc cctatacctt gtctgcctcc ccgcgttgcg

7201 tcgcggtgca tggagccggg ccacctcgac ctgaatggaa gccggcggca cctcgctaac

7261 ggattcacca ctccaagaat tggagccaat caattcttgc ggagaactgt gaatgcgcaa

7321 accaaccctt ggcagaacat atccatcgcg tccgccatct ccagcagccg cacgcggcgc

7381 atctcgggca gcgttgggtc ctggccacgg gtgcgcatga tcgtgctcct gtcgttgagg

7441 acccggctag gctggcgggg ttgccttact ggttagcaga atgaatcacc gatacgcgag

7501 cgaacgtgaa gcgactgctg ctgcaaaacg tctgcgacct gagcaacaac atgaatggtc

7561 ttcggtttcc gtgtttcgta aagtctggaa acgcggaagt cagcgccctg caccattatg

7621 ttccggatct gcatcgcagg atgctgctgg ctaccctgtg gaacacctac atctgtatta

7681 acgaagcgct ggcattgacc ctgagtgatt tttctctggt cccgccgcat ccataccgcc

7741 agttgtttac cctcacaacg ttccagtaac cgggcatgtt catcatcagt aacccgtatc

7801 gtgagcatcc tctctcgttt catcggtatc attaccccca tgaacagaaa tcccccttac

7861 acggaggcat cagtgaccaa acaggaaaaa accgccctta acatggcccg ctttatcaga

7921 agccagacat taacgcttct ggagaaactc aacgagctgg acgcggatga acaggcagac

7981 atctgtgaat cgcttcacga ccacgctgat gagctttacc gcagctgcct cgcgcgtttc

8041 ggtgatgacg gtgaaaacct ctgacacatg cagctcccgg agacggtcac agcttgtctg

8101 taagcggatg ccgggagcag acaagcccgt cagggcgcgt cagcgggtgt tggcgggtgt

8161 cggggcgcag ccatgaccca gtcacgtagc gatagcggag tgtatactgg cttaactatg

8221 cggcatcaga gcagattgta ctgagagtgc accatatgcg gtgtgaaata ccgcacagat

8281 gcgtaaggag aaaataccgc atcaggcgct cttccgcttc ctcgctcact gactcgctgc

8341 gctcggtcgt tcggctgcgg cgagcggtat cagctcactc aaaggcggta atacggttat

8401 ccacagaatc aggggataac gcaggaaaga acatgtgagc aaaaggccag caaaaggcca

8461 ggaaccgtaa aaaggccgcg ttgctggcgt ttttccatag gctccgcccc cctgacgagc

8521 atcacaaaaa tcgacgctca agtcagaggt ggcgaaaccc gacaggacta taaagatacc

8581 aggcgtttcc ccctggaagc tccctcgtgc gctctcctgt tccgaccctg ccgcttaccg

8641 gatacctgtc cgcctttctc ccttcgggaa gcgtggcgct ttctcatagc tcacgctgta

8701 ggtatctcag ttcggtgtag gtcgttcgct ccaagctggg ctgtgtgcac gaaccccccg

8761 ttcagcccga ccgctgcgcc ttatccggta actatcgtct tgagtccaac ccggtaagac

8821 acgacttatc gccactggca gcagccactg gtaacaggat tagcagagcg aggtatgtag

8881 gcggtgctac agagttcttg aagtggtggc ctaactacgg ctacactaga aggacagtat

8941 ttggtatctg cgctctgctg aagccagtta ccttcggaaa aagagttggt agctcttgat

9001 ccggcaaaca aaccaccgct ggtagcggtg gtttttttgt ttgcaagcag cagattacgc

9061 gcagaaaaaa aggatctcaa gaagatcctt tgatcttttc tacggggtct gacgctcagt

9121 ggaacgaaaa ctcacgttaa gggattttgg tcatgagatt atcaaaaagg atcttcacct

9181 agatcctttt aaattaaaaa tgaagtttta aatcaatcta aagtatatat gagtaaactt

9241 ggtctgacag ttaccaatgc ttaatcagtg aggcacctat ctcagcgatc tgtctatttc

9301 gttcatccat agttgcctga ctccccgtcg tgtagataac tacgatacgg gagggcttac

9361 catctggccc cagtgctgca atgataccgc gagacccacg ctcaccggct ccagatttat

9421 cagcaataaa ccagccagcc ggaagggccg agcgcagaag tggtcctgca actttatccg

9481 cctccatcca gtctattaat tgttgccggg aagctagagt aagtagttcg ccagttaata

9541 gtttgcgcaa cgttgttgcc attgctgcag gcatcgtggt gtcacgctcg tcgtttggta

9601 tggcttcatt cagctccggt tcccaacgat caaggcgagt tacatgatcc cccatgttgt

9661 gcaaaaaagc ggttagctcc ttcggtcctc cgatcgttgt cagaagtaag ttggccgcag

9721 tgttatcact catggttatg gcagcactgc ataattctct tactgtcatg ccatccgtaa

9781 gatgcttttc tgtgactggt gagtactcaa ccaagtcatt ctgagaatag tgtatgcggc

9841 gaccgagttg ctcttgcccg gcgtcaacac gggataatac cgcgccacat agcagaactt

9901 taaaagtgct catcattgga aaacgttctt cggggcgaaa actctcaagg atcttaccgc

9961 tgttgagatc cagttcgatg taacccactc gtgcacccaa ctgatcttca gcatctttta

10021 ctttcaccag cgtttctggg tgagcaaaaa caggaaggca aaatgccgca aaaaagggaa

10081 taagggcgac acggaaatgt tgaatactca tactcttcct ttttcaatat tattgaagca

10141 tttatcaggg ttattgtctc atgagcggat acatatttga atgtatttag aaaaataaac

10201 aaataggggt tccgcgcaca tttccccgaa aagtgccacc tgacgtctaa gaaaccatta

10261 ttatcatgac attaacctat aaaaataggc gtatcacgag gccctttcgt cttcaagaat

10321 tcgcggcccc gcatgggccc a

//

**2. pTEL-Fen**

LOCUS Exported 6923 bp ds-DNA circular SYN 09-DEC-2019

DEFINITION synthetic circular DNA.

ACCESSION .

VERSION .

KEYWORDS .

SOURCE synthetic DNA construct

ORGANISM synthetic DNA construct

REFERENCE 1 (bases 1 to 6923)

AUTHORS Thomas Leisen

TITLE -

JOURNAL -

FEATURES Location/Qualifiers

source 1..6923

/organism="synthetic DNA construct"

/mol_type="other DNA"

misc_feature 93..689

/label=HTEL

/note="Human telomeric region, compare Barreau et al 1998"

gap 328..431

/estimated_length="104"

CDS complement(1035..1850)

/label=KanR

/note="keep under constant selection for stabilisation of

telomere regions"

misc_feature complement(2020..2512)

/label=HTEL

/note="Human telomeric region, compare Barreau et al 1998"

terminator 2842..3000

/label=TniaD

/note="terminator from B. cinerea"

CDS complement(join(3001..3813,3863..4390,4446..4532))

/codon_start=1

/label=fferg27

/note="fferg27 (FFUJ_04356)"

/translation="MGSQLSPATAPWEGVSGQEQLFVLITGANSGIGLSIGERLIDEFL

ATRSLRSHLILIPTTRSKSKSLQTIQTLRGYANKAAQSSTALRSRAGSSYRWEDTIARI

HVLSLQLDLCDLRGVYSFANALLRGPVSNPEGLQGEYLRNVRIPRLDTVVFNAAYGGWS

GVNYPKAVWTILTQGLVQSVTWPNFKMALPTALLNEKRNYNYPKEPLLGEVFTACVFGH

YILAHELLPLLSRRSETETPGRLVWSSSLEAVDSVLDMSDFQCFNGKGPYESAKRVTDI

LSLTATLPAAMPSSSRFFTPDDPSEARDKPIRPRMYLTHPGIVASTLFPVPWFLMWAYE

LALLISRWIGSPWHNTDSYTGAKSPVWIALQEQSALDELGAERIKWGSSSNRHMQVEVK

KTEVEGWGWEGKVEDAAALEADTAVGVFRKTIGRKRGAKDVTKEDIVRFEELGAECWER

MENMRHEWETILRVKKA"

promoter 4533..4876

/label=PtrpC

/note="PtrpC

promoter from A. nidulans"

rep_origin 5074..5662

/label=ori

/note="high-copy-number ColE1/pMB1/pBR322/pUC origin of

replication"

CDS complement(5833..6693)

/gene="bla"

/product="beta-lactamase"

/label=AmpR

/note="confers resistance to ampicillin, carbenicillin, and

related antibiotics"

ORIGIN

1 gatctaggcc tgcaggatgc tagcttcaga cgtgtctagg gataacaggg taattcgaac

61 ccccgcgccg cctttgcgag ggtggagttg ccttagggtt agggttaggg ttagggttag

121 ggttagggtt agggttaggg ttagggttag ggttagggtt tagggttagg gttagggtta

181 gggttagggt tagggttagg gtcagggtca ggggtagggt caggggtagg gtcaggggta

241 ggggtagggg tagggtcagg gttagggtta gggttagggt tagggttagg gttagggtta

301 gggtcagggt tagggttagg gttagggnnn nnnnnnnnnn nnnnnnnnnn nnnnnnnnnn

361 nnnnnnnnnn nnnnnnnnnn nnnnnnnnnn nnnnnnnnnn nnnnnnnnnn nnnnnnnnnn

421 nnnnnnnnnn naggggtagg ggtaggggta gggttagggt tagggttagg gttagggtta

481 gggttagggt tagggtcagg gtcagggtca ggggtagggg taggggtagg ggtaggggta

541 gggttagggg tagggttagg ggtaggggta ggggtagggt tagggttagg gtttagggtt

601 agggttaggg ttagggttag ggttaggggt tagggttagg gttagggtta gaaggttagg

661 gttagggtta gggttaaggg ttaagggtta ggtgtggggt gtggcgaatt cctcgacctg

721 caagcggccg cttgcagggg ggggggggcg ctgaggtctg cctcgtgaag aaggtgttgc

781 tgactcatac caggcctgaa tcgccccatc atccagccag aaagtgaggg agccacggtt

841 gatgagagct ttgttgtagg tggaccagtt ggtgattttg aacttttgct ttgccacgga

901 acggtctgcg ttgtcgggaa gatgcgtgat ctgatccttc aactcagcaa aagttcgatt

961 tattcaacaa agccgccgtc ccgtcaagtc agcgtaatgc tctgccagtg ttacaaccaa

1021 ttaaccaatt ctgattagaa aaactcatcg agcatcaaat gaaactgcaa tttattcata

1081 tcaggattat caataccata tttttgaaaa agccgtttct gtaatgaagg agaaaactca

1141 ccgaggcagt tccataggat ggcaagatcc tggtatcggt ctgcgattcc gactcgtcca

1201 acatcaatac aacctattaa tttcccctcg tcaaaaataa ggttatcaag tgagaaatca

1261 ccatgagtga cgactgaatc cggtgagaat ggcaaaagct tatgcatttc tttccagact

1321 tgttcaacag gccagccatt acgctcgtca tcaaaatcac tcgcatcaac caaaccgtta

1381 ttcattcgtg attgcgcctg agcgagacga aatacgcgat cgctgttaaa aggacaatta

1441 caaacaggaa tcgaatgcaa ccggcgcagg aacactgcca gcgcatcaac aatattttca

1501 cctgaatcag gatattcttc taatacctgg aatgctgttt tcccggggat cgcagtggtg

1561 agtaaccatg catcatcagg agtacggata aaatgcttga tggtcggaag aggcataaat

1621 tccgtcagcc agtttagtct gaccatctca tctgtaacat cattggcaac gctacctttg

1681 ccatgtttca gaaacaactc tggcgcatcg ggcttcccat acaatcgata gattgtcgca

1741 cctgattgcc cgacattatc gcgagcccat ttatacccat ataaatcagc atccatgttg

1801 gaatttaatc gcggcctcga gcaagacgtt tcccgttgaa tatggctcat aacacccctt

1861 gtattactgt ttatgtaagc agacagtttt attgttcatg atgatatatt tttatcttgt

1921 gcaatgtaac atcagagatt ttgagacaca acgtggcttt cccccccccc cctgcaagcg

1981 gccgcttgca ggtcgaggaa ttcgccacac cccacaccta acccttaacc cttaacccta

2041 accctaaccc taaccctaac cctaacccta accctaaccc ctaaccctaa ccctaaccct

2101 aaccctaacc ctaaacccta accctaaccc tacccctacc cctaccccta accctacccc

2161 taaccctacc cctaccccta cccctacccc tacccctgac cctgaccctg accctaaccc

2221 taaccctaac cctaacccta accctaaccc taaccctacc cctaccccta cccctnnccc

2281 taaccctaac cctaaccctg accctaaccc taaccctaac cctaacccta accctaaccc

2341 taaccctgac cctaccccta cccctacccc tgaccctacc cctgacccta cccctgaccc

2401 tgaccctaac cctaacccta accctaaccc taaccctaac cctaaaccct aaccctaacc

2461 ctaaccctaa ccctaaccct aaccctaacc ctaaccctaa ccctaaccct aaggcaactc

2521 caccctcgca aaggcggcgc gggggttcga attaccctgt tatccctaga tacgtctgct

2581 ttttgttgac ttccattgtt cattccacgg acaaaaacag agaaaggaaa cgacagaggc

2641 caaaaagctc gctttcagca cctgtcgttt cctttctttt cagagggtat tttaaataaa

2701 aacattaagt tatgacgaag aagaacggaa acgccttaaa ccggaaaatt ttcataaata

2761 gcgaaaaccc gcgaggtcgc cgccccgtaa caaggcggat cgccggaaag gacccgcaaa

2821 tgataataat tatcaattcc taggctcact gatacatctg gcacctactt tataataata

2881 ctttactgaa atactttata gatactatat tcaaacatcc tctctcccat tattttagac

2941 gcagttcaat ggcaaaaact acaaccatat ctaaaccacc tctcagttac ttaaaacctc

3001 ctatgccttt ttaaccctaa gaatggtttc ccactcgtgt cgcatgttct ccatcctttc

3061 ccagcattcg gcgcccagtt cctcgaatct cacgatatct tccttggtca cgtctttagc

3121 gcctctcttt cgcccgatgg tcttcctgaa aacaccaaca gcggtgtccg cttcaagcgc

3181 cgccgcatcc tcgactttgc cctcccaacc ccagccttca acttctgtct tcttaacttc

3241 gacctgcatg tgacggttcg agctgctgcc ccacttgatg cgctctgcac ctagttcatc

3301 aagcgcagat tgctcttgga gcgcaatcca aacaggggat ttggcaccgg tatagctgtc

3361 tgtgttgtgc cagggcgaac caatccagcg actgatcaga agagcaagtt catatgccca

3421 catcagaaac cagggaactg ggaatagggt actggcaaca atacccggat gggtgagata

3481 catgcgcggt cgaatgggtt tatcgcgggc ctcgctgggg tcgtcggggg tgaagaagcg

3541 gctggaagat ggcatggctg cggggagagt tgcagtgagg gagagaatgt cggtgactcg

3601 tttagcggat tcgtacgggc ctttaccatt gaagcactgg aagtcggaca tatcaagaac

3661 actgtcgacg gcctcaagac tgctggacca gacaagacgg ccgggtgttt cggtttcgga

3721 tcgccgacta agtagtggaa gaagttcatg ggccaatata taatgaccaa aaacacaggc

3781 tgtaaacacc tcgccgagta aaggttcctt gggctgcatt cagtcaatgg gtgaaatcat

3841 tgagttactg cgactgactc acatagttgt agttgcgctt ttcgttcaag agagcagtag

3901 ggagggccat cttgaagttt ggccaagtaa cagactgcac caacccctgg gtcaagatag

3961 tccatacagc tttgggataa ttgacccctg accatccacc atacgcagca ttgaatacta

4021 cagtatccag cctgggaatg cgtacatttc gtagatattc gccctgcagc ccctcagggt

4081 tgctcaccgg acctcgtaac aaagcatttg cgaaagaata caccccgcgc aagtcacaca

4141 ggtcaagctg caagctcaga acatggattc gcgcaatggt atcttcccaa cgatatgaac

4201 tcccagcccg cgaacgtagt gctgtagaag attgcgcagc cttgttggcg tagccacgaa

4261 gggtttggat cgtttgtaag gacttggact tggatcgtgt ggtagggatg aggatgaggt

4321 gggagcgcaa agagcgtgta gcaaggaatt cgtcgataag tcgttctcca atactgagac

4381 cgataccact tgagttgcat tagctgtcgc tccctatata tagcctggag cttttgaaca

4441 aacctattgg cacctgtgat cagaacaaaa agttgttcct gacccgagac tccctcccag

4501 ggggctgtcg ccggcgacaa ttgggagccc atttggatgc ttgggtagaa taggtaagtc

4561 agattgaatc tgaaataaag ggaggaaggg cgaacttaag aaggtatgac cgggtcgtcc

4621 acttaccttg cttgacaaac gcaccaagtt atcgtgcacc aagcagcaga tgataataat

4681 gtcctcgttc ctgtctgcta ataagagtca cacttcgagc gccgccgcta ctgctacaag

4741 tggggctgat ctgaccagtt gcctaaatga accatcttgt caaacgacac aaattttgtg

4801 ctcaccgcct ggacgactaa accaaaatag gcattcattg ttgacctcca ctagctccag

4861 ccaagcccaa aaaatggcgc gccatatggt accggatgaa ttgcaatggg cgtggaagac

4921 agcgaatgtg acgacttctg aagataaagc tggtgcagcc ccaccacaat ggcccttgga

4981 cttgagcatc aatccggagc tggatcctac atgcatgtga gcaaaaggcc agcaaaaggc

5041 caggaaccgt aaaaaggccg cgttgctggc gtttttccat aggctccgcc cccctgacga

5101 gcatcacaaa aatcgacgct caagtcagag gtggcgaaac ccgacaggac tataaagata

5161 ccaggcgttt ccccctggaa gctccctcgt gcgctctcct gttccgaccc tgccgcttac

5221 cggatacctg tccgcctttc tcccttcggg aagcgtggcg ctttctcata gctcacgctg

5281 taggtatctc agttcggtgt aggtcgttcg ctccaagctg ggctgtgtgc acgaaccccc

5341 cgttcagccc gaccgctgcg ccttatccgg taactatcgt cttgagtcca acccggtaag

5401 acacgactta tcgccactgg cagcagccac tggtaacagg attagcagag cgaggtatgt

5461 aggcggtgct acagagttct tgaagtggtg gcctaactac ggctacacta gaaggacagt

5521 atttggtatc tgcgctctgc tgaagccagt taccttcgga aaaagagttg gtagctcttg

5581 atccggcaaa caaaccaccg ctggtagcgg tggttttttt gtttgcaagc agcagattac

5641 gcgcagaaaa aaaggatctc aagaagatcc tttgatcttt tctacggggt ctgacgctca

5701 gtggaacgaa aactcacgtt aagggatttt ggtcatgaga ttatcaaaaa ggatcttcac

5761 ctagatcctt ttaaattaaa aatgaagttt taaatcaatc taaagtatat atgagtaaac

5821 ttggtctgac agttaccaat gcttaatcag tgaggcacct atctcagcga tctgtctatt

5881 tcgttcatcc atagttgcct gactccccgt cgtgtagata actacgatac gggagggctt

5941 accatctggc cccagtgctg caatgatacc gcgagaccca cgctcaccgg ctccagattt

6001 atcagcaata aaccagccag ccggaagggc cgagcgcaga agtggtcctg caactttatc

6061 cgcctccatc cagtctatta attgttgccg ggaagctaga gtaagtagtt cgccagttaa

6121 tagtttgcgc aacgttgttg ccattgctgc aggcatcgtg gtgtcacgct cgtcgtttgg

6181 tatggcttca ttcagctccg gttcccaacg atcaaggcga gttacatgat cccccatgtt

6241 gtgcaaaaaa gcggttagct ccttcggtcc tccgatcgtt gtcagaagta agttggccgc

6301 agtgttatca ctcatggtta tggcagcact gcataattct cttactgtca tgccatccgt

6361 aagatgcttt tctgtgactg gtgagtactc aaccaagtca ttctgagaat agtgtatgcg

6421 gcgaccgagt tgctcttgcc cggcgtcaac acgggataat accgcgccac atagcagaac

6481 tttaaaagtg ctcatcattg gaaaacgttc ttcggggcga aaactctcaa ggatcttacc

6541 gctgttgaga tccagttcga tgtaacccac tcgtgcaccc aactgatctt cagcatcttt

6601 tactttcacc agcgtttctg ggtgagcaaa aacaggaagg caaaatgccg caaaaaaggg

6661 aataagggcg acacggaaat gttgaatact catactcttc ctttttcaat attattgaag

6721 catttatcag ggttattgtc tcatgagcgg atacatattt gaatgtattt agaaaaataa

6781 acaaataggg gttccgcgca catttccccg aaaagtgcca cctgacgtct aagaaaccat

6841 tattatcatg acattaacct ataaaaatag gcgtatcacg aggccctttc gtcttcaaga

6901 attcgcggcc ccgcatgggc cca

//

**3. pUC-BcCas-SV40x4_nat_niaD**

LOCUS Exported 10786 bp ds-DNA circular SYN 26-APR-2018

DEFINITION synthetic circular DNA

ACCESSION .

VERSION .

KEYWORDS pUC57 BcCas-Sv40*4 NatSchu NiaD-Ki

SOURCE synthetic DNA construct

ORGANISM recombinant plasmid

REFERENCE 1 (bases 1 to 10786)

AUTHORS Fabian Bietz

TITLE -

JOURNAL -

COMMENT Alias: pUC57_BcCasSv40x4_NatSchu_NiaD-KI

FEATURES Location/Qualifiers

source 1..10786

/organism="recombinant plasmid"

/mol_type="other DNA"

misc_feature 402..1431

/label=NiaD 5'

/note="BcNiaD 5' Flank"

promoter 1441..2282

/label=OliC

/note="promoter from A. nidulans"

CDS 2283..6386

/codon_start=1

/product="Cas9 (Csn1) endonuclease from the Streptococcus

pyogenes Type II CRISPR/Cas system"

/label=Cas9

/note="generates RNA-guided double strand breaks in DNA"

/translation="MDKKYSIGLDIGTNSVGWAVITDEYKVPSKKFKVLGNTDRHSIKK

NLIGALLFDSGETAEATRLKRTARRRYTRRKNRICYLQEIFSNEMAKVDDSFFHRLEES

FLVEEDKKHERHPIFGNIVDEVAYHEKYPTIYHLRKKLVDSTDKADLRLIYLALAHMIK

FRGHFLIEGDLNPDNSDVDKLFIQLVQTYNQLFEENPINASGVDAKAILSARLSKSRRL

ENLIAQLPGEKKNGLFGNLIALSLGLTPNFKSNFDLAEDAKLQLSKDTYDDDLDNLLAQ

IGDQYADLFLAAKNLSDAILLSDILRVNTEITKAPLSASMIKRYDEHHQDLTLLKALVR

QQLPEKYKEIFFDQSKNGYAGYIDGGASQEEFYKFIKPILEKMDGTEELLVKLNREDLL

RKQRTFDNGSIPHQIHLGELHAILRRQEDFYPFLKDNREKIEKILTFRIPYYVGPLARG

NSRFAWMTRKSEETITPWNFEEVVDKGASAQSFIERMTNFDKNLPNEKVLPKHSLLYEY

FTVYNELTKVKYVTEGMRKPAFLSGEQKKAIVDLLFKTNRKVTVKQLKEDYFKKIECFD

SVEISGVEDRFNASLGTYHDLLKIIKDKDFLDNEENEDILEDIVLTLTLFEDREMIEER

LKTYAHLFDDKVMKQLKRRRYTGWGRLSRKLINGIRDKQSGKTILDFLKSDGFANRNFM

QLIHDDSLTFKEDIQKAQVSGQGDSLHEHIANLAGSPAIKKGILQTVKVVDELVKVMGR

HKPENIVIEMARENQTTQKGQKNSRERMKRIEEGIKELGSQILKEHPVENTQLQNEKLY

LYYLQNGRDMYVDQELDINRLSDYDVDHIVPQSFLKDDSIDNKVLTRSDKNRGKSDNVP

SEEVVKKMKNYWRQLLNAKLITQRKFDNLTKAERGGLSELDKAGFIKRQLVETRQITKH

VAQILDSRMNTKYDENDKLIREVKVITLKSKLVSDFRKDFQFYKVREINNYHHAHDAYL

NAVVGTALIKKYPKLESEFVYGDYKVYDVRKMIAKSEQEIGKATAKYFFYSNIMNFFKT

EITLANGEIRKRPLIETNGETGEIVWDKGRDFATVRKVLSMPQVNIVKKTEVQTGGFSK

ESILPKRNSDKLIARKKDWDPKKYGGFDSPTVAYSVLVVAKVEKGKSKKLKSVKELLGI

TIMERSSFEKNPIDFLEAKGYKEVKKDLIIKLPKYSLFELENGRKRMLASAGELQKGNE

LALPSKYVNFLYLASHYEKLKGSPEDNEQKQLFVEQHKHYLDEIIEQISEFSKRVILAD

ANLDKVLSAYNKHRDKPIREQAENIIHLFTLTNLGAPAAFKYFDTTIDRKRYTSTKEVL

DATLIHQSITGLYETRIDLSQLGGD"

CDS 6408..6428

/codon_start=1

/product="nuclear localization signal of SV40 large T

antigen"

/label=SV40 NLS

/translation="PKKKRKV"

CDS 6438..6458

/codon_start=1

/product="nuclear localization signal of SV40 large T

antigen"

/label=SV40 NLS

/translation="PKKKRKV"

CDS 6468..6488

/codon_start=1

/product="nuclear localization signal of SV40 large T

antigen"

/label=SV40 NLS

/translation="PKKKRKV"

CDS 6498..6518

/codon_start=1

/product="nuclear localization signal of SV40 large T

antigen"

/label=SV40 NLS

/translation="PKKKRKV"

CDS 6519..6545

/codon_start=1

/product="HA (human influenza hemagglutinin) epitope tag"

/label=HA

/translation="YPYDVPDYA"

CDS 6558..6575

/codon_start=1

/product="6xHis affinity tag"

/label=6xHis

/translation="HHHHHH"

terminator 6591..7090

/label=T gluc

/note="terminator from B. cinerea"

CDS complement(7244..7813)

/codon_start=1

/label=NatR

/translation="MATLDDTAYRYRTSVPGDAEAIEALDGSFTTDTVFRVTATGDGFT

LREVPVDPPLTKVFPDDESDDESDDGEDGDPDSRTFVAYGDDGDLAGFVVVSYSGWNRR

LTVEDIEVAPEHRGHGVGRALMGLATEFARERGAGHLWLEVTNVNAPAIHAYRRMGFTL

CGLDTALYDGTASDGEQALYMSMPCP"

promoter complement(7828..8157)

/label=PtrpC

/note="promoter from A. nidulans

misc_feature 8181..8532

/label=NiaD 3'

/note="BcNiaD 3' Flank"

protein_bind 8589..8605

/label=lac operator

/bound_moiety="lac repressor encoded by lacI"

/note="The lac repressor binds to the lac operator to

inhibit transcription in E. coli. This inhibition can be

relieved by adding lactose or

isopropyl-beta-D-thiogalactopyranoside (IPTG)."

promoter complement(8613..8643)

/label=lac promoter

/note="promoter for the E. coli lac operon"

protein_bind 8658..8679

/label=CAP binding site

/bound_moiety="E. coli catabolite activator protein"

/note="CAP binding activates transcription in the presence

of cAMP."

rep_origin complement(8967..9555)

/direction=LEFT

/label=ori

/note="high-copy-number ColE1/pMB1/pBR322/pUC origin of

replication"

CDS complement(9726..10586)

/codon_start=1

/gene="bla"

/product="beta-lactamase"

/label=AmpR

/note="confers resistance to ampicillin, carbenicillin, and

related antibiotics"

ORIGIN

1 tcgcgcgttt cggtgatgac ggtgaaaacc tctgacacat gcagctcccg gagacggtca

61 cagcttgtct gtaagcggat gccgggagca gacaagcccg tcagggcgcg tcagcgggtg

121 ttggcgggtg tcggggctgg cttaactatg cggcatcaga gcagattgta ctgagagtgc

181 accatatgcg gtgtgaaata ccgcacagat gcgtaaggag aaaataccgc atcaggcgcc

241 attcgccatt caggctgcgc aactgttggg aagggcgatc ggtgcgggcc tcttcgctat

301 tacgccagct ggcgaaaggg ggatgtgctg caaggcgatt aagttgggta acgccagggt

361 tttcccagtc acgacgttgt aaaacgacgg ccagtgaatt ccgatgtgaa agggcattcc

421 tctttcaaca atgtagcaag atccagacat cacttcctat tatatcgttg acttcaacgt

481 tacggtccaa cttgccacga tgtacttgtt cgcacgatgc gcatttctga tgcggggctc

541 tgaaccgtcc gaaacttctg ctttacttca atagtggctg tgaagatttt attcgggttg

601 ttggagactt ttgtggtatt ccaattccat tcgggaacac caccctttcg ttccaccaat

661 agctggataa acgggggctc gactatctcc actgcgagta tctcgtatac atcgtctggt

721 ttgatggccg tggcaaacag gaacaagctc gaataccgag ctttgactcg atgaaagctt

781 tggtggtaat tgtttctatg attccttcgc cccatgttgt ctgtcttgat acgatgccgc

841 gttgggacat ttgcctacat tgtgaactcc tggaagtaca ataatatgtc tgagatagcc

901 ctacggtcat aaagttttga cactattcaa aggaaggggt ttcacgacaa tagcacggac

961 aagttgttaa tttcttgctt ctcacacgcc tgcttgtgtt tctgttcttc tcaaaccctc

1021 aaagttgttg aactcggtct taagatcgcg aacaagacgt cgctcccaaa gcagactgcg

1081 ttattgtttt gatacacagt tcaatatgag ggacttttga tcatggcctg cgacatgact

1141 gttgcatcca cggagtaagg aagacatgaa tcgtggggat catcatgcga attgaaggct

1201 agcgtccggt aaagagcccg ggatatctag cttcttatct ttctcaaatc atttcatgaa

1261 acaatctaaa gaaccccgag taccaacaaa ggcattacca accgaatttt ccagtcaatt

1321 ggcacaataa tgtgaaaacc ttccttatca tcagttcatt gtatagacaa gcatatcaat

1381 tgtgtctcga gatcggaaat acttcgttcc cgtagctatt agtaactgga gcctgaggaa

1441 atgcatctgt ggagccgcat tccgattcgg gccggattgg tcagatttgc gtccgaggtg

1501 ccgtctatca ttctagcttg cggtcctggg cttgtgactg gtcgcgagct gccactaagt

1561 ggggcagtac cattttatcg gacccatcca gctatgggac ccactcgcaa atttttacat

1621 cattttcttt ttgctcagta acggccacct tttgtaaagc gtaaccagca aacaaattgc

1681 aattggcccg tagcaaggta gtcagggctt atcgtgatgg aggagaaggc tatatcagcc

1741 tcaaaaatat gttgccagct ggcggaagcc cggaaggtaa gtggattctt cgccgtggct

1801 ggagcaaccg gtggattcca gcgtctccga cttggactga gcaattcagc gtcacggatt

1861 cacgatagac agctcagacc gctccacggc tggcggcatt attggttaac ccggaaactc

1921 agtctccttg gccccgtccc gaagggaccc gacttaccag gctgggaaag ccagggatag

1981 aatacactgt acgggcttcg tacgggaggt tcggcgtagg gttgttccca agttttacac

2041 accccccaag acagctagcg cacgaaagac gcggagggtt tggtgaaaaa agggcgaaaa

2101 ttaagcggga gacgtattta ggtgctaggg ccggtttcct ccccattttt cttcggttcc

2161 ctttctctcc tggaagactt tctctctctc tcttcttctc ttcttccatc ctcagtccat

2221 cttcctttcc catcatccat ctcctcaccc ccatctcaac tccatcacat cacaatcgat

2281 ccatggacaa gaaatatagc atcgggctcg acattggtac caactcggtt ggttgggctg

2341 tgattacgga cgaatacaag gtgccatcca aaaagtttaa ggtccttgga aacaccgatc

2401 gtcactcaat caaaaagaat ctcattggag cccttctctt cgatagtggg gaaaccgccg

2461 aagctactcg attgaaacga acggctcgca gacgttatac acgacgcaag aatcgcatct

2521 gctacctcca agagattttc agcaacgaaa tggctaaagt tgatgactca ttctttcatc

2581 gactcgaaga aagtttcttg gtcgaggagg ataagaaaca cgagcgccat ccgatctttg

2641 gtaacattgt ggatgaggtt gcctatcacg aaaagtaccc aactatctat catcttcgta

2701 agaaacttgt cgatagcacg gacaaagctg atttgcgact tatctacttg gcactcgcgc

2761 acatgattaa gttccgcggc cattttctta tcgagggtga cctcaacccc gataattctg

2821 acgttgataa actcttcatc cagttggtcc aaacctacaa tcaactcttt gaggaaaacc

2881 ctattaatgc ttctggcgtg gatgccaaag ctatcctttc ggctcgcttg tctaagtcga

2941 gaagattgga gaaccttatc gcacaactcc ccggcgaaaa gaagaacggc ctcttcggta

3001 atttgattgc gttgtcactt ggtcttactc ctaacttcaa aagtaatttt gacttggcag

3061 aggatgcgaa actccagttg tctaaagata cgtatgatga cgatctcgac aacttgcttg

3121 cccaaatcgg tgaccagtac gctgatcttt tccttgccgc taagaatctc tcagatgcaa

3181 tccttctcag tgacattttg agagtcaaca ccgagattac taaagccccc ttgtcagcta

3241 gtatgatcaa aaggtatgat gagcaccatc aagatttgac cttgcttaag gccctcgtgc

3301 gtcagcaatt gcctgagaag tacaaagaaa tcttctttga ccaatccaaa aacggatacg

3361 cagggtatat tgatggcggt gcgagccaag aagagttcta caagtttatc aaaccgattt

3421 tggagaagat ggacggcact gaggaacttc tcgtcaaact caatcgcgaa gatttgcttc

3481 gtaaacaacg aacgttcgac aacggctcca taccgcacca gattcatctt ggcgaactcc

3541 acgccatcct tcgacgccag gaagatttct acccatttct caaagacaac cgtgagaaga

3601 tcgaaaagat tcttacattc cgaatcccct actatgtggg acctttggcc cgtggaaatt

3661 cccgatttgc ttggatgacc cgaaaaagcg aggaaaccat cactccgtgg aacttcgagg

3721 aagtcgtgga caaaggtgca tccgcgcaat cattcattga gagaatgacc aattttgata

3781 agaaccttcc gaatgaaaag gtccttccaa aacattcgtt gctctacgag tatttcaccg

3841 tgtataacga actcactaaa gtcaagtacg tgacggaggg aatgaggaaa ccagccttcc

3901 tctcagggga acaaaagaag gctatcgtcg atttgctttt taagaccaat cgtaaagtga

3961 ctgttaagca gttgaaggag gattatttca agaagattga atgtttcgac tccgtcgaga

4021 tcagcggcgt ggaagatcgc tttaacgctt ccctcggaac ctaccacgac cttctcaaga

4081 tcattaagga caaagatttc ctcgataacg aggaaaatga ggacatcttg gaagatattg

4141 tcctcacgtt gacacttttt gaggaccgcg aaatgatcga ggaaagactc aaaacatacg

4201 cccatttgtt cgacgataag gtgatgaaac agttgaaacg acgtagatac accggatggg

4261 gtcgccttag caggaaactc atcaacggca ttcgagataa gcaatctggt aagactatat

4321 tggatttcct taagtcggat ggcttcgcca accgcaattt tatgcagctt attcacgacg

4381 attccttgac gttcaaagag gacatccaga aagcacaagt ctcaggacaa ggggattccc

4441 ttcacgagca tatcgccaac cttgctggat caccggcgat caagaaaggg attcttcaga

4501 ccgtcaaagt tgtcgatgag cttgtcaaag tgatgggccg tcataagcca gaaaacatcg

4561 tgattgagat ggcccgagaa aatcagacca ctcaaaaggg tcagaagaac agccgcgaga

4621 gaatgaaacg tatcgaggaa ggcattaagg aacttggttc tcaaatcctc aaagagcacc

4681 ctgttgaaaa cacacaactc caaaatgaga aactctatct ctactatttg caaaatggac

4741 gcgacatgta cgtcgatcag gagttggaca ttaacaggtt gtcggactac gatgttgatc

4801 atatcgtccc gcaatccttc cttaaggacg atagcattga taacaaagtg ttgactcgct

4861 cagataagaa cagaggcaaa tccgacaatg ttccaagcga ggaagtggtt aagaagatga

4921 agaactactg gcgacaattg cttaatgcca aactcattac acaacgcaag tttgacaact

4981 tgaccaaagc cgagagagga gggttgagtg aactcgataa ggctggcttc atcaaacgtc

5041 aactcgtgga aacgcgacag atcacaaaac acgttgctca gattcttgat tccaggatga

5101 acacaaagta cgacgagaat gataagctca tccgtgaagt taaggtcatt accctcaagt

5161 ctaagttggt gtcggatttc cgcaaggact tccaatttta taaggttcga gagatcaaca

5221 attatcacca tgcacatgat gcgtacctca acgcagtcgt gggaactgcg ctcatcaaga

5281 agtatcccaa gttggagtcc gaatttgtct acggggatta taaggtttac gatgtccgca

5341 agatgattgc caagagtgag caggaaattg gcaaagccac ggctaagtat ttcttttact

5401 ccaacatcat gaatttcttt aagacggaga tcacactcgc caatggagaa attcgtaaac

5461 gacctttgat tgagactaac ggcgagactg gtgaaatcgt ttgggataag gggcgcgact

5521 tcgctaccgt gagaaaggtt ctcagcatgc cgcaagtcaa tattgtcaag aaaaccgagg

5581 tgcaaacagg cggtttctct aaggaatcga ttcttccaaa acgtaactct gacaaactca

5641 tcgctcgaaa gaaagattgg gaccccaaga agtatggagg gttcgattct cctacagtgg

5701 catactcggt tctcgttgtc gcgaaagttg agaagggaaa gtctaagaaa ctcaagtcgg

5761 tcaaagaact tctcgggatc accattatgg agcgctccag cttcgagaag aatcccatcg

5821 actttctcga agccaaaggc tataaggaag tcaagaaaga tttgatcatt aagttgccta

5881 agtactcttt gttcgagctt gaaaacggtc gaaaacgaat gctcgcatcg gcaggagagt

5941 tgcagaaagg gaatgaattg gcacttccct caaagtacgt gaacttcttg tatctcgcgt

6001 cccactacga gaaattgaag ggtagccctg aagataacga acagaagcaa ctttttgttg

6061 agcaacacaa gcattatctt gatgagatca ttgaacagat ttcagagttc agtaaacgcg

6121 tcattctcgc cgatgctaat ctcgacaaag tgttgtcggc ctacaacaaa caccgtgaca

6181 agccgattcg agagcaagct gaaaatatca ttcatctctt caccctcact aacttgggag

6241 caccagcagc gttcaagtat tttgatacga caatcgaccg taaacgatac acgtccacaa

6301 aagaggtgct tgatgcgacc cttattcatc aatccatcac tgggctctat gaaacccgta

6361 tcgacttgag tcaacttggg ggcgaccccg gatccgggtc ggccgacccc aagaagaagc

6421 gcaaagtagg tggaagtcca aagaagaaac ggaaagtagg cggctccccc aaaaagaagc

6481 gaaaagtggg aggttcccct aagaaaaagc ggaaggtata cccatacgat gttccagatt

6541 atgcttgata agtcgaccac caccaccacc accactgata agagctctag cgtatgtaga

6601 taagatgtat gattaggggt tgaggggaag gattatggct gaggaagtgg tttctgattc

6661 gtcttgtaca taagtattag catggaccct tgtggaggta tttgctcaaa gggggtgttt

6721 tagcggaaga caaaagaggg cggaattaaa tctcaatccg ttttcaactt tgaaaatctt

6781 gatccaacat tgtgattcca tgtatttgtg caaccaagtt tttcatcatt gattctgctg

6841 taatgtgaac aaactacaag taggaggaca tttgtttaaa gttttcagct cacgtggtat

6901 tgtggcctga caggagcaca aacgccgttt ttgagaaaac gaaagtttgg aattgaccat

6961 ccacaaccac atgcgtttcc tatgatacac ctcatgtggc gttaccatat gatattcggt

7021 ttcatatcta ggcaaagagg agaacatcat acgtacatct gatttgacaa ccccttcccc

7081 ccaacaagat gttggagatt tcagtaacgt taagtggatc gtatcttatc gagatcctga

7141 acaccatttg tctcaactcc ggagctgaca tcgacaccaa cgatcttata tccagattcg

7201 tcaagctgtt tgatgatttc agtaacgtta agtggataga tcttcagggg cagggcatgc

7261 tcatgtagag cgcctgctcg ccgtccgagg cggtgccgtc gtacagggcg gtgtccaggc

7321 cgcagagggt gaaccccatc cgccggtacg cgtggatcgc cggtgcgttg acgttggtga

7381 cctccagcca gaggtgcccg gcgccccgct cgcgggcgaa ctccgtcgcg agccccatca

7441 acgcgcgccc gaccccgtgc ccccggtgct ccggggcgac ctcgatgtcc tcgacggtca

7501 gccggcggtt ccagccggag tacgagacga ccacgaagcc cgccaggtcg ccgtcgtccc

7561 cgtacgcgac gaacgtccgg gagtccgggt cgccgtcctc cccgtcgtcc gattcgtcgt

7621 ccgattcgtc gtcggggaac accttggtca ggggcgggtc caccggcacc tcccgcaggg

7681 tgaagccgtc cccggtggcg gtgacgcgga agacggtgtc ggtggtgaag gacccatcca

7741 gtgcctcgat ggcctcggcg tcccccggga cactggtgcg gtaccggtaa gccgtgtcgt

7801 cgagggtggc catgagtcta gagcttgggt agaataggta agtcagattg aatctgaaat

7861 aaagggagga agggcgaact taagaaggta tgaccgggtc gttcacttac cttgcttgac

7921 aaacgcacca agttatcgtg caccaagcag cagatgataa taatgtcctc gttcctgtct

7981 gctaataaga gtcacacttc gagcgccgcc gctactgcta caagtggggc tgatctgacc

8041 agttgcctaa atgaaccatc ttgtcaaacg acacaaattt tgtgctcacc gcctggacga

8101 ctaaaccaaa ataggcattc attgttgacc tccactagct ccagccaagc ccaaaaagtg

8161 ctccttcaat atcactcgag gaggttttaa gtaactgaga ggtggtttag atatggttgt

8221 agtttttgcc attgaactgc gtctaaaata atgggagaga ggatgtttga atatagtatc

8281 tataaagtat ttcaataaag tattattata aagtaggtgc cagatgtatc agtgagtctg

8341 agtaagaaca tgccaggatt tgaaagtttt atatttctaa acctagctct tgtaactatt

8401 tagcttgggc tacttatcgt gaaagctcta ttttgtcaac cttactagcg aatcctcgct

8461 ttagcatgat tgaaatttga tggatatgtt cgatagctcc cgctcgctta gcaacaatta

8521 ttaatccaat gcggctgcag cagctgaagc ttggcgtaat catggtcata gctgtttcct

8581 gtgtgaaatt gttatccgct cacaattcca cacaacatac gagccggaag cataaagtgt

8641 aaagcctggg gtgcctaatg agtgagctaa ctcacattaa ttgcgttgcg ctcactgccc

8701 gctttccagt cgggaaacct gtcgtgccag ctgcattaat gaatcggcca acgcgcgggg

8761 agaggcggtt tgcgtattgg gcgctcttcc gcttcctcgc tcactgactc gctgcgctcg

8821 gtcgttcggc tgcggcgagc ggtatcagct cactcaaagg cggtaatacg gttatccaca

8881 gaatcagggg ataacgcagg aaagaacatg tgagcaaaag gccagcaaaa ggccaggaac

8941 cgtaaaaagg ccgcgttgct ggcgtttttc cataggctcc gcccccctga cgagcatcac

9001 aaaaatcgac gctcaagtca gaggtggcga aacccgacag gactataaag ataccaggcg

9061 tttccccctg gaagctccct cgtgcgctct cctgttccga ccctgccgct taccggatac

9121 ctgtccgcct ttctcccttc gggaagcgtg gcgctttctc atagctcacg ctgtaggtat

9181 ctcagttcgg tgtaggtcgt tcgctccaag ctgggctgtg tgcacgaacc ccccgttcag

9241 cccgaccgct gcgccttatc cggtaactat cgtcttgagt ccaacccggt aagacacgac

9301 ttatcgccac tggcagcagc cactggtaac aggattagca gagcgaggta tgtaggcggt

9361 gctacagagt tcttgaagtg gtggcctaac tacggctaca ctagaagaac agtatttggt

9421 atctgcgctc tgctgaagcc agttaccttc ggaaaaagag ttggtagctc ttgatccggc

9481 aaacaaacca ccgctggtag cggtggtttt tttgtttgca agcagcagat tacgcgcaga

9541 aaaaaaggat ctcaagaaga tcctttgatc ttttctacgg ggtctgacgc tcagtggaac

9601 gaaaactcac gttaagggat tttggtcatg agattatcaa aaaggatctt cacctagatc

9661 cttttaaatt aaaaatgaag ttttaaatca atctaaagta tatatgagta aacttggtct

9721 gacagttacc aatgcttaat cagtgaggca cctatctcag cgatctgtct atttcgttca

9781 tccatagttg cctgactccc cgtcgtgtag ataactacga tacgggaggg cttaccatct

9841 ggccccagtg ctgcaatgat accgcgagac ccacgctcac cggctccaga tttatcagca

9901 ataaaccagc cagccggaag ggccgagcgc agaagtggtc ctgcaacttt atccgcctcc

9961 atccagtcta ttaattgttg ccgggaagct agagtaagta gttcgccagt taatagtttg

10021 cgcaacgttg ttgccattgc tacaggcatc gtggtgtcac gctcgtcgtt tggtatggct

10081 tcattcagct ccggttccca acgatcaagg cgagttacat gatcccccat gttgtgcaaa

10141 aaagcggtta gctccttcgg tcctccgatc gttgtcagaa gtaagttggc cgcagtgtta

10201 tcactcatgg ttatggcagc actgcataat tctcttactg tcatgccatc cgtaagatgc

10261 ttttctgtga ctggtgagta ctcaaccaag tcattctgag aatagtgtat gcggcgaccg

10321 agttgctctt gcccggcgtc aatacgggat aataccgcgc cacatagcag aactttaaaa

10381 gtgctcatca ttggaaaacg ttcttcgggg cgaaaactct caaggatctt accgctgttg

10441 agatccagtt cgatgtaacc cactcgtgca cccaactgat cttcagcatc ttttactttc

10501 accagcgttt ctgggtgagc aaaaacagga aggcaaaatg ccgcaaaaaa gggaataagg

10561 gcgacacgga aatgttgaat actcatactc ttcctttttc aatattattg aagcatttat

10621 cagggttatt gtctcatgag cggatacata tttgaatgta tttagaaaaa taaacaaata

10681 ggggttccgc gcacatttcc ccgaaaagtg ccacctgacg tctaagaaac cattattatc

10741 atgacattaa cctataaaaa taggcgtatc acgaggccct ttcgtc

//

**4. pTEL-BcCas9GFP-NLS-SV40x4**

LOCUS Exported 16174 bp ds-DNA circular SYN 09-DEC-2019

DEFINITION synthetic circular DNA.

ACCESSION .

VERSION .

KEYWORDS .

SOURCE synthetic DNA construct

ORGANISM synthetic DNA construct

REFERENCE 1 (bases 1 to 16174)

AUTHORS Fabian Bietz

TITLE -

JOURNAL -

FEATURES Location/Qualifiers

source 1..16174

/organism="synthetic DNA construct"

/mol_type="other DNA"

source 329..430

/organism="synthetic DNA construct"

/mol_type="other DNA"

misc_feature 93..689

/label=HTEL

/note="Human telomeric region, compare Barreau et al 1998"

gap 328..431

/estimated_length="104"

CDS complement(1035..1850)

/label=KanR

/note="keep under constant selection for stabilisation of

telomere regions"

misc_feature complement(2020..2512)

/label=HTEL

/note="Human telomeric region, compare Barreau et al 1998"

misc_feature complement(3041..3241)

/label=cos

terminator 4049..4548

/label=T gluc

/note="terminator from B. cinerea"

CDS complement(4564..4581)

/codon_start=1

/product="6xHis affinity tag"

/label=6xHis

/translation="HHHHHH"

CDS complement(4594..4620)

/codon_start=1

/product="HA (human influenza hemagglutinin) epitope tag"

/label=HA

/translation="YPYDVPDYA"

CDS complement(4621..4641)

/codon_start=1

/product="nuclear localization signal of SV40 large T

antigen"

/label=SV40 NLS

/translation="PKKKRKV"

CDS complement(4651..4671)

/codon_start=1

/product="nuclear localization signal of SV40 large T

antigen"

/label=SV40 NLS

/translation="PKKKRKV"

CDS complement(4681..4701)

/codon_start=1

/product="nuclear localization signal of SV40 large T

antigen"

/label=SV40 NLS

/translation="PKKKRKV"

CDS complement(4711..4731)

/codon_start=1

/product="nuclear localization signal of SV40 large T

antigen"

/label=SV40 NLS

/translation="PKKKRKV"

CDS complement(4739..5454)

/codon_start=1

/label=BcGFP

/translation="VSKGEELFTGVVPILVELDGDVNGHKFSVSGEGEGDATYGKLTLK

FICTTGKLPVPWPTLVTTLTYGVQCFSRYPDHMKQHDFFKSAMPEGYVQERTIFFKDDG

NYKTRAEVKFEGDTLVNRIELKGIDFKEDGNILGHKLEYNYNSHNVYIMADKQKNGIKV

NFKIRHNIEDGSVQLADHYQQNTPIGDGPVLLPDNHYLSTQSALSKDPNEKRDHMVLLE

FVTAAGITLGMDELYK"

CDS complement(5464..9567)

/codon_start=1

/product="Cas9 (Csn1) endonuclease from the Streptococcus

pyogenes Type II CRISPR/Cas system"

/label=Cas9

/note="generates RNA-guided double strand breaks in DNA"

/translation="MDKKYSIGLDIGTNSVGWAVITDEYKVPSKKFKVLGNTDRHSIKK

NLIGALLFDSGETAEATRLKRTARRRYTRRKNRICYLQEIFSNEMAKVDDSFFHRLEES

FLVEEDKKHERHPIFGNIVDEVAYHEKYPTIYHLRKKLVDSTDKADLRLIYLALAHMIK

FRGHFLIEGDLNPDNSDVDKLFIQLVQTYNQLFEENPINASGVDAKAILSARLSKSRRL

ENLIAQLPGEKKNGLFGNLIALSLGLTPNFKSNFDLAEDAKLQLSKDTYDDDLDNLLAQ

IGDQYADLFLAAKNLSDAILLSDILRVNTEITKAPLSASMIKRYDEHHQDLTLLKALVR

QQLPEKYKEIFFDQSKNGYAGYIDGGASQEEFYKFIKPILEKMDGTEELLVKLNREDLL

RKQRTFDNGSIPHQIHLGELHAILRRQEDFYPFLKDNREKIEKILTFRIPYYVGPLARG

NSRFAWMTRKSEETITPWNFEEVVDKGASAQSFIERMTNFDKNLPNEKVLPKHSLLYEY

FTVYNELTKVKYVTEGMRKPAFLSGEQKKAIVDLLFKTNRKVTVKQLKEDYFKKIECFD

SVEISGVEDRFNASLGTYHDLLKIIKDKDFLDNEENEDILEDIVLTLTLFEDREMIEER

LKTYAHLFDDKVMKQLKRRRYTGWGRLSRKLINGIRDKQSGKTILDFLKSDGFANRNFM

QLIHDDSLTFKEDIQKAQVSGQGDSLHEHIANLAGSPAIKKGILQTVKVVDELVKVMGR

HKPENIVIEMARENQTTQKGQKNSRERMKRIEEGIKELGSQILKEHPVENTQLQNEKLY

LYYLQNGRDMYVDQELDINRLSDYDVDHIVPQSFLKDDSIDNKVLTRSDKNRGKSDNVP

SEEVVKKMKNYWRQLLNAKLITQRKFDNLTKAERGGLSELDKAGFIKRQLVETRQITKH

VAQILDSRMNTKYDENDKLIREVKVITLKSKLVSDFRKDFQFYKVREINNYHHAHDAYL

NAVVGTALIKKYPKLESEFVYGDYKVYDVRKMIAKSEQEIGKATAKYFFYSNIMNFFKT

EITLANGEIRKRPLIETNGETGEIVWDKGRDFATVRKVLSMPQVNIVKKTEVQTGGFSK

ESILPKRNSDKLIARKKDWDPKKYGGFDSPTVAYSVLVVAKVEKGKSKKLKSVKELLGI

TIMERSSFEKNPIDFLEAKGYKEVKKDLIIKLPKYSLFELENGRKRMLASAGELQKGNE

LALPSKYVNFLYLASHYEKLKGSPEDNEQKQLFVEQHKHYLDEIIEQISEFSKRVILAD

ANLDKVLSAYNKHRDKPIREQAENIIHLFTLTNLGAPAAFKYFDTTIDRKRYTSTKEVL

DATLIHQSITGLYETRIDLSQLGGD"

promoter complement(9568..10409)

/label=OliC

/note="promoter from A. nidulans"

terminator 10486..10736

/label=TtrpC

/note="terminator from A. nidulans"

CDS complement(10737..11762)

/codon_start=1

/gene="aph(4)-Ia"

/product="aminoglycoside phosphotransferase from E. coli"

/label=HygR

/note="confers resistance to hygromycin"

/translation="MKKPELTATSVEKFLIEKFDSVSDLMQLSEGEESRAFSFDVGGRG

YVLRVNSCADGFYKDRYVYRHFASAALPIPEVLDIGEFSESLTYCISRRAQGVTLQDLP

ETELPAVLQPVAEAMDAIAAADLSQTSGFGPFGPQGIGQYTTWRDFICAIADPHVYHWQ

TVMDDTVSASVAQALDELMLWAEDCPEVRHLVHADFGSNNVLTDNGRITAVIDWSEAMF

GDSQYEVANIFFWRPWLACMEQQTRYFERRHPELAGSPRLRAYMLRIGLDQLYQSLVDG

NFDDAAWAQGRCDAIVRSGAGTVGRTQIARRSAAVWTDGCVEVLADSGNRRPSTRPRAK

E"

promoter 11763..12532

/label=Pcpc1

/note="promoter from N. crassa"

CDS 13706..13897

/codon_start=1

/gene="rop"

/product="Rop protein, which maintains plasmids at low copy

number"

/label=rop

/translation="MTKQEKTALNMARFIRSQTLTLLEKLNELDADEQADICESLHDHA

DELYRSCLARFGDDGENL"

misc_feature 13999..14139

/label=bom

/note="basis of mobility region from pBR322"

rep_origin complement(14325..14913)

/direction=LEFT

/label=ori

/note="high-copy-number ColE1/pMB1/pBR322/pUC origin of

replication"

CDS complement(15084..15944)

/codon_start=1

/gene="bla"

/product="beta-lactamase"

/label=AmpR

/note="confers resistance to ampicillin, carbenicillin, and

related antibiotics"

/translation="MSIQHFRVALIPFFAAFCLPVFAHPETLVKVKDAEDQLGARVGYI

ELDLNSGKILESFRPEERFPMMSTFKVLLCGAVLSRVDAGQEQLGRRIHYSQNDLVEYS

PVTEKHLTDGMTVRELCSAAITMSDNTAANLLLTTIGGPKELTAFLHNMGDHVTRLDRW

EPELNEAIPNDERDTTMPAAMATTLRKLLTGELLTLASRQQLIDWMEADKVAGPLLRSA

LPAGWFIADKSGAGERGSRGIIAALGPDGKPSRIVVIYTTGSQATMDERNRQIAEIGAS

LIKHW"

ORIGIN

1 gatctaggcc tgcaggatgc tagcttcaga cgtgtctagg gataacaggg taattcgaac

61 ccccgcgccg cctttgcgag ggtggagttg ccttagggtt agggttaggg ttagggttag

121 ggttagggtt agggttaggg ttagggttag ggttagggtt tagggttagg gttagggtta

181 gggttagggt tagggttagg gtcagggtca ggggtagggt caggggtagg gtcaggggta

241 ggggtagggg tagggtcagg gttagggtta gggttagggt tagggttagg gttagggtta

301 gggtcagggt tagggttagg gttagggnnn nnnnnnnnnn nnnnnnnnnn nnnnnnnnnn

361 nnnnnnnnnn nnnnnnnnnn nnnnnnnnnn nnnnnnnnnn nnnnnnnnnn nnnnnnnnnn

421 nnnnnnnnnn naggggtagg ggtaggggta gggttagggt tagggttagg gttagggtta

481 gggttagggt tagggtcagg gtcagggtca ggggtagggg taggggtagg ggtaggggta

541 gggttagggg tagggttagg ggtaggggta ggggtagggt tagggttagg gtttagggtt

601 agggttaggg ttagggttag ggttaggggt tagggttagg gttagggtta gaaggttagg

661 gttagggtta gggttaaggg ttaagggtta ggtgtggggt gtggcgaatt cctcgacctg

721 caagcggccg cttgcagggg ggggggggcg ctgaggtctg cctcgtgaag aaggtgttgc

781 tgactcatac caggcctgaa tcgccccatc atccagccag aaagtgaggg agccacggtt

841 gatgagagct ttgttgtagg tggaccagtt ggtgattttg aacttttgct ttgccacgga

901 acggtctgcg ttgtcgggaa gatgcgtgat ctgatccttc aactcagcaa aagttcgatt

961 tattcaacaa agccgccgtc ccgtcaagtc agcgtaatgc tctgccagtg ttacaaccaa

1021 ttaaccaatt ctgattagaa aaactcatcg agcatcaaat gaaactgcaa tttattcata

1081 tcaggattat caataccata tttttgaaaa agccgtttct gtaatgaagg agaaaactca

1141 ccgaggcagt tccataggat ggcaagatcc tggtatcggt ctgcgattcc gactcgtcca

1201 acatcaatac aacctattaa tttcccctcg tcaaaaataa ggttatcaag tgagaaatca

1261 ccatgagtga cgactgaatc cggtgagaat ggcaaaagct tatgcatttc tttccagact

1321 tgttcaacag gccagccatt acgctcgtca tcaaaatcac tcgcatcaac caaaccgtta

1381 ttcattcgtg attgcgcctg agcgagacga aatacgcgat cgctgttaaa aggacaatta

1441 caaacaggaa tcgaatgcaa ccggcgcagg aacactgcca gcgcatcaac aatattttca

1501 cctgaatcag gatattcttc taatacctgg aatgctgttt tcccggggat cgcagtggtg

1561 agtaaccatg catcatcagg agtacggata aaatgcttga tggtcggaag aggcataaat

1621 tccgtcagcc agtttagtct gaccatctca tctgtaacat cattggcaac gctacctttg

1681 ccatgtttca gaaacaactc tggcgcatcg ggcttcccat acaatcgata gattgtcgca

1741 cctgattgcc cgacattatc gcgagcccat ttatacccat ataaatcagc atccatgttg

1801 gaatttaatc gcggcctcga gcaagacgtt tcccgttgaa tatggctcat aacacccctt

1861 gtattactgt ttatgtaagc agacagtttt attgttcatg atgatatatt tttatcttgt

1921 gcaatgtaac atcagagatt ttgagacaca acgtggcttt cccccccccc cctgcaagcg

1981 gccgcttgca ggtcgaggaa ttcgccacac cccacaccta acccttaacc cttaacccta

2041 accctaaccc taaccctaac cctaacccta accctaaccc ctaaccctaa ccctaaccct

2101 aaccctaacc ctaaacccta accctaaccc tacccctacc cctaccccta accctacccc

2161 taaccctacc cctaccccta cccctacccc tacccctgac cctgaccctg accctaaccc

2221 taaccctaac cctaacccta accctaaccc taaccctacc cctaccccta cccctnnccc

2281 taaccctaac cctaaccctg accctaaccc taaccctaac cctaacccta accctaaccc

2341 taaccctgac cctaccccta cccctacccc tgaccctacc cctgacccta cccctgaccc

2401 tgaccctaac cctaacccta accctaaccc taaccctaac cctaaaccct aaccctaacc

2461 ctaaccctaa ccctaaccct aaccctaacc ctaaccctaa ccctaaccct aaggcaactc

2521 caccctcgca aaggcggcgc gggggttcga attaccctgt tatccctaga tacgtctgct

2581 ttttgttgac ttccattgtt cattccacgg acaaaaacag agaaaggaaa cgacagaggc

2641 caaaaagctc gctttcagca cctgtcgttt cctttctttt cagagggtat tttaaataaa

2701 aacattaagt tatgacgaag aagaacggaa acgccttaaa ccggaaaatt ttcataaata

2761 gcgaaaaccc gcgaggtcgc cgccccgtaa caaggcggat cgccggaaag gacccgcaaa

2821 tgataataat tatcaattgc atactatcga cggcactgct gccagataac accaccgggg

2881 aaacattcca tcatgatggc cgtgcggaca taggaagcca gttcatccat cgctttcttg

2941 tctgctgcca tttgctttgt gacatccagc gccgcacatt cagcagcgtt tttcagcgcg

3001 ttttcgatca acgtttcaat gttggtatca acaccaggtt taactttgaa cttatcggca

3061 ctgacggtta ccttgttctg cgctggctca tcacgcagga taccaaggct gatgttgtag

3121 atattggtca ccggctgagg gttttcgatt gccgctgcgt ggatagcacc atttgcgatc

3181 aggcgtcctt gatgaatgac actccattgc gaataagttc gaaggagacg gtgtcacgaa

3241 tgcgctggtc cagctcggtc gattgccttt tgtgcagcag aggtatcaat ctcaacgcca

3301 aggctcatcg aagcgcaata ttgctgctca ccaaaacgcg tattgaccag gtgttcaacg

3361 gcaaatttct gcccttctga tgtcagaaag gcaaagtgat tttctttctg gtattcagtt

3421 gctgtgtgtc ggtttcagca aaaccaagct cgcgcaattc ggctgtgcag atttagaagg

3481 cagatcacca gacagcaacg gccaacggaa aacagcgcat acagaacatc cgtcgccgcg

3541 ccgacaacgt gataattttt atgacccatg atttatttcc ttttagacgt gagcctgtcg

3601 cacagcaaag ccgccgaaag ttcctcgacc gatgcctcga ccgatgccct tgagagcctt

3661 caacccagtc agctccttcc ggtgggcgcg gggcatgact atcgtcgccg cacttatgac

3721 tgtcttcttt atcatgcaac tcgtaggaca ggtgccggca gcgctctggg tcattttcgg

3781 cgaggaccgc tttcgctgga gcgcgacgat gatcggcctg tcgcttgcgg tattcggaat

3841 cttgcacgcc ctcgctcaag ccttcgtcac tggtcccgcc accaaacgtt tcggcgagaa

3901 gcaggccatt atcgccggca tggcggccga cgcgctgggc tacgtcttgc tggcgttcga

3961 gcttgcatgc tgatcgccgg agttgagaca aatggtgttc aggatctcga taagatacga

4021 tccacttaac gttactgaaa tctccaacat cttgttgggg ggaaggggtt gtcaaatcag

4081 atgtacgtat gatgttctcc tctttgccta gatatgaaac cgaatatcat atggtaacgc

4141 cacatgaggt gtatcatagg aaacgcatgt ggttgtggat ggtcaattcc aaactttcgt

4201 tttctcaaaa acggcgtttg tgctcctgtc aggccacaat accacgtgag ctgaaaactt

4261 taaacaaatg tcctcctact tgtagtttgt tcacattaca gcagaatcaa tgatgaaaaa

4321 cttggttgca caaatacatg gaatcacaat gttggatcaa gattttcaaa gttgaaaacg

4381 gattgagatt taattccgcc ctcttttgtc ttccgctaaa acaccccctt tgagcaaata

4441 cctccacaag ggtccatgct aatacttatg tacaagacga atcagaaacc acttcctcag

4501 ccataatcct tcccctcaac ccctaatcat acatcttatc tacatacgct agagctctta

4561 tcagtggtgg tggtggtggt ggtcgactta tcaagcataa tctggaacat cgtatgggta

4621 taccttccgc tttttcttag gggaacctcc cacttttcgc ttctttttgg gggagccgcc

4681 tactttccgt ttcttctttg gacttccacc tactttgcgc ttcttcttgg ggtcggccgc

4741 tttgtaaagt tcatccattc ccaaggtaat tccggctgct gtgacaaatt cgagaagaac

4801 catgtggtca cgcttttcgt tagggtcctt tgaaagagca gattgagtgg agaggtaatg

4861 gttatctggg agcaagactg gaccatcacc aattggagta ttctgttggt agtgatctgc

4921 caattgaaca gagccatcct caatgttgtg tctgattttg aagttgacct tgattccatt

4981 tttttgcttg tcggccatga tataaacgtt gtgactgttg tagttgtatt caagcttatg

5041 gccgaggata tttccgtctt ccttgaaatc gatgcccttc aactcaatac ggttgacgag

5101 agtatcaccc tcgaacttaa cctcggcacg agtcttataa tttccgtcat ccttaaagaa

5161 gattgttctc tcttggacat atccctctgg cattgcagac ttgaaaaagt catgttgttt

5221 catgtgatct gggtaacggg aaaagcattg aactccgtag gtgagggtgg tgacgagtgt

5281 aggccaaggt acaggcaatt tacctgtggt gcagatgaac ttaagagtca atttaccata

5341 tgtagcgtct ccctcaccct ctccgctgac tgagaactta tggccattga cgtcaccatc

5401 gagttcgacc aagattggaa cgacgccagt gaaaagctct tctcctttac tgacggatcc

5461 ggggtcgccc ccaagttgac tcaagtcgat acgggtttca tagagcccag tgatggattg

5521 atgaataagg gtcgcatcaa gcacctcttt tgtggacgtg tatcgtttac ggtcgattgt

5581 cgtatcaaaa tacttgaacg ctgctggtgc tcccaagtta gtgagggtga agagatgaat

5641 gatattttca gcttgctctc gaatcggctt gtcacggtgt ttgttgtagg ccgacaacac

5701 tttgtcgaga ttagcatcgg cgagaatgac gcgtttactg aactctgaaa tctgttcaat

5761 gatctcatca agataatgct tgtgttgctc aacaaaaagt tgcttctgtt cgttatcttc

5821 agggctaccc ttcaatttct cgtagtggga cgcgagatac aagaagttca cgtactttga

5881 gggaagtgcc aattcattcc ctttctgcaa ctctcctgcc gatgcgagca ttcgttttcg

5941 accgttttca agctcgaaca aagagtactt aggcaactta atgatcaaat ctttcttgac

6001 ttccttatag cctttggctt cgagaaagtc gatgggattc ttctcgaagc tggagcgctc

6061 cataatggtg atcccgagaa gttctttgac cgacttgagt ttcttagact ttcccttctc

6121 aactttcgcg acaacgagaa ccgagtatgc cactgtagga gaatcgaacc ctccatactt

6181 cttggggtcc caatctttct ttcgagcgat gagtttgtca gagttacgtt ttggaagaat

6241 cgattcctta gagaaaccgc ctgtttgcac ctcggttttc ttgacaatat tgacttgcgg

6301 catgctgaga acctttctca cggtagcgaa gtcgcgcccc ttatcccaaa cgatttcacc

6361 agtctcgccg ttagtctcaa tcaaaggtcg tttacgaatt tctccattgg cgagtgtgat

6421 ctccgtctta aagaaattca tgatgttgga gtaaaagaaa tacttagccg tggctttgcc

6481 aatttcctgc tcactcttgg caatcatctt gcggacatcg taaaccttat aatccccgta

6541 gacaaattcg gactccaact tgggatactt cttgatgagc gcagttccca cgactgcgtt

6601 gaggtacgca tcatgtgcat ggtgataatt gttgatctct cgaaccttat aaaattggaa

6661 gtccttgcgg aaatccgaca ccaacttaga cttgagggta atgaccttaa cttcacggat

6721 gagcttatca ttctcgtcgt actttgtgtt catcctggaa tcaagaatct gagcaacgtg

6781 ttttgtgatc tgtcgcgttt ccacgagttg acgtttgatg aagccagcct tatcgagttc

6841 actcaaccct cctctctcgg ctttggtcaa gttgtcaaac ttgcgttgtg taatgagttt

6901 ggcattaagc aattgtcgcc agtagttctt catcttctta accacttcct cgcttggaac

6961 attgtcggat ttgcctctgt tcttatctga gcgagtcaac actttgttat caatgctatc

7021 gtccttaagg aaggattgcg ggacgatatg atcaacatcg tagtccgaca acctgttaat

7081 gtccaactcc tgatcgacgt acatgtcgcg tccattttgc aaatagtaga gatagagttt

7141 ctcattttgg agttgtgtgt tttcaacagg gtgctctttg aggatttgag aaccaagttc

7201 cttaatgcct tcctcgatac gtttcattct ctcgcggctg ttcttctgac ccttttgagt

7261 ggtctgattt tctcgggcca tctcaatcac gatgttttct ggcttatgac ggcccatcac

7321 tttgacaagc tcatcgacaa ctttgacggt ctgaagaatc cctttcttga tcgccggtga

7381 tccagcaagg ttggcgatat gctcgtgaag ggaatcccct tgtcctgaga cttgtgcttt

7441 ctggatgtcc tctttgaacg tcaaggaatc gtcgtgaata agctgcataa aattgcggtt

7501 ggcgaagcca tccgacttaa ggaaatccaa tatagtctta ccagattgct tatctcgaat

7561 gccgttgatg agtttcctgc taaggcgacc ccatccggtg tatctacgtc gtttcaactg

7621 tttcatcacc ttatcgtcga acaaatgggc gtatgttttg agtctttcct cgatcatttc

7681 gcggtcctca aaaagtgtca acgtgaggac aatatcttcc aagatgtcct cattttcctc

7741 gttatcgagg aaatctttgt ccttaatgat cttgagaagg tcgtggtagg ttccgaggga

7801 agcgttaaag cgatcttcca cgccgctgat ctcgacggag tcgaaacatt caatcttctt

7861 gaaataatcc tccttcaact gcttaacagt cactttacga ttggtcttaa aaagcaaatc

7921 gacgatagcc ttcttttgtt cccctgagag gaaggctggt ttcctcattc cctccgtcac

7981 gtacttgact ttagtgagtt cgttatacac ggtgaaatac tcgtagagca acgaatgttt

8041 tggaaggacc ttttcattcg gaaggttctt atcaaaattg gtcattctct caatgaatga

8101 ttgcgcggat gcacctttgt ccacgacttc ctcgaagttc cacggagtga tggtttcctc

8161 gctttttcgg gtcatccaag caaatcggga atttccacgg gccaaaggtc ccacatagta

8221 ggggattcgg aatgtaagaa tcttttcgat cttctcacgg ttgtctttga gaaatgggta

8281 gaaatcttcc tggcgtcgaa ggatggcgtg gagttcgcca agatgaatct ggtgcggtat

8341 ggagccgttg tcgaacgttc gttgtttacg aagcaaatct tcgcgattga gtttgacgag

8401 aagttcctca gtgccgtcca tcttctccaa aatcggtttg ataaacttgt agaactcttc

8461 ttggctcgca ccgccatcaa tataccctgc gtatccgttt ttggattggt caaagaagat

8521 ttctttgtac ttctcaggca attgctgacg cacgagggcc ttaagcaagg tcaaatcttg

8581 atggtgctca tcataccttt tgatcatact agctgacaag ggggctttag taatctcggt

8641 gttgactctc aaaatgtcac tgagaaggat tgcatctgag agattcttag cggcaaggaa

8701 aagatcagcg tactggtcac cgatttgggc aagcaagttg tcgagatcgt catcatacgt

8761 atctttagac aactggagtt tcgcatcctc tgccaagtca aaattacttt tgaagttagg

8821 agtaagacca agtgacaacg caatcaaatt accgaagagg ccgttcttct tttcgccggg

8881 gagttgtgcg ataaggttct ccaatcttct cgacttagac aagcgagccg aaaggatagc

8941 tttggcatcc acgccagaag cattaatagg gttttcctca aagagttgat tgtaggtttg

9001 gaccaactgg atgaagagtt tatcaacgtc agaattatcg gggttgaggt caccctcgat

9061 aagaaaatgg ccgcggaact taatcatgtg cgcgagtgcc aagtagataa gtcgcaaatc

9121 agctttgtcc gtgctatcga caagtttctt acgaagatga tagatagttg ggtacttttc

9181 gtgataggca acctcatcca caatgttacc aaagatcgga tggcgctcgt gtttcttatc

9241 ctcctcgacc aagaaacttt cttcgagtcg atgaaagaat gagtcatcaa ctttagccat

9301 ttcgttgctg aaaatctctt ggaggtagca gatgcgattc ttgcgtcgtg tataacgtct

9361 gcgagccgtt cgtttcaatc gagtagcttc ggcggtttcc ccactatcga agagaagggc

9421 tccaatgaga ttctttttga ttgagtgacg atcggtgttt ccaaggacct taaacttttt

9481 ggatggcacc ttgtattcgt ccgtaatcac agcccaacca accgagttgg taccaatgtc

9541 gagcccgatg ctatatttct tgtccatgga tcgattgtga tgtgatggag ttgagatggg

9601 ggtgaggaga tggatgatgg gaaaggaaga tggactgagg atggaagaag agaagaagag

9661 agagagagaa agtcttccag gagagaaagg gaaccgaaga aaaatgggga ggaaaccggc

9721 cctagcacct aaatacgtct cccgcttaat tttcgccctt ttttcaccaa accctccgcg

9781 tctttcgtgc gctagctgtc ttggggggtg tgtaaaactt gggaacaacc ctacgccgaa

9841 cctcccgtac gaagcccgta cagtgtattc tatccctggc tttcccagcc tggtaagtcg

9901 ggtcccttcg ggacggggcc aaggagactg agtttccggg ttaaccaata atgccgccag

9961 ccgtggagcg gtctgagctg tctatcgtga atccgtgacg ctgaattgct cagtccaagt

10021 cggagacgct ggaatccacc ggttgctcca gccacggcga agaatccact taccttccgg

10081 gcttccgcca gctggcaaca tatttttgag gctgatatag ccttctcctc catcacgata

10141 agccctgact accttgctac gggccaattg caatttgttt gctggttacg ctttacaaaa

10201 ggtggccgtt actgagcaaa aagaaaatga tgtaaaaatt tgcgagtggg tcccatagct

10261 ggatgggtcc gataaaatgg tactgcccca cttagtggca gctcgcgacc agtcacaagc

10321 ccaggaccgc aagctagaat gatagacggc acctcggacg caaatctgac caatccggcc

10381 cgaatcggaa tgcggctcca cagatgcatt tcctcagctg tataagctat tcttctgctt

10441 cgccggagcc tgaagggcgt actagggttg cgaggtccaa tgcattaatg cattgcagat

10501 gagctgtatc tggaagaggt aaacccgaaa cgcgttttat tcttgttgac atggagctat

10561 taaatcacta gaaggcactc tttgctgctt ggacaaatga acgtatctta tcgagatcct

10621 gaacaccatt tgtctcaact ccggagctga catcgacacc aacgatctta tatccagatt

10681 cgtcaagctg tttgatgatt tcagtaacgt taagtggatc ccggtcggca tctactctat

10741 tcctttgccc tcggacgagt gctggggcgt cggtttccac tatcggcgag tacttctaca

10801 cagccatcgg tccagacggc cgcgcttctg cgggcgattt gtgtacgccc gacagtcccg

10861 gctccggatc ggacgattgc gtcgcatcga ccctgcgccc aagctgcatc atcgaaattg

10921 ccgtcaacca agctctgata gagttggtca agaccaatgc ggagcatata cgcccggagg

10981 cgcggcgatc ctgcaagctc cggatgcctc cgctcgaagt agcgcgtctg ctgctccata

11041 caagccaacc acggcctcca gaagaagatg ttggcgacct cgtattggga atccccgaac

11101 atcgcctcgc tccagtcaat gaccgctgtt atgcggccat tgtccgtcag gacattgttg

11161 gagccgaaat ccgcgtgcac gaggtgccgg acttcggggc agtcctcggc ccaaagcatc

11221 agctcatcga gagcctgcgc gacggacgca ctgacggtgt cgtccatcac agtttgccag

11281 tgatacacat ggggatcagc aatcgcgcat atgaaatcac gccatgtagt gtattgaccg

11341 attccttgcg gtccgaatgg gccgaacccg ctcgtctggc taagatcggc cgcagcgatc

11401 gcatccatgg cctccgcgac cggctgcaga acagcgggca gttcggtttc aggcaggtct

11461 tgcaacgtga caccctgtgc acggcgggag atgcaatagg tcaggctctc gctgaattcc

11521 ccaatgtcaa gcacttccgg aatcgggagc gcggccgatg caaagtgccg ataaacataa

11581 cgatctttgt agaaaccatc ggcgcagcta tttacccgca ggacatatcc acgccctcct

11641 acatcgaagc tgaaagcacg agattcttcg ccctccgaga gctgcatcag gtcggagacg

11701 ctgtcgaact tttcgatcag aaacttctcg acagacgtcg cggtgagttc aggctttttc

11761 atatgggtac ctgagaacat cttgttgccc tgctttccgt gcgaaatact accggtactt

11821 ttgggaaaca agggaacagg agggcgctgc tgtgcgcggt tctgagtgtt caggattgaa

11881 gctgaagaag gtgctgagga agcgtagaac tgttgcggac gcgagttctg agaagagctg

11941 taccgattgg tgaaagccga agaagtgagt tggtgccctg ttgcctggat aatgtttgca

12001 actcgctggt tctgcagaga cggagacaaa tgctggctac gatgttgctg attcaggttg

12061 atacctcggt cgagatactg ttttggtttg atagggtgga tttggttgca gagaagaaga

12121 aaggaaggtc aaagagggaa aactgggcgg agggaaggat tttgtatcag gcagcaaact

12181 gccactgcag tggccctggc agtgccgggc gaggcaccca cgcacggccg cgcaaccggt

12241 tggtccttgc ccaccacgaa acccttctga aaggtcagat ggaagtgtgc gacagtgcgc

12301 gtccccaagc caatgcaggc gccatggatc cactccccac ccgcaagatt tcactgtgcg

12361 ttcttattgg ttgccgcaag gccagccaaa gggggaagta tgagtcacag caccgataca

12421 agaaaattgc agaactaaca tatggatgcg cgcgctattc tgtagagctc tgggcaaagc

12481 accaatcctg cgggtcggta cacacactag cactgcccca cctgaggcag tcagccccgc

12541 tgaccgaatt gccaagagcc aatggagacg gaaagccaac gctgatggag caccatctga

12601 atggacctcg ctcgcttgcc tggaagggac aagggacacc ggagacaggg cctccaacca

12661 agggcgcggg aaagacgatc cccaaagtcg caacggccca gaaagaacgc atccatcaca

12721 ttcggatggg atggcttgaa gccagcttgc agcaacttca aagctcgacg cgaggctgga

12781 tggccttccc cattatgatt cttctcgctt ccggcggcat cgggatgccc gcgttgcagg

12841 ccatgctgtc caggcaggta gatgacgacc atcagggaca gcttcaagga tcgctcgcgg

12901 ctcttaccag cctaacttcg atcattggac cgctgatcgt cacggcgatt tatgccgcct

12961 cggcgagcac atggaacggg ttggcatgga ttgtaggcgc cgccctatac cttgtctgcc

13021 tccccgcgtt gcgtcgcggt gcatggagcc gggccacctc gacctgaatg gaagccggcg

13081 gcacctcgct aacggattca ccactccaag aattggagcc aatcaattct tgcggagaac

13141 tgtgaatgcg caaaccaacc cttggcagaa catatccatc gcgtccgcca tctccagcag

13201 ccgcacgcgg cgcatctcgg gcagcgttgg gtcctggcca cgggtgcgca tgatcgtgct

13261 cctgtcgttg aggacccggc taggctggcg gggttgcctt actggttagc agaatgaatc

13321 accgatacgc gagcgaacgt gaagcgactg ctgctgcaaa acgtctgcga cctgagcaac

13381 aacatgaatg gtcttcggtt tccgtgtttc gtaaagtctg gaaacgcgga agtcagcgcc

13441 ctgcaccatt atgttccgga tctgcatcgc aggatgctgc tggctaccct gtggaacacc

13501 tacatctgta ttaacgaagc gctggcattg accctgagtg atttttctct ggtcccgccg

13561 catccatacc gccagttgtt taccctcaca acgttccagt aaccgggcat gttcatcatc

13621 agtaacccgt atcgtgagca tcctctctcg tttcatcggt atcattaccc ccatgaacag

13681 aaatccccct tacacggagg catcagtgac caaacaggaa aaaaccgccc ttaacatggc

13741 ccgctttatc agaagccaga cattaacgct tctggagaaa ctcaacgagc tggacgcgga

13801 tgaacaggca gacatctgtg aatcgcttca cgaccacgct gatgagcttt accgcagctg

13861 cctcgcgcgt ttcggtgatg acggtgaaaa cctctgacac atgcagctcc cggagacggt

13921 cacagcttgt ctgtaagcgg atgccgggag cagacaagcc cgtcagggcg cgtcagcggg

13981 tgttggcggg tgtcggggcg cagccatgac ccagtcacgt agcgatagcg gagtgtatac

14041 tggcttaact atgcggcatc agagcagatt gtactgagag tgcaccatat gcggtgtgaa

14101 ataccgcaca gatgcgtaag gagaaaatac cgcatcaggc gctcttccgc ttcctcgctc

14161 actgactcgc tgcgctcggt cgttcggctg cggcgagcgg tatcagctca ctcaaaggcg

14221 gtaatacggt tatccacaga atcaggggat aacgcaggaa agaacatgtg agcaaaaggc

14281 cagcaaaagg ccaggaaccg taaaaaggcc gcgttgctgg cgtttttcca taggctccgc

14341 ccccctgacg agcatcacaa aaatcgacgc tcaagtcaga ggtggcgaaa cccgacagga

14401 ctataaagat accaggcgtt tccccctgga agctccctcg tgcgctctcc tgttccgacc

14461 ctgccgctta ccggatacct gtccgccttt ctcccttcgg gaagcgtggc gctttctcat

14521 agctcacgct gtaggtatct cagttcggtg taggtcgttc gctccaagct gggctgtgtg

14581 cacgaacccc ccgttcagcc cgaccgctgc gccttatccg gtaactatcg tcttgagtcc

14641 aacccggtaa gacacgactt atcgccactg gcagcagcca ctggtaacag gattagcaga

14701 gcgaggtatg taggcggtgc tacagagttc ttgaagtggt ggcctaacta cggctacact

14761 agaaggacag tatttggtat ctgcgctctg ctgaagccag ttaccttcgg aaaaagagtt

14821 ggtagctctt gatccggcaa acaaaccacc gctggtagcg gtggtttttt tgtttgcaag

14881 cagcagatta cgcgcagaaa aaaaggatct caagaagatc ctttgatctt ttctacgggg

14941 tctgacgctc agtggaacga aaactcacgt taagggattt tggtcatgag attatcaaaa

15001 aggatcttca cctagatcct tttaaattaa aaatgaagtt ttaaatcaat ctaaagtata

15061 tatgagtaaa cttggtctga cagttaccaa tgcttaatca gtgaggcacc tatctcagcg

15121 atctgtctat ttcgttcatc catagttgcc tgactccccg tcgtgtagat aactacgata

15181 cgggagggct taccatctgg ccccagtgct gcaatgatac cgcgagaccc acgctcaccg

15241 gctccagatt tatcagcaat aaaccagcca gccggaaggg ccgagcgcag aagtggtcct

15301 gcaactttat ccgcctccat ccagtctatt aattgttgcc gggaagctag agtaagtagt

15361 tcgccagtta atagtttgcg caacgttgtt gccattgctg caggcatcgt ggtgtcacgc

15421 tcgtcgtttg gtatggcttc attcagctcc ggttcccaac gatcaaggcg agttacatga

15481 tcccccatgt tgtgcaaaaa agcggttagc tccttcggtc ctccgatcgt tgtcagaagt

15541 aagttggccg cagtgttatc actcatggtt atggcagcac tgcataattc tcttactgtc

15601 atgccatccg taagatgctt ttctgtgact ggtgagtact caaccaagtc attctgagaa

15661 tagtgtatgc ggcgaccgag ttgctcttgc ccggcgtcaa cacgggataa taccgcgcca

15721 catagcagaa ctttaaaagt gctcatcatt ggaaaacgtt cttcggggcg aaaactctca

15781 aggatcttac cgctgttgag atccagttcg atgtaaccca ctcgtgcacc caactgatct

15841 tcagcatctt ttactttcac cagcgtttct gggtgagcaa aaacaggaag gcaaaatgcc

15901 gcaaaaaagg gaataagggc gacacggaaa tgttgaatac tcatactctt cctttttcaa

15961 tattattgaa gcatttatca gggttattgt ctcatgagcg gatacatatt tgaatgtatt

16021 tagaaaaata aacaaatagg ggttccgcgc acatttcccc gaaaagtgcc acctgacgtc

16081 taagaaacca ttattatcat gacattaacc tataaaaata ggcgtatcac gaggcccttt

16141 cgtcttcaag aattcgcggc cccgcatggg ccca

//

**5. pTEL-BcCas9GFP-NLS-Stux2**

LOCUS Exported 16267 bp ds-DNA circular SYN 11-JUN-2019

DEFINITION synthetic circular DNA.

ACCESSION .

VERSION .

KEYWORDS .

SOURCE synthetic DNA construct

ORGANISM synthetic DNA construct

REFERENCE 1 (bases 1 to 16267)

AUTHORS Fabian Bietz

TITLE -

JOURNAL -

FEATURES Location/Qualifiers

source 1..16267

/organism="synthetic DNA construct"

/mol_type="other DNA"

misc_feature 93..689

/label=HTEL

/note="Human telomeric region, compare Barreau et al 1998"

gap 328..431

/estimated_length="104"

CDS complement(1035..1850)

/label=KanR

/note="keep under constant selection for stabilisation of

telomere regions"

misc_feature complement(2020..2512)

/label=HTEL

/note="Human telomeric region, compare Barreau et al 1998"

misc_feature complement(3041..3241)

/label=cos

promoter 3984..4825

/label=OliC

/note="promoter from A. nidulans"

CDS 4826..8929

/codon_start=1

/product="Cas9 (Csn1) endonuclease from the Streptococcus

pyogenes Type II CRISPR/Cas system"

/label=Cas9

/note="generates RNA-guided double strand breaks in DNA"

/translation="MDKKYSIGLDIGTNSVGWAVITDEYKVPSKKFKVLGNTDRHSIKK

NLIGALLFDSGETAEATRLKRTARRRYTRRKNRICYLQEIFSNEMAKVDDSFFHRLEES

FLVEEDKKHERHPIFGNIVDEVAYHEKYPTIYHLRKKLVDSTDKADLRLIYLALAHMIK

FRGHFLIEGDLNPDNSDVDKLFIQLVQTYNQLFEENPINASGVDAKAILSARLSKSRRL

ENLIAQLPGEKKNGLFGNLIALSLGLTPNFKSNFDLAEDAKLQLSKDTYDDDLDNLLAQ

IGDQYADLFLAAKNLSDAILLSDILRVNTEITKAPLSASMIKRYDEHHQDLTLLKALVR

QQLPEKYKEIFFDQSKNGYAGYIDGGASQEEFYKFIKPILEKMDGTEELLVKLNREDLL

RKQRTFDNGSIPHQIHLGELHAILRRQEDFYPFLKDNREKIEKILTFRIPYYVGPLARG

NSRFAWMTRKSEETITPWNFEEVVDKGASAQSFIERMTNFDKNLPNEKVLPKHSLLYEY

FTVYNELTKVKYVTEGMRKPAFLSGEQKKAIVDLLFKTNRKVTVKQLKEDYFKKIECFD

SVEISGVEDRFNASLGTYHDLLKIIKDKDFLDNEENEDILEDIVLTLTLFEDREMIEER

LKTYAHLFDDKVMKQLKRRRYTGWGRLSRKLINGIRDKQSGKTILDFLKSDGFANRNFM

QLIHDDSLTFKEDIQKAQVSGQGDSLHEHIANLAGSPAIKKGILQTVKVVDELVKVMGR

HKPENIVIEMARENQTTQKGQKNSRERMKRIEEGIKELGSQILKEHPVENTQLQNEKLY

LYYLQNGRDMYVDQELDINRLSDYDVDHIVPQSFLKDDSIDNKVLTRSDKNRGKSDNVP

SEEVVKKMKNYWRQLLNAKLITQRKFDNLTKAERGGLSELDKAGFIKRQLVETRQITKH

VAQILDSRMNTKYDENDKLIREVKVITLKSKLVSDFRKDFQFYKVREINNYHHAHDAYL

NAVVGTALIKKYPKLESEFVYGDYKVYDVRKMIAKSEQEIGKATAKYFFYSNIMNFFKT

EITLANGEIRKRPLIETNGETGEIVWDKGRDFATVRKVLSMPQVNIVKKTEVQTGGFSK

ESILPKRNSDKLIARKKDWDPKKYGGFDSPTVAYSVLVVAKVEKGKSKKLKSVKELLGI

TIMERSSFEKNPIDFLEAKGYKEVKKDLIIKLPKYSLFELENGRKRMLASAGELQKGNE

LALPSKYVNFLYLASHYEKLKGSPEDNEQKQLFVEQHKHYLDEIIEQISEFSKRVILAD

ANLDKVLSAYNKHRDKPIREQAENIIHLFTLTNLGAPAAFKYFDTTIDRKRYTSTKEVL

DATLIHQSITGLYETRIDLSQLGGD"

CDS 8939..9654

/codon_start=1

/label=BcGFP

/translation="VSKGEELFTGVVPILVELDGDVNGHKFSVSGEGEGDATYGKLTLK

FICTTGKLPVPWPTLVTTLTYGVQCFSRYPDHMKQHDFFKSAMPEGYVQERTIFFKDDG

NYKTRAEVKFEGDTLVNRIELKGIDFKEDGNILGHKLEYNYNSHNVYIMADKQKNGIKV

NFKIRHNIEDGSVQLADHYQQNTPIGDGPVLLPDNHYLSTQSALSKDPNEKRDHMVLLE

FVTAAGITLGMDELYK"

CDS 9664..9751

/codon_start=1

/label=BcStu NLS 1

CDS 9776..9862

/codon_start=1

/product="putative NLS from B. cinerea BofuT4_P105010.1

(Similar to cell pattern formation-associated protein

stuA)"

/label=BcStu NLS 2

CDS 9866..9892

/codon_start=1

/product="HA (human influenza hemagglutinin) epitope tag"

/label=HA

CDS 9905..9922

/codon_start=1

/product="6xHis affinity tag"

/label=6xHis

terminator 9938..10437

/label=T gluc

/note="terminator from B. cinerea"

terminator 10579..10829

/label=TtrpC

/note="terminator from A. nidulans"

CDS complement(10830..11855)

/codon_start=1

/gene="aph(4)-Ia"

/product="aminoglycoside phosphotransferase from E. coli"

/label=HygR

/note="confers resistance to hygromycin"

promoter 11856..12625

/label=Pcpc1

/note="promoter from N. crassa"

CDS 13799..13990

/codon_start=1

/gene="rop"

/product="Rop protein, which maintains plasmids at low copy

number"

/label=rop

misc_feature 14092..14232

/label=bom

/note="basis of mobility region from pBR322"

rep_origin complement(14418..15006)

/direction=LEFT

/label=ori

/note="high-copy-number ColE1/pMB1/pBR322/pUC origin of

replication"

CDS complement(15177..16037)

/codon_start=1

/gene="bla"

/product="beta-lactamase"

/label=AmpR

/note="confers resistance to ampicillin, carbenicillin, and

related antibiotics"

ORIGIN

1 gatctaggcc tgcaggatgc tagcttcaga cgtgtctagg gataacaggg taattcgaac

61 ccccgcgccg cctttgcgag ggtggagttg ccttagggtt agggttaggg ttagggttag

121 ggttagggtt agggttaggg ttagggttag ggttagggtt tagggttagg gttagggtta

181 gggttagggt tagggttagg gtcagggtca ggggtagggt caggggtagg gtcaggggta

241 ggggtagggg tagggtcagg gttagggtta gggttagggt tagggttagg gttagggtta

301 gggtcagggt tagggttagg gttagggnnn nnnnnnnnnn nnnnnnnnnn nnnnnnnnnn

361 nnnnnnnnnn nnnnnnnnnn nnnnnnnnnn nnnnnnnnnn nnnnnnnnnn nnnnnnnnnn

421 nnnnnnnnnn naggggtagg ggtaggggta gggttagggt tagggttagg gttagggtta

481 gggttagggt tagggtcagg gtcagggtca ggggtagggg taggggtagg ggtaggggta

541 gggttagggg tagggttagg ggtaggggta ggggtagggt tagggttagg gtttagggtt

601 agggttaggg ttagggttag ggttaggggt tagggttagg gttagggtta gaaggttagg

661 gttagggtta gggttaaggg ttaagggtta ggtgtggggt gtggcgaatt cctcgacctg

721 caagcggccg cttgcagggg ggggggggcg ctgaggtctg cctcgtgaag aaggtgttgc

781 tgactcatac caggcctgaa tcgccccatc atccagccag aaagtgaggg agccacggtt

841 gatgagagct ttgttgtagg tggaccagtt ggtgattttg aacttttgct ttgccacgga

901 acggtctgcg ttgtcgggaa gatgcgtgat ctgatccttc aactcagcaa aagttcgatt

961 tattcaacaa agccgccgtc ccgtcaagtc agcgtaatgc tctgccagtg ttacaaccaa

1021 ttaaccaatt ctgattagaa aaactcatcg agcatcaaat gaaactgcaa tttattcata

1081 tcaggattat caataccata tttttgaaaa agccgtttct gtaatgaagg agaaaactca

1141 ccgaggcagt tccataggat ggcaagatcc tggtatcggt ctgcgattcc gactcgtcca

1201 acatcaatac aacctattaa tttcccctcg tcaaaaataa ggttatcaag tgagaaatca

1261 ccatgagtga cgactgaatc cggtgagaat ggcaaaagct tatgcatttc tttccagact

1321 tgttcaacag gccagccatt acgctcgtca tcaaaatcac tcgcatcaac caaaccgtta

1381 ttcattcgtg attgcgcctg agcgagacga aatacgcgat cgctgttaaa aggacaatta

1441 caaacaggaa tcgaatgcaa ccggcgcagg aacactgcca gcgcatcaac aatattttca

1501 cctgaatcag gatattcttc taatacctgg aatgctgttt tcccggggat cgcagtggtg

1561 agtaaccatg catcatcagg agtacggata aaatgcttga tggtcggaag aggcataaat

1621 tccgtcagcc agtttagtct gaccatctca tctgtaacat cattggcaac gctacctttg

1681 ccatgtttca gaaacaactc tggcgcatcg ggcttcccat acaatcgata gattgtcgca

1741 cctgattgcc cgacattatc gcgagcccat ttatacccat ataaatcagc atccatgttg

1801 gaatttaatc gcggcctcga gcaagacgtt tcccgttgaa tatggctcat aacacccctt

1861 gtattactgt ttatgtaagc agacagtttt attgttcatg atgatatatt tttatcttgt

1921 gcaatgtaac atcagagatt ttgagacaca acgtggcttt cccccccccc cctgcaagcg

1981 gccgcttgca ggtcgaggaa ttcgccacac cccacaccta acccttaacc cttaacccta

2041 accctaaccc taaccctaac cctaacccta accctaaccc ctaaccctaa ccctaaccct

2101 aaccctaacc ctaaacccta accctaaccc tacccctacc cctaccccta accctacccc

2161 taaccctacc cctaccccta cccctacccc tacccctgac cctgaccctg accctaaccc

2221 taaccctaac cctaacccta accctaaccc taaccctacc cctaccccta cccctnnccc

2281 taaccctaac cctaaccctg accctaaccc taaccctaac cctaacccta accctaaccc

2341 taaccctgac cctaccccta cccctacccc tgaccctacc cctgacccta cccctgaccc

2401 tgaccctaac cctaacccta accctaaccc taaccctaac cctaaaccct aaccctaacc

2461 ctaaccctaa ccctaaccct aaccctaacc ctaaccctaa ccctaaccct aaggcaactc

2521 caccctcgca aaggcggcgc gggggttcga attaccctgt tatccctaga tacgtctgct

2581 ttttgttgac ttccattgtt cattccacgg acaaaaacag agaaaggaaa cgacagaggc

2641 caaaaagctc gctttcagca cctgtcgttt cctttctttt cagagggtat tttaaataaa

2701 aacattaagt tatgacgaag aagaacggaa acgccttaaa ccggaaaatt ttcataaata

2761 gcgaaaaccc gcgaggtcgc cgccccgtaa caaggcggat cgccggaaag gacccgcaaa

2821 tgataataat tatcaattgc atactatcga cggcactgct gccagataac accaccgggg

2881 aaacattcca tcatgatggc cgtgcggaca taggaagcca gttcatccat cgctttcttg

2941 tctgctgcca tttgctttgt gacatccagc gccgcacatt cagcagcgtt tttcagcgcg

3001 ttttcgatca acgtttcaat gttggtatca acaccaggtt taactttgaa cttatcggca

3061 ctgacggtta ccttgttctg cgctggctca tcacgcagga taccaaggct gatgttgtag

3121 atattggtca ccggctgagg gttttcgatt gccgctgcgt ggatagcacc atttgcgatc

3181 aggcgtcctt gatgaatgac actccattgc gaataagttc gaaggagacg gtgtcacgaa

3241 tgcgctggtc cagctcggtc gattgccttt tgtgcagcag aggtatcaat ctcaacgcca

3301 aggctcatcg aagcgcaata ttgctgctca ccaaaacgcg tattgaccag gtgttcaacg

3361 gcaaatttct gcccttctga tgtcagaaag gcaaagtgat tttctttctg gtattcagtt

3421 gctgtgtgtc ggtttcagca aaaccaagct cgcgcaattc ggctgtgcag atttagaagg

3481 cagatcacca gacagcaacg gccaacggaa aacagcgcat acagaacatc cgtcgccgcg

3541 ccgacaacgt gataattttt atgacccatg atttatttcc ttttagacgt gagcctgtcg

3601 cacagcaaag ccgccgaaag ttcctcgacc gatgcctcga ccgatgccct tgagagcctt

3661 caacccagtc agctccttcc ggtgggcgcg gggcatgact atcgtcgccg cacttatgac

3721 tgtcttcttt atcatgcaac tcgtaggaca ggtgccggca gcgctctggg tcattttcgg

3781 cgaggaccgc tttcgctgga gcgcgacgat gatcggcctg tcgcttgcgg tattcggaat

3841 cttgcacgcc ctcgctcaag ccttcgtcac tggtcccgcc accaaacgtt tcggcgagaa

3901 gcaggccatt atcgccggca tggcggccga cgcgctgggc tacgtcttgc tggcgttcga

3961 gcttgcatgc tgatgctgag gaaatgcatc tgtggagccg cattccgatt cgggccggat

4021 tggtcagatt tgcgtccgag gtgccgtcta tcattctagc ttgcggtcct gggcttgtga

4081 ctggtcgcga gctgccacta agtggggcag taccatttta tcggacccat ccagctatgg

4141 gacccactcg caaattttta catcattttc tttttgctca gtaacggcca ccttttgtaa

4201 agcgtaacca gcaaacaaat tgcaattggc ccgtagcaag gtagtcaggg cttatcgtga

4261 tggaggagaa ggctatatca gcctcaaaaa tatgttgcca gctggcggaa gcccggaagg

4321 taagtggatt cttcgccgtg gctggagcaa ccggtggatt ccagcgtctc cgacttggac

4381 tgagcaattc agcgtcacgg attcacgata gacagctcag accgctccac ggctggcggc

4441 attattggtt aacccggaaa ctcagtctcc ttggccccgt cccgaaggga cccgacttac

4501 caggctggga aagccaggga tagaatacac tgtacgggct tcgtacggga ggttcggcgt

4561 agggttgttc ccaagtttta cacacccccc aagacagcta gcgcacgaaa gacgcggagg

4621 gtttggtgaa aaaagggcga aaattaagcg ggagacgtat ttaggtgcta gggccggttt

4681 cctccccatt tttcttcggt tccctttctc tcctggaaga ctttctctct ctctcttctt

4741 ctcttcttcc atcctcagtc catcttcctt tcccatcatc catctcctca cccccatctc

4801 aactccatca catcacaatc gatccatgga caagaaatat agcatcgggc tcgacattgg

4861 taccaactcg gttggttggg ctgtgattac ggacgaatac aaggtgccat ccaaaaagtt

4921 taaggtcctt ggaaacaccg atcgtcactc aatcaaaaag aatctcattg gagcccttct

4981 cttcgatagt ggggaaaccg ccgaagctac tcgattgaaa cgaacggctc gcagacgtta

5041 tacacgacgc aagaatcgca tctgctacct ccaagagatt ttcagcaacg aaatggctaa

5101 agttgatgac tcattctttc atcgactcga agaaagtttc ttggtcgagg aggataagaa

5161 acacgagcgc catccgatct ttggtaacat tgtggatgag gttgcctatc acgaaaagta

5221 cccaactatc tatcatcttc gtaagaaact tgtcgatagc acggacaaag ctgatttgcg

5281 acttatctac ttggcactcg cgcacatgat taagttccgc ggccattttc ttatcgaggg

5341 tgacctcaac cccgataatt ctgacgttga taaactcttc atccagttgg tccaaaccta

5401 caatcaactc tttgaggaaa accctattaa tgcttctggc gtggatgcca aagctatcct

5461 ttcggctcgc ttgtctaagt cgagaagatt ggagaacctt atcgcacaac tccccggcga

5521 aaagaagaac ggcctcttcg gtaatttgat tgcgttgtca cttggtctta ctcctaactt

5581 caaaagtaat tttgacttgg cagaggatgc gaaactccag ttgtctaaag atacgtatga

5641 tgacgatctc gacaacttgc ttgcccaaat cggtgaccag tacgctgatc ttttccttgc

5701 cgctaagaat ctctcagatg caatccttct cagtgacatt ttgagagtca acaccgagat

5761 tactaaagcc cccttgtcag ctagtatgat caaaaggtat gatgagcacc atcaagattt

5821 gaccttgctt aaggccctcg tgcgtcagca attgcctgag aagtacaaag aaatcttctt

5881 tgaccaatcc aaaaacggat acgcagggta tattgatggc ggtgcgagcc aagaagagtt

5941 ctacaagttt atcaaaccga ttttggagaa gatggacggc actgaggaac ttctcgtcaa

6001 actcaatcgc gaagatttgc ttcgtaaaca acgaacgttc gacaacggct ccataccgca

6061 ccagattcat cttggcgaac tccacgccat ccttcgacgc caggaagatt tctacccatt

6121 tctcaaagac aaccgtgaga agatcgaaaa gattcttaca ttccgaatcc cctactatgt

6181 gggacctttg gcccgtggaa attcccgatt tgcttggatg acccgaaaaa gcgaggaaac

6241 catcactccg tggaacttcg aggaagtcgt ggacaaaggt gcatccgcgc aatcattcat

6301 tgagagaatg accaattttg ataagaacct tccgaatgaa aaggtccttc caaaacattc

6361 gttgctctac gagtatttca ccgtgtataa cgaactcact aaagtcaagt acgtgacgga

6421 gggaatgagg aaaccagcct tcctctcagg ggaacaaaag aaggctatcg tcgatttgct

6481 ttttaagacc aatcgtaaag tgactgttaa gcagttgaag gaggattatt tcaagaagat

6541 tgaatgtttc gactccgtcg agatcagcgg cgtggaagat cgctttaacg cttccctcgg

6601 aacctaccac gaccttctca agatcattaa ggacaaagat ttcctcgata acgaggaaaa

6661 tgaggacatc ttggaagata ttgtcctcac gttgacactt tttgaggacc gcgaaatgat

6721 cgaggaaaga ctcaaaacat acgcccattt gttcgacgat aaggtgatga aacagttgaa

6781 acgacgtaga tacaccggat ggggtcgcct tagcaggaaa ctcatcaacg gcattcgaga

6841 taagcaatct ggtaagacta tattggattt ccttaagtcg gatggcttcg ccaaccgcaa

6901 ttttatgcag cttattcacg acgattcctt gacgttcaaa gaggacatcc agaaagcaca

6961 agtctcagga caaggggatt cccttcacga gcatatcgcc aaccttgctg gatcaccggc

7021 gatcaagaaa gggattcttc agaccgtcaa agttgtcgat gagcttgtca aagtgatggg

7081 ccgtcataag ccagaaaaca tcgtgattga gatggcccga gaaaatcaga ccactcaaaa

7141 gggtcagaag aacagccgcg agagaatgaa acgtatcgag gaaggcatta aggaacttgg

7201 ttctcaaatc ctcaaagagc accctgttga aaacacacaa ctccaaaatg agaaactcta

7261 tctctactat ttgcaaaatg gacgcgacat gtacgtcgat caggagttgg acattaacag

7321 gttgtcggac tacgatgttg atcatatcgt cccgcaatcc ttccttaagg acgatagcat

7381 tgataacaaa gtgttgactc gctcagataa gaacagaggc aaatccgaca atgttccaag

7441 cgaggaagtg gttaagaaga tgaagaacta ctggcgacaa ttgcttaatg ccaaactcat

7501 tacacaacgc aagtttgaca acttgaccaa agccgagaga ggagggttga gtgaactcga

7561 taaggctggc ttcatcaaac gtcaactcgt ggaaacgcga cagatcacaa aacacgttgc

7621 tcagattctt gattccagga tgaacacaaa gtacgacgag aatgataagc tcatccgtga

7681 agttaaggtc attaccctca agtctaagtt ggtgtcggat ttccgcaagg acttccaatt

7741 ttataaggtt cgagagatca acaattatca ccatgcacat gatgcgtacc tcaacgcagt

7801 cgtgggaact gcgctcatca agaagtatcc caagttggag tccgaatttg tctacgggga

7861 ttataaggtt tacgatgtcc gcaagatgat tgccaagagt gagcaggaaa ttggcaaagc

7921 cacggctaag tatttctttt actccaacat catgaatttc tttaagacgg agatcacact

7981 cgccaatgga gaaattcgta aacgaccttt gattgagact aacggcgaga ctggtgaaat

8041 cgtttgggat aaggggcgcg acttcgctac cgtgagaaag gttctcagca tgccgcaagt

8101 caatattgtc aagaaaaccg aggtgcaaac aggcggtttc tctaaggaat cgattcttcc

8161 aaaacgtaac tctgacaaac tcatcgctcg aaagaaagat tgggacccca agaagtatgg

8221 agggttcgat tctcctacag tggcatactc ggttctcgtt gtcgcgaaag ttgagaaggg

8281 aaagtctaag aaactcaagt cggtcaaaga acttctcggg atcaccatta tggagcgctc

8341 cagcttcgag aagaatccca tcgactttct cgaagccaaa ggctataagg aagtcaagaa

8401 agatttgatc attaagttgc ctaagtactc tttgttcgag cttgaaaacg gtcgaaaacg

8461 aatgctcgca tcggcaggag agttgcagaa agggaatgaa ttggcacttc cctcaaagta

8521 cgtgaacttc ttgtatctcg cgtcccacta cgagaaattg aagggtagcc ctgaagataa

8581 cgaacagaag caactttttg ttgagcaaca caagcattat cttgatgaga tcattgaaca

8641 gatttcagag ttcagtaaac gcgtcattct cgccgatgct aatctcgaca aagtgttgtc

8701 ggcctacaac aaacaccgtg acaagccgat tcgagagcaa gctgaaaata tcattcatct

8761 cttcaccctc actaacttgg gagcaccagc agcgttcaag tattttgata cgacaatcga

8821 ccgtaaacga tacacgtcca caaaagaggt gcttgatgcg acccttattc atcaatccat

8881 cactgggctc tatgaaaccc gtatcgactt gagtcaactt gggggcgacc ccggatccgt

8941 cagtaaagga gaagagcttt tcactggcgt cgttccaatc ttggtcgaac tcgatggtga

9001 cgtcaatggc cataagttct cagtcagcgg agagggtgag ggagacgcta catatggtaa

9061 attgactctt aagttcatct gcaccacagg taaattgcct gtaccttggc ctacactcgt

9121 caccaccctc acctacggag ttcaatgctt ttcccgttac ccagatcaca tgaaacaaca

9181 tgactttttc aagtctgcaa tgccagaggg atatgtccaa gagagaacaa tcttctttaa

9241 ggatgacgga aattataaga ctcgtgccga ggttaagttc gagggtgata ctctcgtcaa

9301 ccgtattgag ttgaagggca tcgatttcaa ggaagacgga aatatcctcg gccataagct

9361 tgaatacaac tacaacagtc acaacgttta tatcatggcc gacaagcaaa aaaatggaat

9421 caaggtcaac ttcaaaatca gacacaacat tgaggatggc tctgttcaat tggcagatca

9481 ctaccaacag aatactccaa ttggtgatgg tccagtcttg ctcccagata accattacct

9541 ctccactcaa tctgctcttt caaaggaccc taacgaaaag cgtgaccaca tggttcttct

9601 cgaatttgtc acagcagccg gaattacctt gggaatggat gaactttaca aagcggccgg

9661 aaataaaaga gggcgtgacg acgaagatga cggtaggccg agttcccgag gacctggtat

9721 ggcggacgca gacggactta aacgaagaaa gaccgggtcg agtggtgccg gtaacaagcg

9781 tggcagagat gatgaggacg atggccgacc atccagtaga ggtccaggaa tggccgatgc

9841 tgatggtttg aaaagacgga aaacgtaccc atacgatgtt ccagattatg cttgataagt

9901 cgaccaccac caccaccacc actgataaga gctctagcgt atgtagataa gatgtatgat

9961 taggggttga ggggaaggat tatggctgag gaagtggttt ctgattcgtc ttgtacataa

10021 gtattagcat ggacccttgt ggaggtattt gctcaaaggg ggtgttttag cggaagacaa

10081 aagagggcgg aattaaatct caatccgttt tcaactttga aaatcttgat ccaacattgt

10141 gattccatgt atttgtgcaa ccaagttttt catcattgat tctgctgtaa tgtgaacaaa

10201 ctacaagtag gaggacattt gtttaaagtt ttcagctcac gtggtattgt ggcctgacag

10261 gagcacaaac gccgtttttg agaaaacgaa agtttggaat tgaccatcca caaccacatg

10321 cgtttcctat gatacacctc atgtggcgtt accatatgat attcggtttc atatctaggc

10381 aaagaggaga acatcatacg tacatctgat ttgacaaccc cttcccccca acaagatgtt

10441 ggagatttca gtaacgttaa gtggatcgta tcttatcgag atcctgaaca ccatttgtct

10501 caactccggc gtgtataagc tattcttctg cttcgccgga gcctgaaggg cgtactaggg

10561 ttgcgaggtc caatgcatta atgcattgca gatgagctgt atctggaaga ggtaaacccg

10621 aaacgcgttt tattcttgtt gacatggagc tattaaatca ctagaaggca ctctttgctg

10681 cttggacaaa tgaacgtatc ttatcgagat cctgaacacc atttgtctca actccggagc

10741 tgacatcgac accaacgatc ttatatccag attcgtcaag ctgtttgatg atttcagtaa

10801 cgttaagtgg atcccggtcg gcatctactc tattcctttg ccctcggacg agtgctgggg

10861 cgtcggtttc cactatcggc gagtacttct acacagccat cggtccagac ggccgcgctt

10921 ctgcgggcga tttgtgtacg cccgacagtc ccggctccgg atcggacgat tgcgtcgcat

10981 cgaccctgcg cccaagctgc atcatcgaaa ttgccgtcaa ccaagctctg atagagttgg

11041 tcaagaccaa tgcggagcat atacgcccgg aggcgcggcg atcctgcaag ctccggatgc

11101 ctccgctcga agtagcgcgt ctgctgctcc atacaagcca accacggcct ccagaagaag

11161 atgttggcga cctcgtattg ggaatccccg aacatcgcct cgctccagtc aatgaccgct

11221 gttatgcggc cattgtccgt caggacattg ttggagccga aatccgcgtg cacgaggtgc

11281 cggacttcgg ggcagtcctc ggcccaaagc atcagctcat cgagagcctg cgcgacggac

11341 gcactgacgg tgtcgtccat cacagtttgc cagtgataca catggggatc agcaatcgcg

11401 catatgaaat cacgccatgt agtgtattga ccgattcctt gcggtccgaa tgggccgaac

11461 ccgctcgtct ggctaagatc ggccgcagcg atcgcatcca tggcctccgc gaccggctgc

11521 agaacagcgg gcagttcggt ttcaggcagg tcttgcaacg tgacaccctg tgcacggcgg

11581 gagatgcaat aggtcaggct ctcgctgaat tccccaatgt caagcacttc cggaatcggg

11641 agcgcggccg atgcaaagtg ccgataaaca taacgatctt tgtagaaacc atcggcgcag

11701 ctatttaccc gcaggacata tccacgccct cctacatcga agctgaaagc acgagattct

11761 tcgccctccg agagctgcat caggtcggag acgctgtcga acttttcgat cagaaacttc

11821 tcgacagacg tcgcggtgag ttcaggcttt ttcatatggg tacctgagaa catcttgttg

11881 ccctgctttc cgtgcgaaat actaccggta cttttgggaa acaagggaac aggagggcgc

11941 tgctgtgcgc ggttctgagt gttcaggatt gaagctgaag aaggtgctga ggaagcgtag

12001 aactgttgcg gacgcgagtt ctgagaagag ctgtaccgat tggtgaaagc cgaagaagtg

12061 agttggtgcc ctgttgcctg gataatgttt gcaactcgct ggttctgcag agacggagac

12121 aaatgctggc tacgatgttg ctgattcagg ttgatacctc ggtcgagata ctgttttggt

12181 ttgatagggt ggatttggtt gcagagaaga agaaaggaag gtcaaagagg gaaaactggg

12241 cggagggaag gattttgtat caggcagcaa actgccactg cagtggccct ggcagtgccg

12301 ggcgaggcac ccacgcacgg ccgcgcaacc ggttggtcct tgcccaccac gaaacccttc

12361 tgaaaggtca gatggaagtg tgcgacagtg cgcgtcccca agccaatgca ggcgccatgg

12421 atccactccc cacccgcaag atttcactgt gcgttcttat tggttgccgc aaggccagcc

12481 aaagggggaa gtatgagtca cagcaccgat acaagaaaat tgcagaacta acatatggat

12541 gcgcgcgcta ttctgtagag ctctgggcaa agcaccaatc ctgcgggtcg gtacacacac

12601 tagcactgcc ccacctgagg cagtcagccc cgctgaccga attgccaaga gccaatggag

12661 acggaaagcc aacgctgatg gagcaccatc tgaatggacc tcgctcgctt gcctggaagg

12721 gacaagggac accggagaca gggcctccaa ccaagggcgc gggaaagacg atccccaaag

12781 tcgcaacggc ccagaaagaa cgcatccatc acattcggat gggatggctt gaagccagct

12841 tgcagcaact tcaaagctcg acgcgaggct ggatggcctt ccccattatg attcttctcg

12901 cttccggcgg catcgggatg cccgcgttgc aggccatgct gtccaggcag gtagatgacg

12961 accatcaggg acagcttcaa ggatcgctcg cggctcttac cagcctaact tcgatcattg

13021 gaccgctgat cgtcacggcg atttatgccg cctcggcgag cacatggaac gggttggcat

13081 ggattgtagg cgccgcccta taccttgtct gcctccccgc gttgcgtcgc ggtgcatgga

13141 gccgggccac ctcgacctga atggaagccg gcggcacctc gctaacggat tcaccactcc

13201 aagaattgga gccaatcaat tcttgcggag aactgtgaat gcgcaaacca acccttggca

13261 gaacatatcc atcgcgtccg ccatctccag cagccgcacg cggcgcatct cgggcagcgt

13321 tgggtcctgg ccacgggtgc gcatgatcgt gctcctgtcg ttgaggaccc ggctaggctg

13381 gcggggttgc cttactggtt agcagaatga atcaccgata cgcgagcgaa cgtgaagcga

13441 ctgctgctgc aaaacgtctg cgacctgagc aacaacatga atggtcttcg gtttccgtgt

13501 ttcgtaaagt ctggaaacgc ggaagtcagc gccctgcacc attatgttcc ggatctgcat

13561 cgcaggatgc tgctggctac cctgtggaac acctacatct gtattaacga agcgctggca

13621 ttgaccctga gtgatttttc tctggtcccg ccgcatccat accgccagtt gtttaccctc

13681 acaacgttcc agtaaccggg catgttcatc atcagtaacc cgtatcgtga gcatcctctc

13741 tcgtttcatc ggtatcatta cccccatgaa cagaaatccc ccttacacgg aggcatcagt

13801 gaccaaacag gaaaaaaccg cccttaacat ggcccgcttt atcagaagcc agacattaac

13861 gcttctggag aaactcaacg agctggacgc ggatgaacag gcagacatct gtgaatcgct

13921 tcacgaccac gctgatgagc tttaccgcag ctgcctcgcg cgtttcggtg atgacggtga

13981 aaacctctga cacatgcagc tcccggagac ggtcacagct tgtctgtaag cggatgccgg

14041 gagcagacaa gcccgtcagg gcgcgtcagc gggtgttggc gggtgtcggg gcgcagccat

14101 gacccagtca cgtagcgata gcggagtgta tactggctta actatgcggc atcagagcag

14161 attgtactga gagtgcacca tatgcggtgt gaaataccgc acagatgcgt aaggagaaaa

14221 taccgcatca ggcgctcttc cgcttcctcg ctcactgact cgctgcgctc ggtcgttcgg

14281 ctgcggcgag cggtatcagc tcactcaaag gcggtaatac ggttatccac agaatcaggg

14341 gataacgcag gaaagaacat gtgagcaaaa ggccagcaaa aggccaggaa ccgtaaaaag

14401 gccgcgttgc tggcgttttt ccataggctc cgcccccctg acgagcatca caaaaatcga

14461 cgctcaagtc agaggtggcg aaacccgaca ggactataaa gataccaggc gtttccccct

14521 ggaagctccc tcgtgcgctc tcctgttccg accctgccgc ttaccggata cctgtccgcc

14581 tttctccctt cgggaagcgt ggcgctttct catagctcac gctgtaggta tctcagttcg

14641 gtgtaggtcg ttcgctccaa gctgggctgt gtgcacgaac cccccgttca gcccgaccgc

14701 tgcgccttat ccggtaacta tcgtcttgag tccaacccgg taagacacga cttatcgcca

14761 ctggcagcag ccactggtaa caggattagc agagcgaggt atgtaggcgg tgctacagag

14821 ttcttgaagt ggtggcctaa ctacggctac actagaagga cagtatttgg tatctgcgct

14881 ctgctgaagc cagttacctt cggaaaaaga gttggtagct cttgatccgg caaacaaacc

14941 accgctggta gcggtggttt ttttgtttgc aagcagcaga ttacgcgcag aaaaaaagga

15001 tctcaagaag atcctttgat cttttctacg gggtctgacg ctcagtggaa cgaaaactca

15061 cgttaaggga ttttggtcat gagattatca aaaaggatct tcacctagat ccttttaaat

15121 taaaaatgaa gttttaaatc aatctaaagt atatatgagt aaacttggtc tgacagttac

15181 caatgcttaa tcagtgaggc acctatctca gcgatctgtc tatttcgttc atccatagtt

15241 gcctgactcc ccgtcgtgta gataactacg atacgggagg gcttaccatc tggccccagt

15301 gctgcaatga taccgcgaga cccacgctca ccggctccag atttatcagc aataaaccag

15361 ccagccggaa gggccgagcg cagaagtggt cctgcaactt tatccgcctc catccagtct

15421 attaattgtt gccgggaagc tagagtaagt agttcgccag ttaatagttt gcgcaacgtt

15481 gttgccattg ctgcaggcat cgtggtgtca cgctcgtcgt ttggtatggc ttcattcagc

15541 tccggttccc aacgatcaag gcgagttaca tgatccccca tgttgtgcaa aaaagcggtt

15601 agctccttcg gtcctccgat cgttgtcaga agtaagttgg ccgcagtgtt atcactcatg

15661 gttatggcag cactgcataa ttctcttact gtcatgccat ccgtaagatg cttttctgtg

15721 actggtgagt actcaaccaa gtcattctga gaatagtgta tgcggcgacc gagttgctct

15781 tgcccggcgt caacacggga taataccgcg ccacatagca gaactttaaa agtgctcatc

15841 attggaaaac gttcttcggg gcgaaaactc tcaaggatct taccgctgtt gagatccagt

15901 tcgatgtaac ccactcgtgc acccaactga tcttcagcat cttttacttt caccagcgtt

15961 tctgggtgag caaaaacagg aaggcaaaat gccgcaaaaa agggaataag ggcgacacgg

16021 aaatgttgaa tactcatact cttccttttt caatattatt gaagcattta tcagggttat

16081 tgtctcatga gcggatacat atttgaatgt atttagaaaa ataaacaaat aggggttccg

16141 cgcacatttc cccgaaaagt gccacctgac gtctaagaaa ccattattat catgacatta

16201 acctataaaa ataggcgtat cacgaggccc tttcgtcttc aagaattcgc ggccccgcat

16261 gggccca

//

**6. pET24a_Cas9_SV40x4-NLS_His**

LOCUS Exported 9500 bp ds-DNA circular SYN 21-MAY-2019

DEFINITION synthetic circular DNA.

ACCESSION .

VERSION .

KEYWORDS 1. pET24a_Sp_Cas9_4xSV40_His

SOURCE synthetic DNA construct

ORGANISM synthetic DNA construct

REFERENCE 1 (bases 1 to 9500)

AUTHORS Thomas Leisen

TITLE -

JOURNAL -

FEATURES Location/Qualifiers

source 1..9500

/organism="synthetic DNA construct"

/lab_host="Escherichia coli"

/mol_type="other DNA"

CDS 14..31

/product="6xHis affinity tag"

/label=6xHis

terminator 98..145

/label=T7 terminator

/note="transcription terminator for bacteriophage T7 RNA

polymerase"

rep_origin 182..637

/label=f1 origin

/label=f1\origin

/note="/vntifkey=33"

CDS complement(730..1545)

/gene="aph(3')-Ia"

/product="aminoglycoside phosphotransferase"

/label=KanR

/note="confers resistance to kanamycin in bacteria or G418

(Geneticin(R)) in eukaryotes"

rep_origin 1667..2255

/label=ori

/note="high-copy-number ColE1/pMB1/pBR322/pUC origin of

replication"

CDS complement(3685..4767)

/codon_start=1

/gene="lacI"

/product="lac repressor"

/label=lacI

/note="The lac repressor binds to the lac operator to

inhibit transcription in E. coli. This inhibition can be

relieved by adding lactose or

isopropyl-beta-D-thiogalactopyranoside (IPTG)."

promoter complement(4768..4845)

/gene="lacI"

/label=lacI promoter

promoter 5154..5172

/label=T7 promoter

/note="promoter for bacteriophage T7 RNA polymerase"

CDS 5173..5197

/codon_start=1

/label=lac operator

/label=lac\operator

/note="/vntifkey=4"

/translation="GIVSG*QF"

RBS 5212..5234

/note="efficient ribosome binding site from bacteriophage

T7 gene 10 (Olins and Rangwala, 1989)"

CDS 5242..9345

/codon_start=1

/label=Sp_Cas9

/note="/vntifkey=4"

/translation="MDKKYSIGLDIGTNSVGWAVITDEYKVPSKKFKVLGNTDRHSIKK

NLIGALLFDSGETAEATRLKRTARRRYTRRKNRICYLQEIFSNEMAKVDDSFFHRLEES

FLVEEDKKHERHPIFGNIVDEVAYHEKYPTIYHLRKKLVDSTDKADLRLIYLALAHMIK

FRGHFLIEGDLNPDNSDVDKLFIQLVQTYNQLFEENPINASGVDAKAILSARLSKSRRL

ENLIAQLPGEKKNGLFGNLIALSLGLTPNFKSNFDLAEDAKLQLSKDTYDDDLDNLLAQ

IGDQYADLFLAAKNLSDAILLSDILRVNTEITKAPLSASMIKRYDEHHQDLTLLKALVR

QQLPEKYKEIFFDQSKNGYAGYIDGGASQEEFYKFIKPILEKMDGTEELLVKLNREDLL

RKQRTFDNGSIPHQIHLGELHAILRRQEDFYPFLKDNREKIEKILTFRIPYYVGPLARG

NSRFAWMTRKSEETITPWNFEEVVDKGASAQSFIERMTNFDKNLPNEKVLPKHSLLYEY

FTVYNELTKVKYVTEGMRKPAFLSGEQKKAIVDLLFKTNRKVTVKQLKEDYFKKIECFD

SVEISGVEDRFNASLGTYHDLLKIIKDKDFLDNEENEDILEDIVLTLTLFEDREMIEER

LKTYAHLFDDKVMKQLKRRRYTGWGRLSRKLINGIRDKQSGKTILDFLKSDGFANRNFM

QLIHDDSLTFKEDIQKAQVSGQGDSLHEHIANLAGSPAIKKGILQTVKVVDELVKVMGR

HKPENIVIEMARENQTTQKGQKNSRERMKRIEEGIKELGSQILKEHPVENTQLQNEKLY

LYYLQNGRDMYVDQELDINRLSDYDVDHIVPQSFLKDDSIDNKVLTRSDKNRGKSDNVP

SEEVVKKMKNYWRQLLNAKLITQRKFDNLTKAERGGLSELDKAGFIKRQLVETRQITKH

VAQILDSRMNTKYDENDKLIREVKVITLKSKLVSDFRKDFQFYKVREINNYHHAHDAYL

NAVVGTALIKKYPKLESEFVYGDYKVYDVRKMIAKSEQEIGKATAKYFFYSNIMNFFKT

EITLANGEIRKRPLIETNGETGEIVWDKGRDFATVRKVLSMPQVNIVKKTEVQTGGFSK

ESILPKRNSDKLIARKKDWDPKKYGGFDSPTVAYSVLVVAKVEKGKSKKLKSVKELLGI

TIMERSSFEKNPIDFLEAKGYKEVKKDLIIKLPKYSLFELENGRKRMLASAGELQKGNE

LALPSKYVNFLYLASHYEKLKGSPEDNEQKQLFVEQHKHYLDEIIEQISEFSKRVILAD

ANLDKVLSAYNKHRDKPIREQAENIIHLFTLTNLGAPAAFKYFDTTIDRKRYTSTKEVL

DATLIHQSITGLYETRIDLSQLGGD"

CDS 9367..9387

/codon_start=1

/product="nuclear localization signal of SV40 (simian virus

40) large T antigen"

/label=SV40 NLS

CDS 9397..9417

/codon_start=1

/product="nuclear localization signal of SV40 (simian virus

40) large T antigen"

/label=SV40 NLS

CDS 9427..9447

/codon_start=1

/product="nuclear localization signal of SV40 (simian virus

40) large T antigen"

/label=SV40 NLS

CDS 9457..9477

/codon_start=1

/product="nuclear localization signal of SV40 (simian virus

40) large T antigen"

/label=SV40 NLS

CDS 9478..9495

/codon_start=1

/product="6xHis affinity tag"

/label=6xHis

ORIGIN

1 ggccgcactc gagcaccacc accaccacca ctgagatccg gctgctaaca aagcccgaaa

61 ggaagctgag ttggctgctg ccaccgctga gcaataacta gcataacccc ttggggcctc

121 taaacgggtc ttgaggggtt ttttgctgaa aggaggaact atatccggat tggcgaatgg

181 gacgcgccct gtagcggcgc attaagcgcg gcgggtgtgg tggttacgcg cagcgtgacc

241 gctacacttg ccagcgccct agcgcccgct cctttcgctt tcttcccttc ctttctcgcc

301 acgttcgccg gctttccccg tcaagctcta aatcgggggc tccctttagg gttccgattt

361 agtgctttac ggcacctcga ccccaaaaaa cttgattagg gtgatggttc acgtagtggg

421 ccatcgccct gatagacggt ttttcgccct ttgacgttgg agtccacgtt ctttaatagt

481 ggactcttgt tccaaactgg aacaacactc aaccctatct cggtctattc ttttgattta

541 taagggattt tgccgatttc ggcctattgg ttaaaaaatg agctgattta acaaaaattt

601 aacgcgaatt ttaacaaaat attaacgttt acaatttcag gtggcacttt tcggggaaat

661 gtgcgcggaa cccctatttg tttatttttc taaatacatt caaatatgta tccgctcatg

721 aattaattct tagaaaaact catcgagcat caaatgaaac tgcaatttat tcatatcagg

781 attatcaata ccatattttt gaaaaagccg tttctgtaat gaaggagaaa actcaccgag

841 gcagttccat aggatggcaa gatcctggta tcggtctgcg attccgactc gtccaacatc

901 aatacaacct attaatttcc cctcgtcaaa aataaggtta tcaagtgaga aatcaccatg

961 agtgacgact gaatccggtg agaatggcaa aagtttatgc atttctttcc agacttgttc

1021 aacaggccag ccattacgct cgtcatcaaa atcactcgca tcaaccaaac cgttattcat

1081 tcgtgattgc gcctgagcga gacgaaatac gcgatcgctg ttaaaaggac aattacaaac

1141 aggaatcgaa tgcaaccggc gcaggaacac tgccagcgca tcaacaatat tttcacctga

1201 atcaggatat tcttctaata cctggaatgc tgttttcccg gggatcgcag tggtgagtaa

1261 ccatgcatca tcaggagtac ggataaaatg cttgatggtc ggaagaggca taaattccgt

1321 cagccagttt agtctgacca tctcatctgt aacatcattg gcaacgctac ctttgccatg

1381 tttcagaaac aactctggcg catcgggctt cccatacaat cgatagattg tcgcacctga

1441 ttgcccgaca ttatcgcgag cccatttata cccatataaa tcagcatcca tgttggaatt

1501 taatcgcggc ctagagcaag acgtttcccg ttgaatatgg ctcataacac cccttgtatt

1561 actgtttatg taagcagaca gttttattgt tcatgaccaa aatcccttaa cgtgagtttt

1621 cgttccactg agcgtcagac cccgtagaaa agatcaaagg atcttcttga gatccttttt

1681 ttctgcgcgt aatctgctgc ttgcaaacaa aaaaaccacc gctaccagcg gtggtttgtt

1741 tgccggatca agagctacca actctttttc cgaaggtaac tggcttcagc agagcgcaga

1801 taccaaatac tgtccttcta gtgtagccgt agttaggcca ccacttcaag aactctgtag

1861 caccgcctac atacctcgct ctgctaatcc tgttaccagt ggctgctgcc agtggcgata

1921 agtcgtgtct taccgggttg gactcaagac gatagttacc ggataaggcg cagcggtcgg

1981 gctgaacggg gggttcgtgc acacagccca gcttggagcg aacgacctac accgaactga

2041 gatacctaca gcgtgagcta tgagaaagcg ccacgcttcc cgaagggaga aaggcggaca

2101 ggtatccggt aagcggcagg gtcggaacag gagagcgcac gagggagctt ccagggggaa

2161 acgcctggta tctttatagt cctgtcgggt ttcgccacct ctgacttgag cgtcgatttt

2221 tgtgatgctc gtcagggggg cggagcctat ggaaaaacgc cagcaacgcg gcctttttac

2281 ggttcctggc cttttgctgg ccttttgctc acatgttctt tcctgcgtta tcccctgatt

2341 ctgtggataa ccgtattacc gcctttgagt gagctgatac cgctcgccgc agccgaacga

2401 ccgagcgcag cgagtcagtg agcgaggaag cggaagagcg cctgatgcgg tattttctcc

2461 ttacgcatct gtgcggtatt tcacaccgca tatatggtgc actctcagta caatctgctc

2521 tgatgccgca tagttaagcc agtatacact ccgctatcgc tacgtgactg ggtcatggct

2581 gcgccccgac acccgccaac acccgctgac gcgccctgac gggcttgtct gctcccggca

2641 tccgcttaca gacaagctgt gaccgtctcc gggagctgca tgtgtcagag gttttcaccg

2701 tcatcaccga aacgcgcgag gcagctgcgg taaagctcat cagcgtggtc gtgaagcgat

2761 tcacagatgt ctgcctgttc atccgcgtcc agctcgttga gtttctccag aagcgttaat

2821 gtctggcttc tgataaagcg ggccatgtta agggcggttt tttcctgttt ggtcactgat

2881 gcctccgtgt aagggggatt tctgttcatg ggggtaatga taccgatgaa acgagagagg

2941 atgctcacga tacgggttac tgatgatgaa catgcccggt tactggaacg ttgtgagggt

3001 aaacaactgg cggtatggat gcggcgggac cagagaaaaa tcactcaggg tcaatgccag

3061 cgcttcgtta atacagatgt aggtgttcca cagggtagcc agcagcatcc tgcgatgcag

3121 atccggaaca taatggtgca gggcgctgac ttccgcgttt ccagacttta cgaaacacgg

3181 aaaccgaaga ccattcatgt tgttgctcag gtcgcagacg ttttgcagca gcagtcgctt

3241 cacgttcgct cgcgtatcgg tgattcattc tgctaaccag taaggcaacc ccgccagcct

3301 agccgggtcc tcaacgacag gagcacgatc atgcgcaccc gtggggccgc catgccggcg

3361 ataatggcct gcttctcgcc gaaacgtttg gtggcgggac cagtgacgaa ggcttgagcg

3421 agggcgtgca agattccgaa taccgcaagc gacaggccga tcatcgtcgc gctccagcga

3481 aagcggtcct cgccgaaaat gacccagagc gctgccggca cctgtcctac gagttgcatg

3541 ataaagaaga cagtcataag tgcggcgacg atagtcatgc cccgcgccca ccggaaggag

3601 ctgactgggt tgaaggctct caagggcatc ggtcgagatc ccggtgccta atgagtgagc

3661 taacttacat taattgcgtt gcgctcactg cccgctttcc agtcgggaaa cctgtcgtgc

3721 cagctgcatt aatgaatcgg ccaacgcgcg gggagaggcg gtttgcgtat tgggcgccag

3781 ggtggttttt cttttcacca gtgagacggg caacagctga ttgcccttca ccgcctggcc

3841 ctgagagagt tgcagcaagc ggtccacgct ggtttgcccc agcaggcgaa aatcctgttt

3901 gatggtggtt aacggcggga tataacatga gctgtcttcg gtatcgtcgt atcccactac

3961 cgagatatcc gcaccaacgc gcagcccgga ctcggtaatg gcgcgcattg cgcccagcgc

4021 catctgatcg ttggcaacca gcatcgcagt gggaacgatg ccctcattca gcatttgcat

4081 ggtttgttga aaaccggaca tggcactcca gtcgccttcc cgttccgcta tcggctgaat

4141 ttgattgcga gtgagatatt tatgccagcc agccagacgc agacgcgccg agacagaact

4201 taatgggccc gctaacagcg cgatttgctg gtgacccaat gcgaccagat gctccacgcc

4261 cagtcgcgta ccgtcttcat gggagaaaat aatactgttg atgggtgtct ggtcagagac

4321 atcaagaaat aacgccggaa cattagtgca ggcagcttcc acagcaatgg catcctggtc

4381 atccagcgga tagttaatga tcagcccact gacgcgttgc gcgagaagat tgtgcaccgc

4441 cgctttacag gcttcgacgc cgcttcgttc taccatcgac accaccacgc tggcacccag

4501 ttgatcggcg cgagatttaa tcgccgcgac aatttgcgac ggcgcgtgca gggccagact

4561 ggaggtggca acgccaatca gcaacgactg tttgcccgcc agttgttgtg ccacgcggtt

4621 gggaatgtaa ttcagctccg ccatcgccgc ttccactttt tcccgcgttt tcgcagaaac

4681 gtggctggcc tggttcacca cgcgggaaac ggtctgataa gagacaccgg catactctgc

4741 gacatcgtat aacgttactg gtttcacatt caccaccctg aattgactct cttccgggcg

4801 ctatcatgcc ataccgcgaa aggttttgcg ccattcgatg gtgtccggga tctcgacgct

4861 ctcccttatg cgactcctgc attaggaagc agcccagtag taggttgagg ccgttgagca

4921 ccgccgccgc aaggaatggt gcatgcaagg agatggcgcc caacagtccc ccggccacgg

4981 ggcctgccac catacccacg ccgaaacaag cgctcatgag cccgaagtgg cgagcccgat

5041 cttccccatc ggtgatgtcg gcgatatagg cgccagcaac cgcacctgtg gcgccggtga

5101 tgccggccac gatgcgtccg gcgtagagga tcgagatctc gatcccgcga aattaatacg

5161 actcactata ggggaattgt gagcggataa caattcccct ctagaaataa ttttgtttaa

5221 ctttaagaag gagatataca tatggataag aaatatagca ttggcctgga cattggcacc

5281 aacagcgttg gttgggcagt gattaccgac gagtacaagg ttccgagcaa gaagttcaag

5341 gtgctgggta acaccgatcg ccatagtatc aagaaaaact taatcggcgc cctgctgttt

5401 gatagtggcg agaccgcaga agcaacacgc ctgaaacgca cagcacgccg tcgttataca

5461 cgtcgtaaga atcgcatttg ctatctgcag gaaattttta gtaatgagat ggccaaggtg

5521 gatgatagct tctttcaccg tctggaggag agcttcctgg tggaagagga caagaaacac

5581 gagcgccatc cgatctttgg caatatcgtg gacgaagtgg cctaccatga gaagtatccg

5641 acaatctacc atctgcgcaa gaagctggtt gacagcaccg ataaggccga cctgcgtctg

5701 atctatttag ccctggcaca catgatcaaa tttcgcggcc atttcctgat cgagggtgat

5761 ctgaatccgg acaacagtga cgttgacaag ttatttattc agctggttca aacatacaat

5821 caactgttcg aagaaaatcc gattaatgcc agcggcgtgg atgcaaaggc aatcctgagt

5881 gcccgcctga gcaaaagccg tcgtctggaa aatctgatcg cacagctgcc gggtgagaag

5941 aagaacggcc tgtttggtaa cttaattgca ctgagcctgg gtttaacccc gaactttaaa

6001 agcaacttcg acctggcaga agacgccaag ttacagctga gtaaagacac ctacgacgac

6061 gatctggaca acttattagc ccagatcggt gaccagtatg ccgatctgtt tctggcagca

6121 aagaacctga gcgatgcaat cctgctgagc gacattctgc gtgtgaatac cgagattacc

6181 aaagccccgt taagcgcaag tatgattaag cgctatgatg agcaccacca ggacttaacc

6241 ctgctgaaag ccctggtgcg ccagcagtta cctgagaaat acaaagaaat cttttttgat

6301 caaagtaaga atggctacgc cggttatatc gatggcggtg ccagccaaga ggagttttat

6361 aaattcatca aacctatcct ggagaaaatg gacggcaccg aagaactgct ggttaaatta

6421 aatcgcgaag atttactgcg caagcagcgt accttcgata atggcagcat tccgcaccaa

6481 atccacctgg gcgaattaca tgcaatcctg cgccgtcagg aagattttta cccgtttctg

6541 aaagataatc gtgaaaaaat cgaaaagatc ctgaccttcc gcattccgta ttatgttggt

6601 cctctggccc gtggtaacag tcgctttgca tggatgaccc gcaagagcga ggaaacaatt

6661 accccgtgga atttcgaaga agttgttgac aaaggtgcca gcgcccagag cttcatcgag

6721 cgcatgacca actttgataa gaacctgccg aatgaaaaag ttctgccgaa acatagtctg

6781 ctgtatgagt atttcaccgt gtacaatgag ttaacaaaag ttaaatatgt gacagaaggt

6841 atgcgcaagc cggcctttct gagcggtgag cagaagaagg ccatcgtgga cttactgttt

6901 aagaccaatc gcaaagttac cgttaaacag ctgaaagaag actatttcaa gaaaattgag

6961 tgtttcgata gcgttgaaat tagtggcgtg gaggatcgct tcaacgcaag tctgggcacc

7021 tatcatgatt tactgaagat tattaaggac aaggacttcc tggacaacga agagaacgag

7081 gacattttag aggacatcgt gctgaccctg accctgtttg aagatcgcga gatgattgag

7141 gagcgcctga aaacctacgc acacctgttc gatgacaagg tgatgaagca gctgaaacgt

7201 cgccgctata ccggctgggg tcgcttaagc cgcaagctga tcaacggcat ccgcgacaag

7261 cagagcggca aaaccatcct ggacttcctg aagagcgatg gttttgccaa ccgcaatttc

7321 atgcagctga tccacgacga cagcctgacc tttaaagaag acattcagaa agcacaggtt

7381 agtggtcaag gcgacagcct gcatgaacac atcgcaaacc tggcaggtag cccggccatt

7441 aagaaaggta ttctgcagac cgtgaaggtg gtggatgagc tggttaaggt gatgggccgt

7501 cacaaaccgg aaaatattgt tatcgaaatg gcccgtgaga atcaaacaac ccagaagggc

7561 cagaaaaata gccgcgagcg catgaagcgc attgaggaag gcattaaaga attaggcagc

7621 caaattctga aggagcatcc ggttgaaaac acacagctgc agaatgagaa gctgtacctg

7681 tactacctgc agaatggccg cgacatgtat gttgatcagg aattagatat taatcgtctg

7741 agcgactacg acgttgacca tatcgtgcct cagagctttc tgaaggatga cagcatcgac

7801 aacaaggtgc tgacccgtag cgataagaac cgcggtaaga gcgataacgt tccgagcgaa

7861 gaagttgtta agaaaatgaa aaactattgg cgccagctgt taaacgccaa gttaatcacc

7921 caacgtaagt tcgataatct gacaaaggca gaacgtggtg gcctgagcga gctggacaaa

7981 gccggtttca ttaaacgcca gctggtggag acacgccaga tcaccaaaca cgtggcccaa

8041 atcctggaca gccgtatgaa tacaaagtat gatgaaaatg ataaattaat tcgtgaagtt

8101 aaagttatta ccctgaagag caagttagtg agcgacttcc gtaaggactt ccagttttac

8161 aaagttcgcg aaattaataa ttaccaccat gcacacgatg catacctgaa tgccgttgtt

8221 ggcaccgccc tgattaagaa gtacccgaag ctggagagcg agttcgttta tggtgattac

8281 aaagtgtacg atgtgcgcaa aatgatcgca aagagcgaac aggagattgg caaagccacc

8341 gccaaatact tcttttatag caatattatg aatttcttca aaaccgaaat taccctggcc

8401 aatggcgaga ttcgtaaacg tccgctgatc gaaacaaatg gcgaaacagg cgagatcgtg

8461 tgggataagg gccgtgactt tgcaaccgtt cgcaaggtgc tgagcatgcc gcaggtgaat

8521 attgttaaga aaactgaagt tcaaacaggt ggctttagta aggagagcat tctgcctaaa

8581 cgcaacagcg acaaactgat cgcacgcaag aaagactggg accctaagaa atacggcggc

8641 tttgacagcc cgaccgttgc ctatagtgtg ctggtggttg ccaaggtgga gaagggtaaa

8701 agtaaaaaat taaagagtgt taaagagctg ttaggcatta caatcatgga gcgcagcagt

8761 tttgaaaaga accctatcga cttcctggaa gccaagggtt ataaagaagt taaaaaggac

8821 ctgattatta agctgcctaa atatagctta tttgagttag agaacggccg taaacgcatg

8881 ctggcaagcg ccggtgaact gcaaaagggc aatgagctgg ccctgccgag taagtatgtg

8941 aatttcctgt atttagcaag tcactatgaa aagttaaagg gcagcccgga ggacaacgag

9001 cagaagcagc tgtttgttga acagcacaaa cactacctgg acgagattat cgaacagatc

9061 agtgagttca gtaagcgtgt gatcttagcc gacgcaaacc tggacaaagt gctgagcgca

9121 tataacaagc accgtgacaa accgattcgc gagcaggccg agaacattat tcatttattt

9181 accttaacca atctgggtgc accggcagcc ttcaaatatt ttgacacaac catcgaccgc

9241 aagcgctaca caagcacaaa agaagttctg gacgcaacac tgatccacca gagcatcacc

9301 ggcctgtatg agacccgtat tgacctgagc cagctgggtg gtgatcccgg tagtggatcc

9361 gctgatccta agaaaaaacg caaagttggc ggcagcccta agaaaaaacg caaggtgggt

9421 ggcagtccta aaaagaagcg caaagtgggc ggcagcccga aaaagaaacg caaggtgcac

9481 caccatcacc atcattaagc

//

**7. pET24a_Cas9-BcStux2-NLS-His**

LOCUS Exported 9593 bp ds-DNA circular SYN 21-MAY-2019

DEFINITION synthetic circular DNA.

ACCESSION .

VERSION .

KEYWORDS 2 pET24a_Sp_Cas9_2xBcStuNLS_His

SOURCE synthetic DNA construct

ORGANISM synthetic DNA construct

REFERENCE 1 (bases 1 to 9593)

AUTHORS Thomas Leisen

TITLE -

JOURNAL -

FEATURES Location/Qualifiers

source 1..9593

/organism="synthetic DNA construct"

/lab_host="Escherichia coli"

/mol_type="other DNA"

CDS 14..31

/codon_start=1

/product="6xHis affinity tag"

/label=6xHis

terminator 98..145

/label=T7 terminator

/note="transcription terminator for bacteriophage T7 RNA

polymerase"

rep_origin 182..637

/label=f1 origin

/label=f1\origin

/note="/vntifkey=33"

CDS complement(730..1545)

/codon_start=1

/gene="aph(3')-Ia"

/product="aminoglycoside phosphotransferase"

/label=KanR

/note="confers resistance to kanamycin in bacteria or G418

(Geneticin(R)) in eukaryotes"

rep_origin 1667..2255

/label=ori

/note="high-copy-number ColE1/pMB1/pBR322/pUC origin of

replication"

CDS complement(3685..4767)

/codon_start=1

/gene="lacI"

/product="lac repressor"

/label=lacI

/note="The lac repressor binds to the lac operator to

inhibit transcription in E. coli. This inhibition can be

relieved by adding lactose or

isopropyl-beta-D-thiogalactopyranoside (IPTG)."

promoter complement(4768..4845)

/gene="lacI"

/label=lacI promoter

promoter 5154..5172

/label=T7 promoter

/note="promoter for bacteriophage T7 RNA polymerase"

CDS 5173..5197

/codon_start=1

/label=lac operator

/label=lac\operator

/note="/vntifkey=4"

/translation="GIVSG*QF"

RBS 5212..5234

/note="efficient ribosome binding site from bacteriophage

T7 gene 10 (Olins and Rangwala, 1989)"

CDS 5242..9345

/codon_start=1

/label=Sp_Cas9

/note="/vntifkey=4"

/translation="MDKKYSIGLDIGTNSVGWAVITDEYKVPSKKFKVLGNTDRHSIKK

NLIGALLFDSGETAEATRLKRTARRRYTRRKNRICYLQEIFSNEMAKVDDSFFHRLEES

FLVEEDKKHERHPIFGNIVDEVAYHEKYPTIYHLRKKLVDSTDKADLRLIYLALAHMIK

FRGHFLIEGDLNPDNSDVDKLFIQLVQTYNQLFEENPINASGVDAKAILSARLSKSRRL

ENLIAQLPGEKKNGLFGNLIALSLGLTPNFKSNFDLAEDAKLQLSKDTYDDDLDNLLAQ

IGDQYADLFLAAKNLSDAILLSDILRVNTEITKAPLSASMIKRYDEHHQDLTLLKALVR

QQLPEKYKEIFFDQSKNGYAGYIDGGASQEEFYKFIKPILEKMDGTEELLVKLNREDLL

RKQRTFDNGSIPHQIHLGELHAILRRQEDFYPFLKDNREKIEKILTFRIPYYVGPLARG

NSRFAWMTRKSEETITPWNFEEVVDKGASAQSFIERMTNFDKNLPNEKVLPKHSLLYEY

FTVYNELTKVKYVTEGMRKPAFLSGEQKKAIVDLLFKTNRKVTVKQLKEDYFKKIECFD

SVEISGVEDRFNASLGTYHDLLKIIKDKDFLDNEENEDILEDIVLTLTLFEDREMIEER

LKTYAHLFDDKVMKQLKRRRYTGWGRLSRKLINGIRDKQSGKTILDFLKSDGFANRNFM

QLIHDDSLTFKEDIQKAQVSGQGDSLHEHIANLAGSPAIKKGILQTVKVVDELVKVMGR

HKPENIVIEMARENQTTQKGQKNSRERMKRIEEGIKELGSQILKEHPVENTQLQNEKLY

LYYLQNGRDMYVDQELDINRLSDYDVDHIVPQSFLKDDSIDNKVLTRSDKNRGKSDNVP

SEEVVKKMKNYWRQLLNAKLITQRKFDNLTKAERGGLSELDKAGFIKRQLVETRQITKH

VAQILDSRMNTKYDENDKLIREVKVITLKSKLVSDFRKDFQFYKVREINNYHHAHDAYL

NAVVGTALIKKYPKLESEFVYGDYKVYDVRKMIAKSEQEIGKATAKYFFYSNIMNFFKT

EITLANGEIRKRPLIETNGETGEIVWDKGRDFATVRKVLSMPQVNIVKKTEVQTGGFSK

ESILPKRNSDKLIARKKDWDPKKYGGFDSPTVAYSVLVVAKVEKGKSKKLKSVKELLGI

TIMERSSFEKNPIDFLEAKGYKEVKKDLIIKLPKYSLFELENGRKRMLASAGELQKGNE

LALPSKYVNFLYLASHYEKLKGSPEDNEQKQLFVEQHKHYLDEIIEQISEFSKRVILAD

ANLDKVLSAYNKHRDKPIREQAENIIHLFTLTNLGAPAAFKYFDTTIDRKRYTSTKEVL

DATLIHQSITGLYETRIDLSQLGGD"

misc_feature 9364..9459

/label=BcStuNLS

/note="/vntifkey=21"

misc_feature 9475..9570

/label=BcStuNLS

/note="/vntifkey=21"

CDS 9571..9588

/codon_start=1

/product="6xHis affinity tag"

/label=6xHis

ORIGIN

1 ggccgcactc gagcaccacc accaccacca ctgagatccg gctgctaaca aagcccgaaa

61 ggaagctgag ttggctgctg ccaccgctga gcaataacta gcataacccc ttggggcctc

121 taaacgggtc ttgaggggtt ttttgctgaa aggaggaact atatccggat tggcgaatgg

181 gacgcgccct gtagcggcgc attaagcgcg gcgggtgtgg tggttacgcg cagcgtgacc

241 gctacacttg ccagcgccct agcgcccgct cctttcgctt tcttcccttc ctttctcgcc

301 acgttcgccg gctttccccg tcaagctcta aatcgggggc tccctttagg gttccgattt

361 agtgctttac ggcacctcga ccccaaaaaa cttgattagg gtgatggttc acgtagtggg

421 ccatcgccct gatagacggt ttttcgccct ttgacgttgg agtccacgtt ctttaatagt

481 ggactcttgt tccaaactgg aacaacactc aaccctatct cggtctattc ttttgattta

541 taagggattt tgccgatttc ggcctattgg ttaaaaaatg agctgattta acaaaaattt

601 aacgcgaatt ttaacaaaat attaacgttt acaatttcag gtggcacttt tcggggaaat

661 gtgcgcggaa cccctatttg tttatttttc taaatacatt caaatatgta tccgctcatg

721 aattaattct tagaaaaact catcgagcat caaatgaaac tgcaatttat tcatatcagg

781 attatcaata ccatattttt gaaaaagccg tttctgtaat gaaggagaaa actcaccgag

841 gcagttccat aggatggcaa gatcctggta tcggtctgcg attccgactc gtccaacatc

901 aatacaacct attaatttcc cctcgtcaaa aataaggtta tcaagtgaga aatcaccatg

961 agtgacgact gaatccggtg agaatggcaa aagtttatgc atttctttcc agacttgttc

1021 aacaggccag ccattacgct cgtcatcaaa atcactcgca tcaaccaaac cgttattcat

1081 tcgtgattgc gcctgagcga gacgaaatac gcgatcgctg ttaaaaggac aattacaaac

1141 aggaatcgaa tgcaaccggc gcaggaacac tgccagcgca tcaacaatat tttcacctga

1201 atcaggatat tcttctaata cctggaatgc tgttttcccg gggatcgcag tggtgagtaa

1261 ccatgcatca tcaggagtac ggataaaatg cttgatggtc ggaagaggca taaattccgt

1321 cagccagttt agtctgacca tctcatctgt aacatcattg gcaacgctac ctttgccatg

1381 tttcagaaac aactctggcg catcgggctt cccatacaat cgatagattg tcgcacctga

1441 ttgcccgaca ttatcgcgag cccatttata cccatataaa tcagcatcca tgttggaatt

1501 taatcgcggc ctagagcaag acgtttcccg ttgaatatgg ctcataacac cccttgtatt

1561 actgtttatg taagcagaca gttttattgt tcatgaccaa aatcccttaa cgtgagtttt

1621 cgttccactg agcgtcagac cccgtagaaa agatcaaagg atcttcttga gatccttttt

1681 ttctgcgcgt aatctgctgc ttgcaaacaa aaaaaccacc gctaccagcg gtggtttgtt

1741 tgccggatca agagctacca actctttttc cgaaggtaac tggcttcagc agagcgcaga

1801 taccaaatac tgtccttcta gtgtagccgt agttaggcca ccacttcaag aactctgtag

1861 caccgcctac atacctcgct ctgctaatcc tgttaccagt ggctgctgcc agtggcgata

1921 agtcgtgtct taccgggttg gactcaagac gatagttacc ggataaggcg cagcggtcgg

1981 gctgaacggg gggttcgtgc acacagccca gcttggagcg aacgacctac accgaactga

2041 gatacctaca gcgtgagcta tgagaaagcg ccacgcttcc cgaagggaga aaggcggaca

2101 ggtatccggt aagcggcagg gtcggaacag gagagcgcac gagggagctt ccagggggaa

2161 acgcctggta tctttatagt cctgtcgggt ttcgccacct ctgacttgag cgtcgatttt

2221 tgtgatgctc gtcagggggg cggagcctat ggaaaaacgc cagcaacgcg gcctttttac

2281 ggttcctggc cttttgctgg ccttttgctc acatgttctt tcctgcgtta tcccctgatt

2341 ctgtggataa ccgtattacc gcctttgagt gagctgatac cgctcgccgc agccgaacga

2401 ccgagcgcag cgagtcagtg agcgaggaag cggaagagcg cctgatgcgg tattttctcc

2461 ttacgcatct gtgcggtatt tcacaccgca tatatggtgc actctcagta caatctgctc

2521 tgatgccgca tagttaagcc agtatacact ccgctatcgc tacgtgactg ggtcatggct

2581 gcgccccgac acccgccaac acccgctgac gcgccctgac gggcttgtct gctcccggca

2641 tccgcttaca gacaagctgt gaccgtctcc gggagctgca tgtgtcagag gttttcaccg

2701 tcatcaccga aacgcgcgag gcagctgcgg taaagctcat cagcgtggtc gtgaagcgat

2761 tcacagatgt ctgcctgttc atccgcgtcc agctcgttga gtttctccag aagcgttaat

2821 gtctggcttc tgataaagcg ggccatgtta agggcggttt tttcctgttt ggtcactgat

2881 gcctccgtgt aagggggatt tctgttcatg ggggtaatga taccgatgaa acgagagagg

2941 atgctcacga tacgggttac tgatgatgaa catgcccggt tactggaacg ttgtgagggt

3001 aaacaactgg cggtatggat gcggcgggac cagagaaaaa tcactcaggg tcaatgccag

3061 cgcttcgtta atacagatgt aggtgttcca cagggtagcc agcagcatcc tgcgatgcag

3121 atccggaaca taatggtgca gggcgctgac ttccgcgttt ccagacttta cgaaacacgg

3181 aaaccgaaga ccattcatgt tgttgctcag gtcgcagacg ttttgcagca gcagtcgctt

3241 cacgttcgct cgcgtatcgg tgattcattc tgctaaccag taaggcaacc ccgccagcct

3301 agccgggtcc tcaacgacag gagcacgatc atgcgcaccc gtggggccgc catgccggcg

3361 ataatggcct gcttctcgcc gaaacgtttg gtggcgggac cagtgacgaa ggcttgagcg

3421 agggcgtgca agattccgaa taccgcaagc gacaggccga tcatcgtcgc gctccagcga

3481 aagcggtcct cgccgaaaat gacccagagc gctgccggca cctgtcctac gagttgcatg

3541 ataaagaaga cagtcataag tgcggcgacg atagtcatgc cccgcgccca ccggaaggag

3601 ctgactgggt tgaaggctct caagggcatc ggtcgagatc ccggtgccta atgagtgagc

3661 taacttacat taattgcgtt gcgctcactg cccgctttcc agtcgggaaa cctgtcgtgc

3721 cagctgcatt aatgaatcgg ccaacgcgcg gggagaggcg gtttgcgtat tgggcgccag

3781 ggtggttttt cttttcacca gtgagacggg caacagctga ttgcccttca ccgcctggcc

3841 ctgagagagt tgcagcaagc ggtccacgct ggtttgcccc agcaggcgaa aatcctgttt

3901 gatggtggtt aacggcggga tataacatga gctgtcttcg gtatcgtcgt atcccactac

3961 cgagatatcc gcaccaacgc gcagcccgga ctcggtaatg gcgcgcattg cgcccagcgc

4021 catctgatcg ttggcaacca gcatcgcagt gggaacgatg ccctcattca gcatttgcat

4081 ggtttgttga aaaccggaca tggcactcca gtcgccttcc cgttccgcta tcggctgaat

4141 ttgattgcga gtgagatatt tatgccagcc agccagacgc agacgcgccg agacagaact

4201 taatgggccc gctaacagcg cgatttgctg gtgacccaat gcgaccagat gctccacgcc

4261 cagtcgcgta ccgtcttcat gggagaaaat aatactgttg atgggtgtct ggtcagagac

4321 atcaagaaat aacgccggaa cattagtgca ggcagcttcc acagcaatgg catcctggtc

4381 atccagcgga tagttaatga tcagcccact gacgcgttgc gcgagaagat tgtgcaccgc

4441 cgctttacag gcttcgacgc cgcttcgttc taccatcgac accaccacgc tggcacccag

4501 ttgatcggcg cgagatttaa tcgccgcgac aatttgcgac ggcgcgtgca gggccagact

4561 ggaggtggca acgccaatca gcaacgactg tttgcccgcc agttgttgtg ccacgcggtt

4621 gggaatgtaa ttcagctccg ccatcgccgc ttccactttt tcccgcgttt tcgcagaaac

4681 gtggctggcc tggttcacca cgcgggaaac ggtctgataa gagacaccgg catactctgc

4741 gacatcgtat aacgttactg gtttcacatt caccaccctg aattgactct cttccgggcg

4801 ctatcatgcc ataccgcgaa aggttttgcg ccattcgatg gtgtccggga tctcgacgct

4861 ctcccttatg cgactcctgc attaggaagc agcccagtag taggttgagg ccgttgagca

4921 ccgccgccgc aaggaatggt gcatgcaagg agatggcgcc caacagtccc ccggccacgg

4981 ggcctgccac catacccacg ccgaaacaag cgctcatgag cccgaagtgg cgagcccgat

5041 cttccccatc ggtgatgtcg gcgatatagg cgccagcaac cgcacctgtg gcgccggtga

5101 tgccggccac gatgcgtccg gcgtagagga tcgagatctc gatcccgcga aattaatacg

5161 actcactata ggggaattgt gagcggataa caattcccct ctagaaataa ttttgtttaa

5221 ctttaagaag gagatataca tatggataag aaatatagca ttggcctgga cattggcacc

5281 aacagcgttg gttgggcagt gattaccgac gagtacaagg ttccgagcaa gaagttcaag

5341 gtgctgggta acaccgatcg ccatagtatc aagaaaaact taatcggcgc cctgctgttt

5401 gatagtggcg agaccgcaga agcaacacgc ctgaaacgca cagcacgccg tcgttataca

5461 cgtcgtaaga atcgcatttg ctatctgcag gaaattttta gtaatgagat ggccaaggtg

5521 gatgatagct tctttcaccg tctggaggag agcttcctgg tggaagagga caagaaacac

5581 gagcgccatc cgatctttgg caatatcgtg gacgaagtgg cctaccatga gaagtatccg

5641 acaatctacc atctgcgcaa gaagctggtt gacagcaccg ataaggccga cctgcgtctg

5701 atctatttag ccctggcaca catgatcaaa tttcgcggcc atttcctgat cgagggtgat

5761 ctgaatccgg acaacagtga cgttgacaag ttatttattc agctggttca aacatacaat

5821 caactgttcg aagaaaatcc gattaatgcc agcggcgtgg atgcaaaggc aatcctgagt

5881 gcccgcctga gcaaaagccg tcgtctggaa aatctgatcg cacagctgcc gggtgagaag

5941 aagaacggcc tgtttggtaa cttaattgca ctgagcctgg gtttaacccc gaactttaaa

6001 agcaacttcg acctggcaga agacgccaag ttacagctga gtaaagacac ctacgacgac

6061 gatctggaca acttattagc ccagatcggt gaccagtatg ccgatctgtt tctggcagca

6121 aagaacctga gcgatgcaat cctgctgagc gacattctgc gtgtgaatac cgagattacc

6181 aaagccccgt taagcgcaag tatgattaag cgctatgatg agcaccacca ggacttaacc

6241 ctgctgaaag ccctggtgcg ccagcagtta cctgagaaat acaaagaaat cttttttgat

6301 caaagtaaga atggctacgc cggttatatc gatggcggtg ccagccaaga ggagttttat

6361 aaattcatca aacctatcct ggagaaaatg gacggcaccg aagaactgct ggttaaatta

6421 aatcgcgaag atttactgcg caagcagcgt accttcgata atggcagcat tccgcaccaa

6481 atccacctgg gcgaattaca tgcaatcctg cgccgtcagg aagattttta cccgtttctg

6541 aaagataatc gtgaaaaaat cgaaaagatc ctgaccttcc gcattccgta ttatgttggt

6601 cctctggccc gtggtaacag tcgctttgca tggatgaccc gcaagagcga ggaaacaatt

6661 accccgtgga atttcgaaga agttgttgac aaaggtgcca gcgcccagag cttcatcgag

6721 cgcatgacca actttgataa gaacctgccg aatgaaaaag ttctgccgaa acatagtctg

6781 ctgtatgagt atttcaccgt gtacaatgag ttaacaaaag ttaaatatgt gacagaaggt

6841 atgcgcaagc cggcctttct gagcggtgag cagaagaagg ccatcgtgga cttactgttt

6901 aagaccaatc gcaaagttac cgttaaacag ctgaaagaag actatttcaa gaaaattgag

6961 tgtttcgata gcgttgaaat tagtggcgtg gaggatcgct tcaacgcaag tctgggcacc

7021 tatcatgatt tactgaagat tattaaggac aaggacttcc tggacaacga agagaacgag

7081 gacattttag aggacatcgt gctgaccctg accctgtttg aagatcgcga gatgattgag

7141 gagcgcctga aaacctacgc acacctgttc gatgacaagg tgatgaagca gctgaaacgt

7201 cgccgctata ccggctgggg tcgcttaagc cgcaagctga tcaacggcat ccgcgacaag

7261 cagagcggca aaaccatcct ggacttcctg aagagcgatg gttttgccaa ccgcaatttc

7321 atgcagctga tccacgacga cagcctgacc tttaaagaag acattcagaa agcacaggtt

7381 agtggtcaag gcgacagcct gcatgaacac atcgcaaacc tggcaggtag cccggccatt

7441 aagaaaggta ttctgcagac cgtgaaggtg gtggatgagc tggttaaggt gatgggccgt

7501 cacaaaccgg aaaatattgt tatcgaaatg gcccgtgaga atcaaacaac ccagaagggc

7561 cagaaaaata gccgcgagcg catgaagcgc attgaggaag gcattaaaga attaggcagc

7621 caaattctga aggagcatcc ggttgaaaac acacagctgc agaatgagaa gctgtacctg

7681 tactacctgc agaatggccg cgacatgtat gttgatcagg aattagatat taatcgtctg

7741 agcgactacg acgttgacca tatcgtgcct cagagctttc tgaaggatga cagcatcgac

7801 aacaaggtgc tgacccgtag cgataagaac cgcggtaaga gcgataacgt tccgagcgaa

7861 gaagttgtta agaaaatgaa aaactattgg cgccagctgt taaacgccaa gttaatcacc

7921 caacgtaagt tcgataatct gacaaaggca gaacgtggtg gcctgagcga gctggacaaa

7981 gccggtttca ttaaacgcca gctggtggag acacgccaga tcaccaaaca cgtggcccaa

8041 atcctggaca gccgtatgaa tacaaagtat gatgaaaatg ataaattaat tcgtgaagtt

8101 aaagttatta ccctgaagag caagttagtg agcgacttcc gtaaggactt ccagttttac

8161 aaagttcgcg aaattaataa ttaccaccat gcacacgatg catacctgaa tgccgttgtt

8221 ggcaccgccc tgattaagaa gtacccgaag ctggagagcg agttcgttta tggtgattac

8281 aaagtgtacg atgtgcgcaa aatgatcgca aagagcgaac aggagattgg caaagccacc

8341 gccaaatact tcttttatag caatattatg aatttcttca aaaccgaaat taccctggcc

8401 aatggcgaga ttcgtaaacg tccgctgatc gaaacaaatg gcgaaacagg cgagatcgtg

8461 tgggataagg gccgtgactt tgcaaccgtt cgcaaggtgc tgagcatgcc gcaggtgaat

8521 attgttaaga aaactgaagt tcaaacaggt ggctttagta aggagagcat tctgcctaaa

8581 cgcaacagcg acaaactgat cgcacgcaag aaagactggg accctaagaa atacggcggc

8641 tttgacagcc cgaccgttgc ctatagtgtg ctggtggttg ccaaggtgga gaagggtaaa

8701 agtaaaaaat taaagagtgt taaagagctg ttaggcatta caatcatgga gcgcagcagt

8761 tttgaaaaga accctatcga cttcctggaa gccaagggtt ataaagaagt taaaaaggac

8821 ctgattatta agctgcctaa atatagctta tttgagttag agaacggccg taaacgcatg

8881 ctggcaagcg ccggtgaact gcaaaagggc aatgagctgg ccctgccgag taagtatgtg

8941 aatttcctgt atttagcaag tcactatgaa aagttaaagg gcagcccgga ggacaacgag

9001 cagaagcagc tgtttgttga acagcacaaa cactacctgg acgagattat cgaacagatc

9061 agtgagttca gtaagcgtgt gatcttagcc gacgcaaacc tggacaaagt gctgagcgca

9121 tataacaagc accgtgacaa accgattcgc gagcaggccg agaacattat tcatttattt

9181 accttaacca atctgggtgc accggcagcc ttcaaatatt ttgacacaac catcgaccgc

9241 aagcgctaca caagcacaaa agaagttctg gacgcaacac tgatccacca gagcatcacc

9301 ggcctgtatg agacccgtat tgacctgagc cagctgggtg gtgatcccgg tagtggatcc

9361 gctggcaata aacgcggccg tgatgatgaa gatgatggtc gtccgagcag tcgtggcccc

9421 ggtatggccg atgccgatgg tttaaaacgc cgcaaaactg gtagcagcgg tgctggtaac

9481 aaacgcggtc gcgatgatga ggatgatggc cgtccgagta gtcgcggtcc gggtatggct

9541 gatgccgacg gtctgaaacg tcgcaaaacc caccaccatc atcaccatta agc

//
