# Supplementary material for: CRISPR/Cas with ribonucleoprotein complexes and transiently selected telomere vectors allows highly efficient marker-free and multiple genome editing in Botrytis cinerea
Source: PLoS Pathog. 2020 Aug 17;16(8):e1008326. doi: 10.1371/journal.ppat.1008326 (PMC7451986; doi:10.1371/journal.ppat.1008326)
Supplement: Attachment — Submitted filename: Response to reviewers.docx [file ppat.1008326.s022.docx]

**Decision mail from PLoS Pathogens (23 Feb 2020) and responses to reviewers (in red)**

Dear Dr. Hahn,

Thank you very much for submitting your manuscript "CRISPR/Cas with ribonucleoprotein complexes and transiently selected telomere vectors allows highly efficient marker-free and multiple genome editing in Botrytis cinerea" for consideration at PLOS Pathogens. As with all papers reviewed by the journal, your manuscript was reviewed by members of the editorial board and by several independent reviewers. In light of the reviews (below this email), we would like to invite the resubmission of a significantly-revised version that takes into account the reviewers' comments. We would like to note that particularly the concerns of referee 2 deserve careful attention and require additional evidence to support the claims that are made. We cannot make any decision about publication until we have seen the revised manuscript and your response to the reviewers' comments. Your revised manuscript will also be sent to reviewers for further evaluation.

When you are ready to resubmit, please upload the following:

[1] A letter containing a detailed list of your responses to the review comments and a description of the changes you have made in the manuscript. Please note while forming your response, if your article is accepted, you may have the opportunity to make the peer review history publicly available. The record will include editor decision letters (with reviews) and your responses to reviewer comments. If eligible, we will contact you to opt in or out.

[2] Two versions of the revised manuscript: one with either highlights or tracked changes denoting where the text has been changed; the other a clean version (uploaded as the manuscript file).

Important additional instructions are given below your reviewer comments.

Please prepare and submit your revised manuscript within 60 days. If you anticipate any delay, please let us know the expected resubmission date by replying to this email. Please note that revised manuscripts received after the 60-day due date may require evaluation and peer review similar to newly submitted manuscripts.

Thank you again for your submission. We hope that our editorial process has been constructive so far, and we welcome your feedback at any time. Please don't hesitate to contact us if you have any questions or comments.

Sincerely,

Melvin Bolton, Guest Editor
PLOS Pathogens

Bart Thomma, Section Editor
PLOS Pathogens

Kasturi Haldar, Editor-in-Chief
PLOS Pathogens
orcid.org/0000-0001-5065-158X

Michael Malim, Editor-in-Chief
PLOS Pathogens
orcid.org/0000-0002-7699-2064
***********************

Reviewer's Responses to Questions

**Part I - Summary**
Please use this section to discuss strengths/weaknesses of study, novelty/significance, general execution and scholarship.


**Reviewer #1**

Leisen et al. present a novel adaptation of CRISPR/Cas9 technology to the necrotrophic plant pathogen Botrytis cinerea. The major advancement presented in this manuscript is the utilization of a stable telomere vector to enable transient selection of CRISPR/Cas9 mediated edits without integration of the selectable marker. This protocol was expanded to the important plant pathogen Magnaporthe oryzae and was shown to function with high efficiency. This methodology was also used to test various types of genome edits (knock-in of a fluorescent protein, gene deletions), but also used to assess the effects of all possible amino acid substitutions in codon 272 of the sdhB gene. Phenotypic effects of each mutation were evaluated and novel insights into the SDHI resistance mechanisms were obtained. Overall, the experiments presented in this manuscript were well designed and controlled, and the data supports the authors’ conclusions. The paper was well-written and generally clear. The methodology developed in this manuscript is a significant achievement for functional gene analyses across many fungal taxa. Additionally, although not the main focus of the paper, the sdhB functional data presented not only highlights the power of this method, but also provides important information regarding fungicide resistance and may have implications in fungicide chemistry development and/or testing.

**Reviewer #2: Review PPATHOGENS-D-20-00059**
The manuscript presents a potentially interesting new transient selection system that might improve the efficiency of the CrispR-based co-editing strategy previously reported in the rice blast fungus. The authors claim that their system uses ‘autonomous unstable telomere vectors’ to achieve this increase in efficiency. However there is no experimental evidence to support this claim except for the loss of resistance due to culturing the fungal strains without selection. In a paper that describes a novel experimental approach, it is important that all necessary experimental details are given – for example how long does this curing process take and how exactly is it done (does it require single spore isolation, for instance)?

A1: We have provided additional data which demonstrate the instability of pTEL under non-selective conditions based on growth tests (new S1 Table), and its complete loss from the majority of transformants based on PCR data and genome sequencing (new 7S Fig). We have also provided additional experimental details in the text and in the Methods part.

The authors additionally claim that their approach allows ‘introduction of ‘clean’ markerless changes into the genome without unwanted modifications’ and in the discussion they claim the generation of ‘edited strains without any other genomic alterations’ – again there is no experimental evidence to support such a claim and this could not be made without whole genome sequence data to support the assertion of no ‘unwanted modifications’. It is also very important that the authors show that their approach is not more mutagenic than non-CrispR methods.

A2: There have been already two publications (Al Abdallah et al., 2018; Foster et al., 2019) which indicated that there is no evidence for Cas9-RNPs introducing off-target mutations. They report about a background of mutations in transformed strains of ca. 350 additional mutations in A. fumigatus and 74-77 mutations in *M. oryzae*. We additionally performed whole genome sequencing of *B. cinerea* transformants resulting from pTEL-mediated RNP editing and as controls pTEL-only transformants and transformants obtained by regular k.o. mutagenesis. These data showed that off-target mutation frequencies in *B. cinerea* are extremely low in all transformants.

The experiments made with Botrytis seem to have in the main, well-performed, except that in most of the cases reported the genotype is inferred from the phenotype only. There was an attempt made to prove that the strains assumed to be inactivated for BOS1 really contain indels in the target gene. However there is no genotype analysis made for the bot2- and boa6- transformants, as far as this reviewer could see – it is really necessary to sequence the amplicons in these cases. This is very important.

A3: We have performed a genotype analysis of the bot2 and bot2boa6 transformants (Fig. S6B), which shows that the right flanks of the selection markers have been integrated in loco into bot2 and boa6. We now provide genome sequencing data for a bot2boa6 transformant which demonstrate that integration of the two resistance cassettes has occurred by homologous recombination as expected*.*

There are more serious issues with the Magnaporthe transformations which appear to be just reporting one transformation experiment which was not reproduced. The authors must know that an experiment is not reproduced unless repeated exactly, and that performing an experiment with three different concentrations of a constituent does not constitute repetition of the experiment. There is again very limited attempt to genotype the mutants generated - just three strains were analysed (the PCR products were sequenced) according to the manuscript and this information is not actually shown at all. I feel that it is necessary to confirm the assumed genotypes in many more than just three strains. The transformation plate shown in Fig. 6 seems to show confluent growth and I wonder how the authors were able to isolate individual clones from such a dense plating in the absence of an obvious phenotype such as altered pigmentation.

A4: We apologize that in the initial version of our manuscript it was not sufficiently explained that results of these experiment are from independent biological replicates. We now have repeated the cotransformation experiment for targeting MoPIT via HR three more times, and the analysis of the transformants has now been done more thoroughly by sequencing of the insertion site. These data confirm our conclusions from the first experiment. As shown in the extended Fig. 6 and in the newly made S11 Fig, more transformants have been analysed for correct integration of the MoPIT k.o. construct by PCR analysis and sequencing. We have included a map of MoPIT in S11 Fig. to facilitate better understanding of the experimental design.

In summary I believe that this work shows a lack of rigour in many places and makes very broad claims based on very little evidence and I do not think it therefore merits publication in this journal. The method described is in actual fact, very largely an adaptation of existing approaches that have now been reported in many species, including notably Magnaporthe oryzae, where a very similar approach was published in 2018.

I would recommend that the authors address the fate of the ‘telomere-vectors’ used using pulsed field gel electrophoresis and Southern blotting and/or whole genome sequencing before and after curing in both species. This would provide the evidence they really need to support the claims being made. It is also important to explore what happens to the plasmids and whether they are really maintained extra-chromosomally, as assumed by the authors. Additionally, given the fact that a long period of growth with and then without selection is required, I would again recommend that the authors perform whole genome sequencing of some of the strains generated to test whether their approach is more mutagenic than non-CrispR based gene targeting methods.

A5: We are now presenting detailed data about the instability of pTEL vectors in *B. cinerea* (new S1 Table), its linearization to an acentric minichromosome with two telomers (lacking the KanR gene present in the circular pTEL plasmids that are propagated in E. coli), and its complete loss in several edited strains by genome sequencing (new S7 Fig.).

The authors should also describe the past Magnaporthe CrispR approaches, more thoroughly in their introduction – it should be made clear that efficient CrispR-based gene targeting approaches have already been reported in the species and that a co-editing approach, as described here, has already been described before. Likewise in fairness to the scientists that developed the telomere targeting vector previously, the literature describing it should be introduced in the introduction. The authors seem to be rather over-claiming their approach, which is at best an incremental advance to other reported methods, using telomere vectors originally generated by others.

A6: We have already acknowledged the excellent work of Foster et al. for the development of RNP-CRISPR/Cas in *M. oryzae* in our original manuscript. We have now added in the introduction their co-editing approach using benomyl/diethofencarb counterselection (which however has not been tested in combination with Cas9-RNP in their work), with the following sentence ‚In *M. oryzae*, a novel selection strategy was devised based on switching between two antagonistic states of resistance (to either of the fungicides benomyl and diethofencarb), depending on the sequence of codon 198 of the TUB2 gene, which allows the construction of markerless mutations.’

Furthermore, to avoid over-claiming our approach, as critized by the reviewer, we have modified the text at several positions.

The manuscript might be better approached not as a ‘methods paper’, unless more detailed evidence in support of the claims made is provided, but instead by focusing on the application in understanding fungicide resistance development, which is arguably the most novel finding that is reported.

**Reviewer #3**

The submitted manuscript describes the development and testing of RNP-Cas9 based genome editing in the model fungal plant pathogens Botrytis cinerea and Magnaporthe oryzae. The research describes the use of a transient selection vector, which contains teleomeres for temporary selection without marker integration. Results using this method showed efficient genome editing for a fungicide resistance target gene, allowing the creation of 20 amino acid coding mutants for a particular residue known to contribute to fungicide resistance. The manuscript provides details and describes an approach that will further advance the use of CRISPR-based genome editing in fungi and will likely be of interest to those in the field. It is a nice study.

There are however sections of the manuscript that are not well described or presented that make it difficult to read and understand. These should be addressed before acceptance as a key attribute of this work is to facilitate other labs to replicate the work and system for enhanced genome editing efficiency.

A7: We have extended the text at several passages to improve readibility and clarity.

**Part II – Major Issues: Key Experiments Required for Acceptance**
Please use this section to detail the key new experiments or modifications of existing experiments that should be absolutely required to validate study conclusions.

Generally, there should be no more than 3 such required experiments or major modifications for a "Major Revision" recommendation. If more than 3 experiments are necessary to validate the study conclusions, then you are encouraged to recommend "Reject".

Reviewer #1: (No Response)

Reviewer #2: More specific comments
Fig 2 B – it is necessary to explain exactly what T1 T2 T3 and T4 refer to – this is not clear

A8: T1, T2, T3 and T4 correspond to the different sgRNAs used for the transformations shown in Fig. 2A, where we corrected the labeling of sgRNA Bos1-T2; this is now explained better in the legend to Fig. 2.
Fig 5 F and G – the sizes of the proteins do not correspond between the two panels?

A9: We have reorganized Fig. 5, by moving the transformation results (formerly Fig. 5C) into the S8 Fig, and extending the figure plates with GFP-stained strains (Fig. 5Dnew) and a Western blot stained for GFP, now including a Bcgfp1 strain as a control (Fig. 5Enew). The bands on the blot are according to the predicted monomeric sizes of Sod1-GFP and GFP. However, they are not in Fig. 5F, which a shows a non-denaturing gel used for activity staining; Sod1 probably occuring as a dimer of 32 kDa (Chang et al., 1991); the migration behaviour of Sod1-GFP is difficult to explain. See also answers A19-A20.
Fig 6 – why are the pigmented transformants green – my understanding was that this fungus is grey (hence its former name)?

A10: Fig. 6A has been replaced by a picture showing more natural colors of the *M. oryzae* mycelium.

Fig S5 – how many times was this experiment repeated (if at all?) – this needs to be explicitly stated for all the experiments made in fact.

A11: Each of these experiments were performed three times; this is now indicated in the legend.
Fig S8 – there is really no need to show again CrispR based gene targeting with selection given that 3 publications have already demonstrated that this approach works very well in this species. It would additionally appear that this experiment was only carried out once.

A12: We agree, and have deleted Fig. S8; the targeting efficiency is now mentioned in the text.
Fig S9 A – these experiments were also only done once?

A13: We have repeated this experiment three more times and improved the documentation, see answer A4.

Reviewer #3: Conclusion lines 203-205. Can the authors summarize/quantify the types of edits observed to support the comments of ‘remarkably uniform mutation patterns.’ The description suggests that many different types of edits were recovered.

A14: As shown in Fig. 2, a majority of ‚+T‘ edits was obtained for the three sgRNAs which had a ‚T‘ in position 4 from the 3‘-end, which is located at the 5‘-side of the Cas9 cleavage site. To explain this better, we replaced the ambiguous term‚remarkably uniform mutation patterns‘ by the following phrase: ‚… our results show diverse NHEJ error-prone repair events as expected, but that with selected three sgRNAs containing ‚T‘ at position 4 counted from the 3’-terminus, the same ‘+T’ mutation was obtained in 68.3 ± 6.9% of the tested transformants.

Fig. 1: In 1E, what are the # of transformants relative to the N=4, 4, and 11. Also, it is not clear what the statistical test is comparing? Multiple comparison to what? In E, Tukeys post-hoc is reported, while in F Dunnets testing used? Why the difference? For Dunnetts, what is compared against?

A15: We are not sure about the 1st question: ‚n=4,4 and 11‘ indicate the number of transformation experiments for each of the three strategies. Tukey and Dunnett are both multiple comparisons post hoc tests after ANOVA, but Tukey compares each of the three values against each other, whereas Dunnet compares all values with the control (here: SV40-Cas9-SV40). To make the use of Dunnet in Fig. 1F clear, we have extended the text in the results, which now reads: ‚We then compared recombinant Cas9 protein variants carrying different NLS for their efficiency in RNP-mediated transformation. Compared to a commercially available Cas9 containing SV40-NLS on both termini, Cas9 with four C-terminal tandem copies of SV40-NLS conferred slightly higher, and Cas9 with two C- Stu-NLS significantly higher *in vivo* editing activities (Fig. 1F). Cas9-Stux2-NLS was therefore used in all subsequent experiments with *B. cinerea*.‘

Fig. 2: The labeling is unclear between A and B. Which bars in B correspond to guides in A. It should be more explicit. See answer A8 to reviewer 2.

S5 fig. (lines 208-215)
For Fen insertion with 1kb flank, it does not appear as though there was a control. There is no information provided on the rate of Fen insertion from linear or plasmid without Cas9. With such long homologous flanks (1kb) there could be a significant number of insertions from homologous recombination regardless of Cas9 cutting. This data is not interpretable without controls.

A16: The requested control data are availabe and now included in the legend of S5 fig (transformants obtained -without Cas9- with pBS_bos-KO_Fen circular: 0,0,0; with PCR fragm.: 0,0,1.). As a note, transformation efficiencies were quite low in these experiments, we later modified the transformation protocol and obtained much higher transformation rates.

Fig. 3 The grey versus black is very difficult to see and the overall layout is difficult. There needs to be clearer labeling. It was not clear from the narrative why there is such a difference between Ipr and Fen resistance numbers as seen in Fig 3D.

A17: The layout of Fig. 3A-C has been changed. In Fig. 3D, the reason for the difference between Ipr and Fen resistance numbers is now discussed in the text (both NHEJ- and HR-derived transformants are IprR, wehreas only HR-derived transformants are FenR).

Throughout the text and Fig. 5, the term co-editing is used, however, it is co-transformation that is described. The expression of Fen from the pTel along with Cas9 editing is not co-editing. Only a single edit is made.

A18: We agree with the referee that ‚pTEL-mediated coediting‘ is not an adequate term. We have therefore replaced ‚coediting‘ in this context by ‚cotransformation‘ and ‚editing‘ throughout the text.

There is no control for 5E, 5F, 5G. What does a random GFP insertion look like? What does GFP expressed alone show on the blots? Is the blot in F and G from the image shown in E?

A19: We did not obtain random GFP insertions, all transformants analysed showed the similar punctate fluorescence which is distinct from cytoplasmic fluorescence. In 5Dnew, we have added a second insert showing a germling expressing cytoplasmic GFP. For 5Enew, we have made a new Western blot, this time including a second Sod1-GFP strain and a strain expressing GFP, see Fig. 5Enew. Yes, Figs. 5D, 5E and 5F are indeed from the same strain (#4).

What is the expected size of the Sod1-GFP fusion. Also, the analysis for SOD activity is not common knowledge and results should be explicitly explained for 5F

A20: We have considerably extended the text and the legend of Fig. 5 explaining the experiments with the Sod1-GFP strain, which now includes also a short discussion of the predicted and the apparent sizes of Sod1, Sod1-GFP and GFP on the activity-stained gel and the Western blot.

See also answer A9.

Line 313 again does not show co-editing. Only one locus is being edited, the co- is DNA uptake.

A21: This is true, rephrased to ‚cotransformation‘

Line 313-314, no data shown or referenced. (‚By contrast, the rate of cotransformation in Guy11ku80 was much lower.‘)

A22: Because the conclusion was based on as single experiment, we have deleted the sentence.

Is the M. oryzae data with the pTEL-Fen vector provided? Is line 314-315 for the pTEL-lines?

A23: Yes, the text refers to the pTEL lines.

S8 Fig has an error for the numbers. 18/18 is not 72%. Also, there are again no controls. No Cas9 alone, sgGuides alone. Also, the ku80 strain is to test for NHEJ versus HR, but there is not an hph insert lacking homology. That is needed to show that the ku80 strain fails to produce any transformants. This lack of a control becomes apparent for the results in fig 9.

A24: S8 Fig has been deleted, see answer A12.

The lack of explanation and labeling in S9 makes it very difficult to interpret. What is k.o.s? What are the asterisk for gel image? The reader should not be required to explicate these data/figures. It should be clearly labeled and described. Why are bands in M18 and M21 not mentioned?

A25: This experiment has been repeated three more times, and S9 figure remade to improve clarity, see answer A4.

How do they know that editing (small INDEL) did not take place in the PCR+ mutants? One interpretation of the KU80ko losing all integration of Hyg is that the integrations of the hyg were not HR but NHEJ insertions. There is no control where the hyg has no homology, so this cannot be ruled out. Also, why is this called coediting (line 320). Only one site is being edited, MoPIT.

A26: We have now performed a thorough analysis of the edited strains, including sequencing of the borders of MoPIT repair template insertion site, which clearly proved that seamless homologous recombination has occured in many cases.

Line 411-412. Why is highly efficient HR a requirement? Ku80 data shown in S9 suggests NHEJ is important for DNA integration.

A27: This is correct, we have changed the phrase to ‚highly efficient HR and/or NHEJ‘.

Line 419. It is not clear from data show in fig 6 that the transformants are from NHEJ? The hph donor is not described in the text with the data?

A28: Yes, Fig. 6A/B clear shows NHEJ-induced (white) transformants! Some of them have been sequenced, which is shown in the new A10 Fig. However, we made a mistake in the last column of S3 Table (former S1 Table), erroneously reading ‚white HygR colonies‘, which has been corrected to ‚white FenR colonies‘.

M&M. 516-517, It is not clear what this line is referring too? The functionality of the nucleases was tested? This data should be provided then.

A29: We have added a sentence saying that the functionality of the nucleases has been tested, and added to S3 Fig. a picture (C), showing *in vitro* cleavage of targets with Cas9-sgRNA-RNPs.

**Part III – Minor Issues: Editorial and Data Presentation Modifications**
Please use this section for editorial suggestions as well as relatively minor modifications of existing data that would enhance clarity.


Reviewer #1: P5L108: change “telomer” to “telomere”
P8l174: change “cinereal” to “cinerea”
Figure 1: change “Tuckey’s” to “Tukey’s” in the caption
S1 Figure: change “cinereal” to “cinerea”

A: All corrected.
P9L191-193: edit sentence

A: The sentence was corrected.
Figure 2: It appears that sgRNA Bos1-T2 is missing from part A of the figure (Bos1-T1 is labeled twice). A: We have corrected Fig. 2, see answer A8.
P10L222: Shouldn’t this be 64% (16/25 transformants without microhomology were both resisant to Fen and Ipr)?

A: Corrected
P10L223-224: What were the sequence artefacts introduced during integration of Fen via NHEJ (i.e. was there a prominent insertion/deletion type)? (‚Sequencing confirmed that the cassette had integrated via NHEJ into the cleavage site in Bos1‘)

A: We have now sequenced the borders of three FenR IprR transformants, and changed the text accordingly, which now reads: ‚Sequencing revealed in two cases perfect insertions of the FenR cassette, in the same orientation as with the Bos1 borders, and in one case an insertion accompanied by deletion of Bos1 DNA, which was not analysed further.‘
P12L273-279: What were the rates of confirmed Fen/Ipr double resistant transformants for each of the pTEL-Fen concentrations used in this experiment? The number of transformants appeared to increase with increasing pTEL-Fen concentration, but did the rate of co-editing increase? If these data were recorded, it would be nice to include them here.

A: We recorded the numbers in some of the experiments but did not see a trend for changes in co-editing frequencies with increasing pTEL-Fen concentration. We therefore didn’t include them in the results.
P14L333/Figure7: An explanation of why two sgRNAs were used in this experiment would be useful for the audience.

A: This is now explained in the text: ‚Two different gRNAs were used to lower the risk of choosing one with low editing efficiency.‘
P18L416: Can the author’s provide any speculation as to why nep1/nep2 double knockouts were not obtained?

A: To address this point, we have added the following text: Given the high efficiency of CRISPR/Cas-mediated targeting, the frequency of double editing was lower than expected. In the bot2 boa6 double editing experiments, only three out of 20 double resistant transformants were confirmed to contain the desired mutations (S6 Fig), and in the pTEL-mediated double editing experiment with nep1 and nep2, no double edits were identified. These data indicate that there might be an increased rate of ectopic integrations of RT due to as yet uncharacterized interference effects if multiple RNPs and RTs are transformed. In these experiments, the transformation rates (bot2 boa6) and the editing efficiencies (nep1 nep2) were lower than in most other experiments. High transformation rates and editing efficiencies, and careful analysis of the transformants are therefore necessary for reliably obtaining multiple editings.

Reviewer #2: Minor errors
Line 161 and application > an application
Line 182 significantly less should be significantly fewer

A: All corrected.
Line 186. k.o. mutants is not an appropriate or precise description

A: ‚k.o mutants‘ replaced by ‚null mutants‘
Lines 196-197 ‘typical for error-prone NHEJ repair of CRISPR/Cas-induced DNA breaks’ – a reference is required here
A: We have inserted a reference.
Line 414 ‘up to >50%’ – up to 50% or always 50% or more?
A: Corrected to ‚up to 65%‘.
Line 562 Protoplasts > Protoplast
A: Corrected
Line 911 ‘colonies of an Asn mutant which’ – this is unclear – describe the mutation more precisely
A: Sentence was rephrased for clarity.

Reviewer #3:

Abstract, line 102-103: The term clean is vague, and the marker-less approach does not guarantee unwanted modifications.
A: ‚clean‘ was deleted.
Line 117: is the word ‘cultures’ a misspelling? Otherwise, the meaning is unclear.
A: ‚Cultures‘ replaced by ‚field and greenhouse crops‘
Line 142-143 descibes HR in the presence of donor sequence. I think it is appropriate to add language that the presence of homologous sequence can or may stimulate HDR, but it is not a given outcome.
A: This is correct, we have modified the sentence which now reads: ‚When a repair template (RT) DNA homologous to sequences flanking the break is provided, repair can also occur by homologous recombination (HR)‘
Line 418. Fungus, not fungi. Only a single other fungus is shown.
A: Correct, we have rephrased the sentence, which now reads: ‚Importantly, we could show that pTEL-mediated coediting also works with another filamentous fungus, indicating that it can be applied in many fungi‘
M&M 643-644 is not well described and not clear. Also, what is Cas9 Sod1-GFP fusion proteins?

 A: We have rephrased the sentence, which now reads: ‚The frozen powder was suspended in extraction buffer and centrifuged for 20 min at 4°C at 15,000 g. Fifteen µg of clear supernatant were separated on a native polyacrylamide gel. SOD activity was detected by inhibition of reduction of nitro blue tetrazolium by O_2_^-^. SOD-active areas became visible as clear zones on a blue-violet background.

PLOS authors have the option to publish the peer review history of their article ([what does this mean?](https://journals.plos.org/plospathogens/s/editorial-and-peer-review-process#loc-peer-review-history)). If published, this will include your full peer review and any attached files.


If you choose “no”, your identity will remain anonymous but your review may still be made public.


**Do you want your identity to be public for this peer review?** For information about this choice, including consent withdrawal, please see our [Privacy Policy](https://www.plos.org/privacy-policy).

Reviewer #1: No
Reviewer #2: No
Reviewer #3: No

Figure Files:

While revising your submission, please upload your figure files to the Preflight Analysis and Conversion Engine (PACE) digital diagnostic tool, [https://pacev2.apexcovantage.com](https://pacev2.apexcovantage.com/). PACE helps ensure that figures meet PLOS requirements. To use PACE, you must first register as a user. Then, login and navigate to the UPLOAD tab, where you will find detailed instructions on how to use the tool. If you encounter any issues or have any questions when using PACE, please email us at [figures@plos.org](mailto:figures@plos.org).

Data Requirements:

Please note that, as a condition of publication, PLOS' data policy requires that you make available all data used to draw the conclusions outlined in your manuscript. Data must be deposited in an appropriate repository, included within the body of the manuscript, or uploaded as supporting information. This includes all numerical values that were used to generate graphs, histograms etc.. For an example see here on PLOS Biology: <http://www.plosbiology.org/article/info%3Adoi%2F10.1371%2Fjournal.pbio.1001908#s5>.

Reproducibility:

To enhance the reproducibility of your results, PLOS recommends that you deposit laboratory protocols in protocols.io, where a protocol can be assigned its own identifier (DOI) such that it can be cited independently in the future. For instructions see [http://journals.plos.org/plospathogens/s/submission-guidelines#loc-materials-and-methods](http://journals.plos.org/plospathogens/s/submission-guidelines)

*In compliance with data protection regulations, you may request that we remove your personal registration details at any time.* [*(Remove my information/details)*](https://www.editorialmanager.com/ppathogens/login.asp?a=r)*. Please contact the publication office if you have any questions.*
