# Supplementary material for: CRISPR/Cas with ribonucleoprotein complexes and transiently selected telomere vectors allows highly efficient marker-free and multiple genome editing in Botrytis cinerea
Source: PLoS Pathog. 2020 Aug 17;16(8):e1008326. doi: 10.1371/journal.ppat.1008326 (PMC7451986; doi:10.1371/journal.ppat.1008326)
Supplement: Attachment — Submitted filename: Response to reviewers.docx [file ppat.1008326.s023.docx]

**Decision mail from PLoS Pathogens (3 July 2020) and answer (in red)**

Dear Dr. Hahn,

Thank you very much for submitting your manuscript "CRISPR/Cas with ribonucleoprotein complexes…“. The reviewers appreciated the attention to an important topic. Based on the reviews, we are likely to accept this manuscript for publication, providing that you modify the manuscript according to the review recommendations.

Please prepare and submit your revised manuscript within 30 days. When you are ready to resubmit, please upload the following:

[1] A letter containing a detailed list of your responses to all review comments, and a description of the changes you have made in the manuscript.

[2] Two versions of the revised manuscript: one with either highlights or tracked changes denoting where the text has been changed; the other a clean version (uploaded as the manuscript file).

Important additional instructions are given below your reviewer comments.

Thank you again for your submission to our journal. We hope that our editorial process has been constructive so far, and we welcome your feedback at any time. Please don't hesitate to contact us if you have any questions or comments.

Sincerely,

Melvin Bolton, Guest Editor, PLOS Pathogens

Bart Thomma, Section Editor, PLOS Pathogens

Kasturi Haldar, Editor-in-Chief, PLOS Pathogens

Michael Malim, Editor-in-Chief, PLOS Pathogens

***********************

Reviewer Comments (if any, and for reference):

Part I - Summary

Reviewer #3: The authors have addressed the major concerns from the previous version. There are more details provided for the methodology, and the results are more clearly presented. The usefulness of the approach is apparent and should aid in functional genomic studies in fungi.

Part II – Major Issues: Key Experiments Required for Acceptance

Reviewer #3: There is still a problem with using the term 'coediting', as detailed in Fig. 5A. There is only a single locus edited by Cas9, therefore it cannot be coedited. The authors acknowledged this point in their response, but there seem to be additional places in the manuscript where this term needs to be modified for accuracy.

A: We have changed the term ‚coediting‘ to ‚editing‘ in Fig. 5A. Otherwise, we did not use the term ‚coediting‘ anymore for pTEL-mediated editign. However, we still used -correctly- the term ‚coediting‘ correctly for bot2 boa6 and nep1 nep2 double mutagenesis and for the marker exchange experiments. In the discussion, we also changed ‚coediting‘ to ‚editing‘ in order to avoid misunderstandings.

We further corrected some typos and made some small changes in the text to improve clarity. In the Methods part (line 595), we have removed the statement ”(Leisen et al., unpublished)”, and replaced it by additional information.

We have also provided now a document (Suppl. File: raw data) containing all numerical values that were used to generate the graphs in the figures. We have also corrected some minor inconsistencies and errors in the new versions of the figures. All figures have now been checked by PACE.

Part III – Minor Issues: Editorial and Data Presentation Modifications

Reviewer #3: (No Response)

PLOS authors have the option to publish the peer review history of their article. If published, this will include your full peer review and any attached files.

A: We would like to take this option.

Figure Files:

While revising your submission, please upload your figure files to the Preflight Analysis and Conversion Engine (PACE) digital diagnostic tool, https://pacev2.apexcovantage.com. PACE helps ensure that figures meet PLOS requirements. To use PACE, you must first register as a user. Then, login and navigate to the UPLOAD tab, where you will find detailed instructions on how to use the tool. If you encounter any issues or have any questions when using PACE, please email us at figures@plos.org.

Data Requirements:

Please note that, as a condition of publication, PLOS' data policy requires that you make available all data used to draw the conclusions outlined in your manuscript. Data must be deposited in an appropriate repository, included within the body of the manuscript, or uploaded as supporting information. This includes all numerical values that were used to generate graphs, histograms etc..

A: We have submitted a file with raw data, see above.

Reproducibility:

To enhance the reproducibility of your results, PLOS recommends that you deposit laboratory protocols in protocols.io, where a protocol can be assigned its own identifier (DOI) such that it can be cited independently in the future. For instructions see http://journals.plos.org/plospathogens/s/submission-guidelines#loc-materials-and-methods
